# Transgene-free, virus-based gene silencing in plants by artificial microRNAs derived from minimal precursors

**DOI:** 10.1093/nar/gkad747

**Published:** 2023-09-15

**Authors:** Adriana E Cisneros, Tamara Martín-García, Anamarija Primc, Wojtek Kuziuta, Javier Sánchez-Vicente, Verónica Aragonés, José-Antonio Daròs, Alberto Carbonell

**Affiliations:** Instituto de Biología Molecular y Celular de Plantas (IBMCP), CSIC-Universitat Politècnica de València, Av. de los Naranjos s/n, 46022 Valencia, Spain; Instituto de Biología Molecular y Celular de Plantas (IBMCP), CSIC-Universitat Politècnica de València, Av. de los Naranjos s/n, 46022 Valencia, Spain; Instituto de Biología Molecular y Celular de Plantas (IBMCP), CSIC-Universitat Politècnica de València, Av. de los Naranjos s/n, 46022 Valencia, Spain; Instituto de Biología Molecular y Celular de Plantas (IBMCP), CSIC-Universitat Politècnica de València, Av. de los Naranjos s/n, 46022 Valencia, Spain; Instituto de Biología Molecular y Celular de Plantas (IBMCP), CSIC-Universitat Politècnica de València, Av. de los Naranjos s/n, 46022 Valencia, Spain; Instituto de Biología Molecular y Celular de Plantas (IBMCP), CSIC-Universitat Politècnica de València, Av. de los Naranjos s/n, 46022 Valencia, Spain; Instituto de Biología Molecular y Celular de Plantas (IBMCP), CSIC-Universitat Politècnica de València, Av. de los Naranjos s/n, 46022 Valencia, Spain; Instituto de Biología Molecular y Celular de Plantas (IBMCP), CSIC-Universitat Politècnica de València, Av. de los Naranjos s/n, 46022 Valencia, Spain

## Abstract

Artificial microRNAs (amiRNAs) are highly specific, 21-nucleotide (nt) small RNAs designed to silence target transcripts. In plants, their application as biotechnological tools for functional genomics or crop improvement is limited by the need of transgenically expressing long primary miRNA (pri-miRNA) precursors to produce the amiRNAs *in vivo*. Here, we analyzed the minimal structural and sequence requirements for producing effective amiRNAs from the widely used, 521-nt long *AtMIR390a* pri-miRNA from *Arabidopsis thaliana*. We functionally screened in *Nicotiana benthamiana* a large collection of constructs transiently expressing amiRNAs against endogenous genes and from artificially shortened *MIR390*-based precursors and concluded that highly effective and accurately processed amiRNAs can be produced from a chimeric precursor of only 89 nt. This minimal precursor was further validated in *A. thaliana* transgenic plants expressing amiRNAs against endogenous genes. Remarkably, minimal but not full-length precursors produce authentic amiRNAs and induce widespread gene silencing in *N. benthamiana* when expressed from an RNA virus, which can be applied into leaves by spraying infectious crude extracts. Our results reveal that the length of amiRNA precursors can be shortened without affecting silencing efficacy, and that viral vectors including minimal amiRNA precursors can be applied in a transgene-free manner to induce whole-plant gene silencing.

## Graphical Abstract

**Graphical Abstract:**
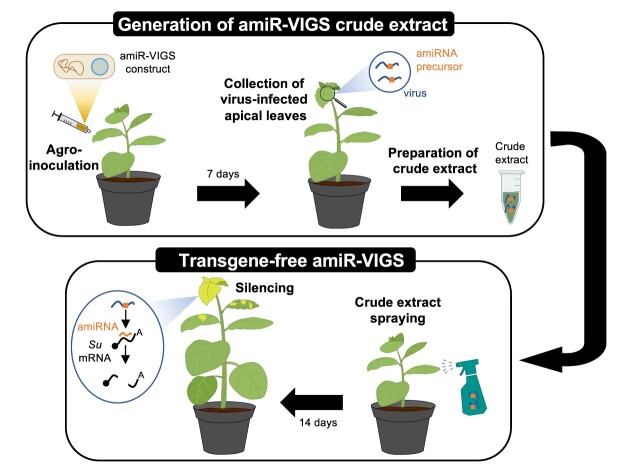

## INTRODUCTION

MicroRNAs (miRNAs) are a class of 20–24-nucleotide (nt) endogenous small RNAs (sRNAs) that repress gene expression post-transcriptionally in plants and animals. Most plant miRNAs are 21-nt long and regulate the expression of genes encoding transcription factors and proteins that impact key biological processes such as growth, development, stress adaptation or hormone regulation (1,2). MiRNA biogenesis in plants [reviewed recently in (3–7)] occurs entirely in the cell nucleus, and initiates with the transcription of *MIR* genes by RNA polymerase II, resulting in the synthesis of large primary miRNA precursors (or pri-miRNAs) that are highly variable in length (from hundreds to thousands of nucleotides) and in secondary structure. Pri-miRNAs are capped, spliced and polyadenylated, and harbor a characteristic foldback (or hairpin) structure that also varies in length (from 50 to 900 nt). MiRNA foldbacks have a common structure consisting of a ∼15–17 base-pair (bp) basal stem (BS), a miRNA/miRNA* duplex, and a distal stem–loop (DSL) region variable in size and shape and flanked by single-stranded RNA (ssRNA) segments (8,9). Processing of most pri-miRNAs occurs through a ‘base-to-loop’ mechanism in which the nuclear RNase III DICER-LIKE1 (DCL1) associated with its accessory proteins SERRATE (SE) and HYPONASTIC LEAVES1 (HYL1) first cleaves at the BS of the foldback to release a shorter precursor miRNA (pre-miRNA). Next, a second cleavage at a 21-nt distance from the first cut releases the miRNA/miRNA* duplex including two-nucleotide 3′ overhangs that are 2′-*O*-methylated by the methyltransferase HUA ENHANCER1 (HEN1) (3,10). Alternatively, for loop-to-base processed precursors, DCL1 produces a first cut that releases the terminal loop and then continues towards the base of the precursor to release the miRNA/miRNA* duplex (8,11). Typically, one of the strands of the duplex (the guide strand) is sorted out into an ARGONAUTE (AGO) protein (AGO1 for the majority of plant miRNAs) resulting in an RNA-induced silencing complex (RISC) that recognizes and silences complementary target RNAs mostly through their endonucleolytic cleavage, and less frequently through their translational repression (12).

Artificial miRNAs (amiRNAs) are 21-nt miRNAs computationally designed for redirecting the endogenous miRNA biogenesis and silencing machineries to selectively suppress specific target RNAs with high efficacy and specificity (with no predicted off-targets) (13–15). AmiRNAs are produced *in planta* by expressing a modified *MIR* gene producing a pri-miRNA precursor in which the sequence of the native miRNA/miRNA* duplex is substituted by the sequence of the amiRNA/amiRNA* duplex. The selection of an appropriate pri-miRNA backbone is critical for ensuring an efficient and accurate precursor processing and produce high amounts of amiRNAs necessary for effective target silencing. The 521-nt long pri*-*miRNA precursor derived from *Arabidopsis thaliana* (Arabidopsis) *MIR390a* (*pri-AtMIR390a*) is a highly efficient and accurately processed pri-miRNA compared to other plant pri-miRNAs used for producing amiRNAs (16,17). Moreover, *pri-AtMIR390a* has been used for expressing amiRNAs in multiple plant species including model and crop plants, to effectively silence both endogenous genes and viral RNAs (17–20). Interestingly, *pri-AtMIR390a* has a relatively short DSL region of only 31 nt, allowing the synthesis of the complete foldback with just two oligonucleotides spanning this structure. This feature has facilitated the development of a cost-effective, high-throughput methodology to directly introduce amiRNA inserts into a series of ‘B/c’ vectors including the *pri-AtMIR390a* sequence (16).

AmiRNAs are used as biotechnological tools for both functional genomics and crop improvement (19,21). However, a current limitation of the amiRNA technology is the use of relatively long precursors, mostly based on full-length pri-miRNA sequences, to produce the amiRNAs *in vivo*. Reducing the size of amiRNA precursors should have several biotechnological advantages such as a more cost-effective production of amiRNA constructs or a more efficient *in vitro* or in bacteria synthesis of amiRNA precursors for topical application to plants. Another limitation is the need to genetically modify plant genomes with amiRNA transgenes. The use of plant DNA viruses to successfully express authentic amiRNAs in *Nicotiana benthamiana* (22–24) is promising, although infections are typically triggered by the agroinfiltration of plant tissues with plasmids including the amiRNA-expressing viral vector and limited to a narrow list of host species. Therefore, this so-called amiRNA-based virus-induced gene silencing (amiR-VIGS) technology requires further optimization for its broader application in multiple plant species and to avoid the generation of genetically modified plant tissues.

Here, we further expanded our recently developed silencing sensor system in *N. benthamiana* (25) and used it to systematically analyze the minimal structural and sequence requirements for producing effective amiRNAs from the broadly used, 521-nt long *pri-AtMIR390a* precursor. We functionally screened a large collection of constructs expressing amiRNAs against the *N. benthamiana* magnesium chelatase subunit CHLI-encoding *SULPHUR* (*NbSu*) or the *1-DEOXY-D-XYLULOSE-5-PHOSPHATE SYNTHASE* (*NbDXS*) genes from *AtMIR390a*-based precursors with artificially shortened BS or DSL regions. By combining phenotypic, biochemical and molecular assays with high-throughput sequencing-based analysis of precursor processing we show that highly effective, accurately processed amiRNAs can be produced in *N. benthamiana* from a shortened chimeric *MIR390*-based amiRNA precursor of only 89 nt. This ‘minimal’ precursor includes the complete BS region of *pri-AtMIR390a* (with no additional ssRNA segments) and the DSL region derived from *Oryza sativa MIR390* (*OsMIR390*) with a 2-nt deletion. The precursor was further validated in Arabidopsis transgenic plants expressing amiRNAs for silencing *CHLORINE42* (*AtCH42*) or *FLOWERING LOCUS T* (*AtFT*) which resulted in bleaching and late flowering phenotypes, respectively. Finally, we show that the minimal precursor has the unique ability to produce authentic amiRNAs and trigger widespread gene silencing in *N. benthamiana* when expressed from an RNA virus vector sprayed to leaves in a non-transgenic, DNA-free manner.

MATERIALS AND METHODS

#### Plant materials and growth conditions

*N. benthamiana* and *Arabidopsis thaliana* (Arabidopsis) Col-0 plants were grown in a growth chamber at 25°C with a 12 h-light/12 h-dark photoperiod or at 22°C with a 16 h-light/8 h-dark photoperiod, respectively. *DCL1i* and *DCL4i N. benthamiana* knockdown plants were described previously (26). Arabidopsis Col-0 plants were genetically transformed using the floral dip method (27) with the *Agrobacterium tumefaciens* GV3101 strain. T1 transgenic Arabidopsis were grown as previously (28). Photographs were taken with a Nikon D3000 digital camera with AF-S DX NIKKOR 18–55 mm *f*/3.5–5.6G VR lens.

#### Plant phenotyping

Plant phenotyping analyses were conducted in blind as described (28). Briefly, the flowering time of each independent line is the number of days elapsed from seed plating to first bud opening (or ‘days to flowering’). The ‘CH42’ phenotype was scored in 10 days old seedlings and was reported as ‘weak’, ‘intermediate’ or ‘severe’ if seedlings had more than two leaves, exactly two leaves or no leaves at all (only two cotyledons), respectively.

#### Artificial small RNA design

AmiR-GUS_Nb_, amiR-GUS_At_, amiR-NbSu and amiR-AtCH42 amiRNA design was described before (25,28,29).

P-SAMS script (https://github.com/carringtonlab/p-sams) (30) returning unlimited optimal results was used to obtain the complete list of optimal amiRNAs targeting *NbDXS* with high specificity (Data S1). The off-targeting filtering in *N. benthamiana* transcriptome v5.1 (31) was enabled in the design to increase amiRNA specificity.

#### DNA constructs

Oligonucleotides AC-627 and AC-628 were annealed and ligated in to *pENTR-D-TOPO* to generate *pENTR-BS-AtMIR390a-BB* including the *BS* sequence interrupted by two inverted *Bsa*I restriction sites. The *B/c* cassette was excised from *Bsa*I-digested *pENTR-AtMIR390a-B/c* (Addgene plasmid #51778), (16) and ligated into *Bsa*I-digested *pENTR-BS-AtMIR390a-BB* to generated *pENTR-BS-AtMIR390a-B/c*. The *BS-AtMIR390a-BB* cassette from *pENTR-AtMIR390a-BB* was transferred by LR recombination into *pMDC32B* (16), a version of *pMDC32* (31,32) with mutated *Bsa*I site, to generate *pMDC32B-BS-AtMIR390a-BB*. Finally, the *B/c* cassette was excised from *Bsa*I-digested *pENTR-AtMIR390a-B/c* and ligated into *Bsa*I-digested *pMDC32B-BS-AtMIR390a-BB* to generate *pMDC32B-BS-AtMIR390a-B/c*. AmiRNA *pENTR-BS-AtMIR390a-B/c* (Addgene plasmid 199559) and *pMDC32B-BS-AtMIR390a-B/c* vectors (Addgene plasmid 199560) are available from Addgene at http://www.addgene.org/.

Constructs 35S:AtDSL-D6-amiR-NbSu, 35S:AtDSL-D13-amiR-NbSu, 35S:AtDSL-D21-amiR-NbSu, 35S:AtDSL-D25-amiR-NbSu, 35S:pri-amiR-NbDXS, 35S:AtDSL-D6-amiR-NbDXS, 35S:AtDSL-D13-amiR-NbDXS, 35S:AtDSL-D21-amiR-NbDXS, 35S:AtDSL-D25-amiR-NbDXS, 35S:OsDSL-amiR-NbSu, 35S:OsDSL-D2-amiR-NbSu, 35S:OsDSL-D4-amiR-NbSu, 35S:OsDSL-D6-amiR-NbSu, 35S:OsDS-AtL-amiR-NbSu, 35S:OsDSL-amiR-NbDXS, 35S:OsDSL-D2-amiR-NbDXS, 35S:OsDSL-D4-amiR-NbDXS, 35S:OsDSL-D6-amiR-NbDXS, 35S:OsDS-AtL-amiR-NbDXS were obtained by ligating annealed oligonucleotide pairs listed in Table S1 into pMDC32B-AtMIR390a-B/c (Addgene plasmid 51776) as described (16). BS cassettes from 35S:pri-amiR-NbSu (25) and 35S:pri-amiR-NbDXS were amplified with oligo pairs AC-335/AC336, inserted into pENTR-D-TOPO and transferred by LR recombination into pMDC32B to generate 35S:BS-amiR-NbSu and 35S:BS-amiR-NbDXS, respectively.

DsDNA oligonucleotides AC-558, AC-559, AC-560, AC-561, AC-611, AC-612, AC-613 and AC-14 were ligated into *pENTR-D-TOPO* to generate *pENTR-BS-D7-amiR-NbSu*, *pENTR-BS-D17-amiR-NbSu*, *pENTR-BS-D23-amiR-NbSu*, *pENTR-BS-D31-amiR-NbSu*, *pENTR-BS-D7-amiR-NbDXS*, *pENTR-BS-D17-amiR-NbDXS*, *pENTR-BS-D23-amiR-NbDXS*, and *pENTR-BS-D31-amiR-NbDXS*, respectively. *BS* cassettes were transferred by LR recombination to *pMDC32B* to generate *35S:BS-D7-amiR-NbSu*, *35S:BS-D17-amiR-NbSu*, *35S:BS-D23-amiR-NbSu*, *35S:BS-D31-amiR-NbSu*, *35S:BS-D7-amiR-NbDXS*, *35S:BS-D17-amiR-NbDXS*, *35S:BS-D23-amiR-NbDXS*, *35S:BS-D31-amiR-NbDXS*.

Constructs *35S:shc-amiR-NbSu*, *35S:shc-amiR-NbDXS*, *35S:shc-amiR-TSWV*, *35S:shc-amiR-AtCH42, 35S:shc-amiR-AtFT* were obtained by ligating annealed oligonucleotide pairs AC-486/AC-487, AC-603/AC-604, AC-672/AC-673, AC-623/AC-624 and AC-621/AC-622, respectively into *pMDC32B-BS-AtMIR390a-B/c* as described (Appendix S1) (33).

The potato virus X (PVX) vector consisted of a modified version of PVX-MT799816.1 (34). The PVX vector was cloned into *pLX-B2* (35) flanked by the 35S CaMV promoter and terminator and an hepatitis delta virus ribozyme (36), to produce *pLB-PVX-MluI*. For PVX-based amiRNA constructs, *pri-* and *shc*-based cassettes were amplified from *35S:pri-amiR-GUS_Nb_*/*35S:pri-amiR-NbSu* or *35S:shc-amiR-NbSu* constructs with oligonucleotides AC-648/AC-662 and AC-650/AC-663, respectively, gel purified and assembled into *Mlu*I-digested and gel-purified *pLB-PVX-MluI* in the presence of GeneArt Gibson Assembly HiFi Master Mix (Invitrogen) to generate *35S:PVX-pri-amiR-GUS_Nb_*, *35S:PVX-pri-amiR-NbSu* and *35S:PVX-shc-amiR-NbSu*. See Appendix S2 for a general protocol to generate PVX-based amiRNA constructs. All precursors were expressed from endogenous PVX coat protein (CP) promoter. An heterologous promoter derived from bamboo mosaic virus (BaMV) drives the expression of PVX CP, which contains the deletion of the 29 initial codons to enhance the stability of the recombinant clones (37). *35S:PVX* was obtained by digesting *pLB-PVX-Z* with *Mlu*I, gel purifying and re-ligating the backbone band. For *35S:PVX-NbSu(89)*, *NbSu(89)* cassette including 89 nucleotides from *NbSu* (positions 1004 to 1092 in reverse orientation) was amplified from *pLX-TRV2-CHLI* (38) with oligos AC-919/AC-921, and inserted in *pLB-PVX-MluI* as described above for amiRNA precursors.

AmiRNA constructs *35S:pri-amiR-GUS_Nb_* and *35S:pri-amiR-NbSu* were reported previously (25). All DNA oligonucleotides used for generating the constructs described above are listed in Table S1. The sequences of all amiRNA precursors are listed in Appendix S3. The sequences of the *BS-AtMIR390a*-based B/c amiRNA vectors are listed in Appendix S4.

#### Transient expression of constructs and mechanical inoculation of viruses

DNA constructs were agroinfiltrated in *N. benthamiana* leaves as described (39,40), using *A. tumefaciens* GV3101 strain. Infection assays with TSWV LL-N.05 isolate were done as described (41,42). Mechanical inoculation with crude extracts from PVX infected plants was done as described for TSWV.

#### Chlorophyll extraction and analysis

Pigments from *N. benthamiana* infiltrated leaf tissues were extracted as described (43). Absorbance measurements and content in chlorophyll *a* were calculated as described (28).

#### RNA preparation

Total RNA from *N. benthamiana* leaves or from Arabidopsis seedlings was isolated as follows. Tissue was ground in liquid nitrogen to fine powder and homogenized in extraction buffer (1 M guanidinium thiocyanate, 1 M ammonium thiocyanate, 0.1 M sodium acetate, 5% glycerol, 38% water-saturated phenol). Next, RNA was extracted by chloroform extraction and precipitated after adding 0.5 volumes of isopropanol for 20 min. Triplicate samples from pools of two *N. benthamiana* leaves or 9–12 Arabidopsis seedlings were analyzed.

#### Real-time RT-qPCR

cDNA was obtained from 500 ng of DNaseI-treated total RNA from apical leaves at 14 days-post agroinoculation (dpa), using the PrimeScript RT Reagent Kit (Perfect Real Time) (Takara) according to the manufacturer's instructions.

Real time RT-qPCR was done as described in a QuantStudio 3 Real-Time PCR system (Thermo Fisher Scientific). *NbSu*/*NbDXS/PVX* and *AtCH42* target RNA expression levels were calculated relative to *N. benthamiana PROTEIN PHOSPHATASE 2A* (*NbPP2A*) and Arabidopsis *ACTIN 2* (*AtACT2*) reference genes, respectively, using the delta delta cycle threshold comparative method of the QuantStudio Design and Analysis Software (version 1.5.1.; Thermo Fisher Scientific). Oligonucleotides used for RT-qPCR are listed in Table S1.

#### Small RNA blot assays

Small RNA blot assays and band quantification from radioactive membranes were done as described (25). Oligonucleotides used as probes for sRNA blots are listed in Table S1.

#### Small RNA sequencing and data analysis

Total RNA quantity, purity and integrity was analyzed with a 2100 Bioanalyzer (RNA 6000 Nano kit, Agilent) and submitted to BGI (Hong Kong, China) for sRNA library preparation and SE50 sequencing in a DNBSEQ-G-400 sequencer (BGI). After reception of quality-trimmed, adaptor removed clean reads from BGI, fastx_collapser (http://hannonlab.cshl.edu/fastx_toolkit) (44) was used to collapse identical reads into a single sequence, while maintaining read counts. A custom Python script was then used to map each clean, unique read against the forward strand of the amiRNA precursor overexpressed in each sample (Data S2) not allowing mismatches or gaps, and also to calculate the counts and RPMs (reads per million mapped reads) for each mapping position. Processing accuracy of amiRNA foldbacks was assessed by quantifying the proportion of 19–24 nt sRNA (+) reads that mapped within ± 4 nt of the 5′ end of the amiRNA guide as reported before (39,43). Phasing register tables were built by calculating the proportion of 21-nucleotide sRNA (+) reads in each register relative to the corresponding amiRNA cleavage site for all 21-nucleotide positions downstream of the cleavage site, as described previously (16). List of 21-nucleotide sRNA (+) reads of target RNAs and species-specific tasiRNA-generating controls (*AtTAS1c* in Arabidopsis and *AtTAS3* in *N. benthamiana*) are shown in Data S3.

#### Stability and sequence analyses of amiRNA precursors during viral infections

Total RNA from apical leaves of each of the three biological replicates was pooled before cDNA synthesis, which was performed as explained above. PCR to detect amiRNA precursors, PVX and *NPP2A* was performed using oligonucleotide pairs AC-654/AC-655, AC-657/AC-658 and AC-365/AC-366 (Table S1), respectively, and Phusion DNA polymerase (ThermoFisher Scientific). PCR products were analyzed by agarose gel electrophoresis, and products of the expected sized were excised from the gel and sequenced.

#### Preparation and spraying of crude extracts obtained from virus infected plants

For each plant, 1 g from 1–2 apical leaves was ground to fine powder, homogenized in 5 ml of buffer (50 mM potassium phosphate pH 8.0, 1% polyvinylpyrrolidone 10 and 1% polyethylene glycol 6000), filtered with sterile Miracloth (Millipore) and transferred to a 30-ml high-density polyethylene vaporizer (Yizhao). All volume (∼5 ml) was sprayed to two different leaves (third and fourth leaves counting from the bottom) of a 3-week old *N. benthamiana* plant, at a 5–10 cm distance (Supplementary Figure S1).

#### Gene and virus identifiers

Arabidopsis and *N. benthamiana* gene identifiers are *AtACT2* (AT3G18780), *AtCH42* (AT4G18480), *AtFT* (AT1G65480), *NbSu* (Nbv5.1tr6204879), *NbDXS* (Nbv5.1tr6224823) and *NbPP2A* (Nbv5.1tr6224808). TSWV LL-N.05 segment L, M and S genome identifiers are KP008128, FM163373 and KP008129, respectively. PVX sequence variant MT799816.1 was cloned into a derivative of *pLX-B2* (KY825137.1).

## RESULTS

### A silencing sensor system in *N. benthamiana* for easily visualizing and quantifying amiRNA-induced silencing of endogenous genes

**Figure 1.**
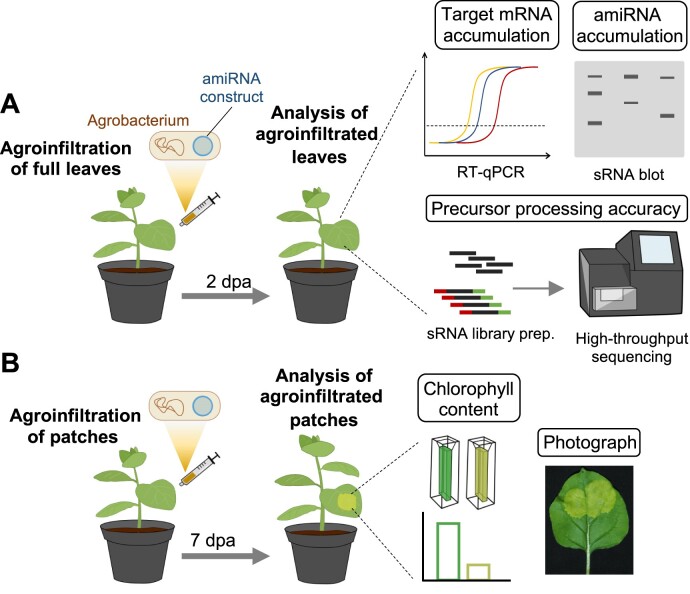
Methodology for easily visualizing and quantifying the silencing activity of amiRNAs derived from *AtMIR390a*-based precursors. (**A** and **B**) Steps for the analysis of fully agroinfiltrated leaves or patches, respectively.

**Figure 2.**
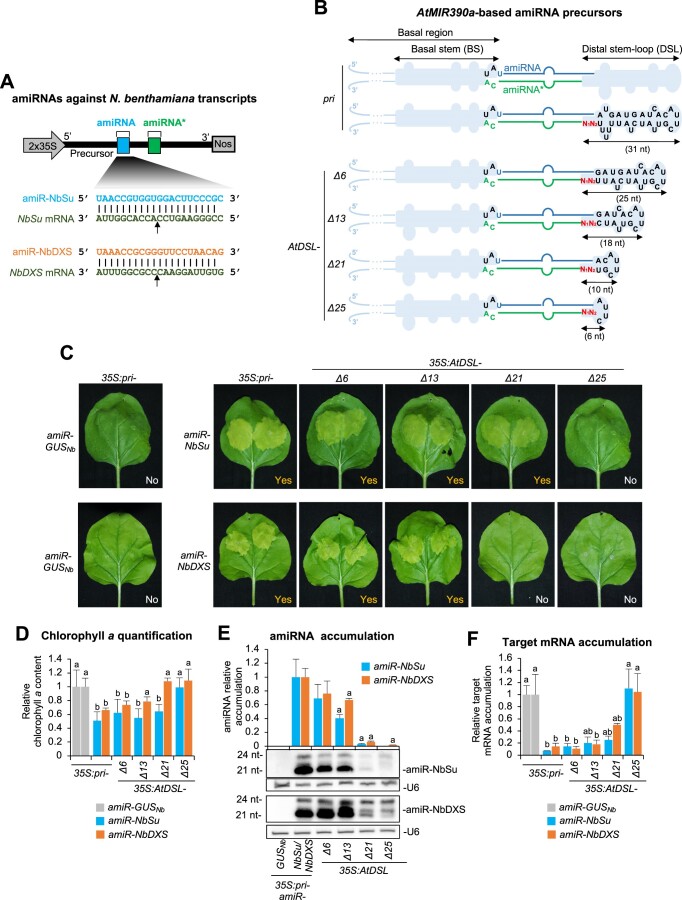
Functional analysis of constructs expressing amiR-NbSu or amiR-NbDXS against *N. benthamiana SULPHUR* (*NbSu*) or *DXS* (*NbDXS*), respectively, from *AtMIR390a*-based precursors with shortened distal stem–loop (DSL) region. (**A**) Diagram of *AtMIR390a*-based amiRNA constructs including the base-pairing of amiRNAs and target mRNAs. Nucleotides corresponding to the guide strand of the amiRNA against *NbSu* and *NbDXS* are in blue and orange, respectively, while nucleotides of target mRNAs are in dark green. The arrows indicate the amiRNA-predicted cleavage site. (**B**) Diagrams of the amiRNA precursors with nucleotides corresponding to the amiRNA guide and amiRNA* strands are in blue and green, respectively, while those from *AtMIR390a* are in black. The two nucleotides specific to each amiRNA construct that are modified for preserving authentic *pri* foldback secondary structure are in red. The shapes corresponding to *pri* basal stem (BS) or DSL regions are in light blue. The size in nucleotides of the DSL region of each precursor is given. (**C**) Photos at 7 days post-agroinfiltration (dpa) of leaves agroinfiltrated with different constructs. ‘Yes’ and ’No’ labels indicate the presence or absence of bleached patches, respectively. (**D**) Bar graph showing the relative content of chlorophyll *a* in patches agroinfiltrated with different constructs (*pri-amiR-GUS_Nb_* = 1.0). Bars with the letter ‘b’ or ‘a’ are significantly different from that of the corresponding control samples *pri-amiR-GUS_Nb_* or *pri-amiR-NbSu*/*pri-amiR-NbDXS*, respectively (*P* < 0.05 in pairwise Student's *t*-test comparisons). (**E**) Northern blot detection of amiR-NbSu and amiR-NbDXS in RNA preparations from agroinfiltrated leaves at 2 dpa. The graph at top shows the mean (*n* = 3) + standard deviation amiRNA relative accumulation (*pri-amiR-NbSu* = 1.0; *pri-amiR-NbDXS* = 1.0). Bars with a letter ‘a’ are significantly different from that of sample *pri-amiR-NbSu* or *pri-amiR-NbDXS*. One blot from three biological replicates is shown. (**F**) Target mRNA accumulation in agroinfiltrated leaves. Mean relative level (*n* = 3) + standard error of *NbSu* or *NbDXS* mRNAs after normalization to *PROTEIN PHOSPHATASE 2A* (*PP2A*), as determined by quantitative RT-PCR (qPCR) (*pri-amiR-GUS_Nb_* = 1.0 in all comparisons). Other details are as in (D).

We recently described a silencing sensor system for easily visualizing and quantifying silencing of the magnesium chelatase subunit CHLI [hereafter *SULPHUR* gene (*NbSu*)] in *N. benthamiana* by the amiRNA amiR-NbSu-1 (25). To further expand the use of the sensor system, we tested if we could trigger a similar visible bleaching with amiRNAs directed against the *1-DEOXY-D-XYLULOSE-5-PHOSPHATE SYNTHASE* (*NbDXS*) gene. Briefly, three different constructs expressing amiRNAs targeting *NbDXS* at unique sites and with no predicted off-targets (Supplementary Figure S2A, Data S1) were independently agroinfiltrated in two areas of two leaves from three different *N. benthamiana* plants, together with a negative control construct expressing an amiRNA against *Escherichia coli* b-glucuronidase GUS. At 7 dpa, areas agroinfiltrated with anti-*NbDXS* amiRNA constructs displayed a visually obvious bleaching predicted from *NbDXS* knockdown while areas expressing the control construct did not (Supplementary Figure S2B). The chlorophyll analysis of agroinfiltrated areas showed a similar decrease in chlorophyll content in bleached areas expressing anti-*NbDXS* amiRNAs compared to areas expressing the control construct (Supplementary Figure S2C). Next, two leaves of three different plants were independently agroinfiltrated in the whole leaf surface with each of the amiRNA constructs described above. sRNA blot assays of RNA preparations from agroinfiltrated leaves showed that all anti-*NbDXS* amiRNAs accumulated as single-size sRNA species, with amiR-NbDXS-1 accumulating to significantly higher levels than the other two amiRNAs (Supplementary Figure S2D). RT-qPCR analysis revealed that all samples expressing anti-*NbDXS* amiRNAs accumulated significantly lower levels of *NbDXS* mRNA than control samples (Supplementary Figure S2E). These results indicate that anti-*NbDXS* amiRNAs are highly active and induce a strong bleaching phenotype in the tissue where they are expressed. Thus, the methodology described in (25) and outlined in Figure 1 was used in this work to test the effect on *NbSu* and *NbDXS* silencing efficacy of amiR-NbSu-1 (hereafter amiR-NbSu) and amiR-NbDXS-1 (hereafter amiR-NbDXS), respectively (Figure 2A), produced from *pri-AtMIR390a* precursors with reduced BS or DSL regions (see next four sections).

### Functional analysis of *pri-AtMIR390a*-based amiRNA precursors with shortened DSL regions

First, we analyzed the silencing activity of amiR-NbSu- and amiR-NbDXS-expressing constructs including *pri-AtMIR390a*-based precursors with progressively shorter DSL regions ranging from 31 nt of the full-length *pri-AtMIR390a* (or simply ‘*pri*’) precursor to only 6 nt (Figure 2B). As expected, at 7 dpa areas expressing amiR-NbSu or amiR-NbDXS from the *pri* precursor displayed the reduced pigmentation characteristic of *NbSu* or *NbDXS* knock-down, while areas expressing amiR-GUS_Nb_ did not (Figure 2C). Interestingly, a similar bleaching was also observed in areas expressing amiR-NbSu or amiR-NbDXS from *AtDSL-Δ6* and *AtDSL-Δ13* precursors, while a less intense bleaching was noticed in areas expressing amiR-NbSu (but not amiR-NbDXS) from the *AtDSL-Δ21* precursor (Figure 2C). No bleaching was observed in areas expressing amiRNAs from the *AtDSL-Δ25* precursor (Figure 2C). Visual silencing phenotypes were confirmed by the chlorophyll analysis of agroinfiltrated areas, with bleached areas displaying lower chlorophyll *a* content than darker green areas (Figure 2D). RNA blot assays of RNA preparations from agroinfiltrated leaves showed that amiR-NbSu and amiR-NbDXS accumulated to high levels when expressed from *pri* or from shorter *AtDSL-Δ6* and *AtDSL-Δ13* precursors (Figure 2E). In overexposed blots both amiRNAs were also detected although to low levels when expressed from *AtDSL-Δ21* and also from *AtDSL-Δ25* in the case of amiR-NbDXS (Figure 2E). Small RNA species of different sizes were also detected in some cases, most likely reflecting that the position of DCL1 cleavage is not always uniform, as proposed before (28). Twenty-four-nt sRNAs present in the overexposed blots presumably arise from transgene-derived dsRNAs, which typically occurs in *N. benthamiana* when agroinfiltrating ectopic transgene constructs, as observed before (45). Finally, RT-qPCR analysis revealed that target mRNA expression was considerably reduced in samples expressing amiRNAs from *pri* or from shortened precursors compared to control samples, except for samples expressing amiR-NbSu or amiR-NbDXS from *AtDSL-Δ25* (Figure 2F). Notably, samples expressing amiRNAs from *pri* or from *AtDSL-Δ6* accumulated similar low target mRNA levels, while the expression of shorter *AtDSL-Δ13* and *AtDSL-Δ21* precursors generally induced a more moderate reduction in target mRNA levels (Figure 2F). In contrast, no significant reduction in target mRNA levels was observed when amiRNAs were expressed from *AtDSL-Δ25* (Figure 2F). These results indicate that the DSL region of *pri-AtMIR390a* can be shortened to some extent without significantly affecting amiRNA accumulation and target mRNA silencing.

### AmiRNAs produced from *pri-AtMIR390a*-based precursors including shortened *OsMIR390*-derived DSL regions trigger gene silencing with high efficacy

**Figure 3.**
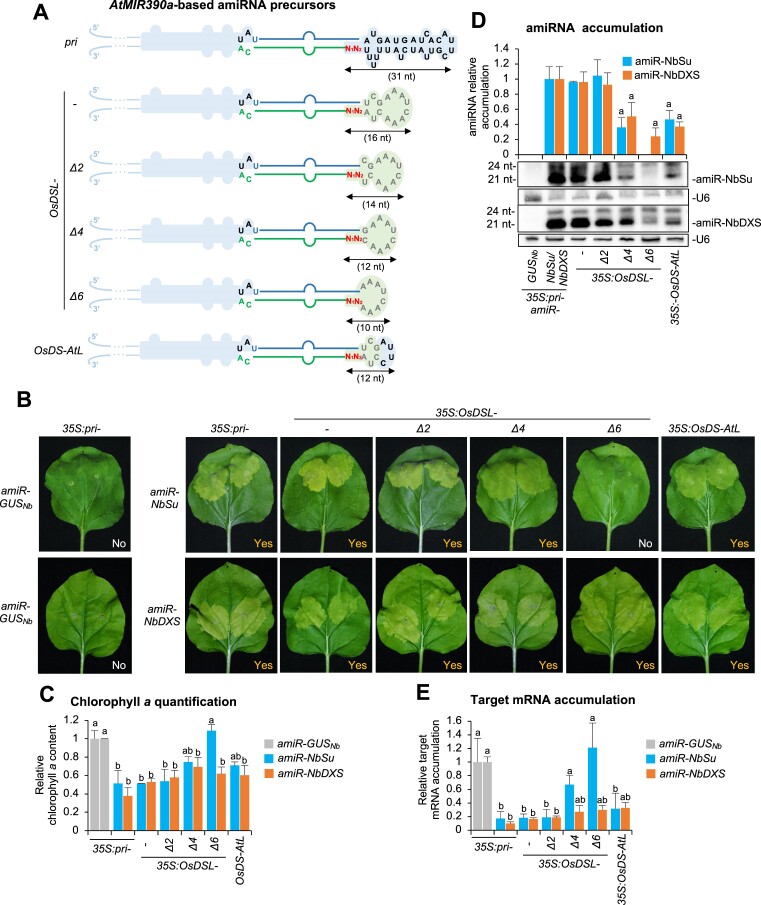
Functional analysis of constructs expressing amiR-NbSu or amiR-NbDXS against *N. benthamiana SULPHUR* (*NbSu*) or *DXS* (*NbDXS*) from chimeric precursors including intact *AtMIR390a* basal region and shortened *OsMIR390* distal stem–loop (DSL) region. (**A**) Diagrams of the amiRNA precursors. Nucleotides from *OsMIR390* are in grey. The shapes corresponding to *OsMIR390a* distal stem–loop regions are in light green. Other details are as in Figure 2B. (**B**) Photos at 7 days post-agroinfiltration (dpa) of leaves agroinfiltrated with different constructs. (**C**) Bar graph showing the relative content of chlorophyll *a* in patches agroinfiltrated with different constructs (*pri-amiR-GUS_Nb_* = 1.0). Other details are as in Figure 2D. (**D**) Northern blot detection of amiR-NbSu and amiR-NbDXS amiRNAs in RNA preparations from agroinfiltrated leaves at 2 dpa. Other details are as in Figures 2D and E. (**E**) Target mRNA accumulation in agroinfiltrated leaves. Other details are as in Figures 2D and F.

The *OsMIR390* precursor has a DSL region (OsDSL) consisting of only 16 nt, which represents one of the shortest DSL regions from all conserved plant *MIRNA* precursors (43). Here, we reasoned that the *OsDSL* region might also tolerate nucleotide deletions while preserving efficient amiRNA production and target mRNA silencing. To test this, several constructs were generated for expressing amiR-NbSu and amiR-NbDXS from chimeric constructs including the complete (16-nt long) *OsDSL* region or progressively shortened versions of it (Figure 3A). In addition, a construct in which the 8-nt terminal loop of *OsMIR390* was replaced by the shorter 4-nt long terminal loop of *AtMIR390a* was also included in the analysis (Figure 3A). Areas agroinfiltrated with constructs expressing amiRNAs from *pri* or from chimeric *OsDSL* and *OsDSL-D2* precursors showed intense bleaching and similar reduction in the accumulation of chlorophyll *a* compared to controls (Figure 3B and 2C). In contrast, other agroinfiltrated areas displayed weaker bleaching and lower chlorophyll *a* content except those from controls and from areas expressing amiR-NbSu from *OsDSL-D6* which did not display any sign of silencing (Figure 3B and C). RNA blot analysis of RNA preparations from agroinfiltrated leaves revealed that amiRNAs accumulated to high levels in samples expressing the *pri* or chimeric *OsDSL* and *OsDSL-D2* precursors, while amiRNA levels dropped significantly in the rest of samples except for those expressing the *35S:OsDSL-D6-amiR-NbSu* construct in which amiR-NbSu was not detected (Figure 3D). RT-qPCR analysis revealed that target mRNA accumulation significantly decreased in all samples expressing amiRNAs from *pri* or from chimeric precursors compared to controls, except in samples expressing amiR-NbSu from *OsDSL-D4* and *OsDSL-D6* (Figure 3E). Importantly, target mRNA levels were similar in samples expressing amiRNAs from *pri* or from chimeric *OsDSL* and *OsDSL-D2* precursors (Figure 3E). Taken together, these results indicate that the *OsDSL* region can also be shortened—in this case down to 14 nt—in *AtMIR390a*-based precursors without compromising silencing efficacy.

### Functional analysis of *AtMIR390a*-based amiRNA precursors with shortened BS

**Figure 4.**
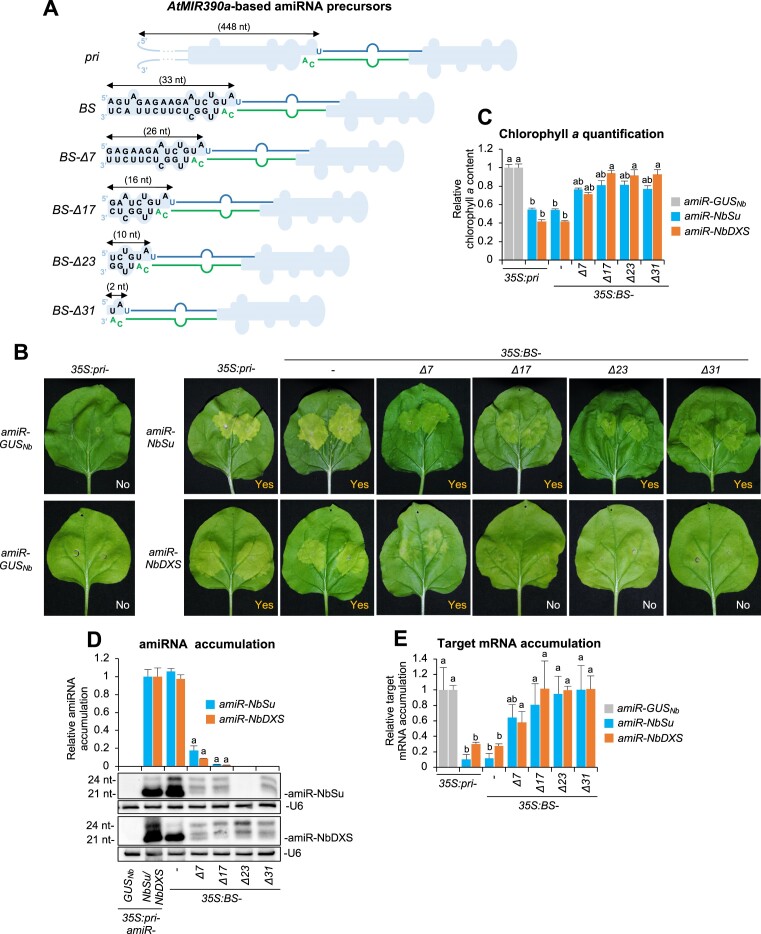
Functional analysis of constructs expressing the amiR-NbSu amiRNA against *N. benthamiana SULPHUR* (*NbSu*) or *DXS* (*NbDXS*) from *AtMIR390a* precursors with shortened basal stem (BS) region. (**A**) Diagrams of the amiRNA precursors. The size in nucleotides of the BS region of each precursor is given. Other details are as in Figure 2B. (**B**) Photos at 7 days post-agroinfiltration (dpa) of leaves agroinfiltrated with different constructs. (**C**) Bar graph showing the relative content of chlorophyll *a* in patches agroinfiltrated with different constructs (*pri-amiR-GUS_Nb_* = 1.0). Other details are as in Figure 2D. (**D**) Northern blot detection of amiR-NbSu and amiR-NbDXS amiRNAs in RNA preparations from agroinfiltrated leaves at 2 dpa. Other details are as in Figures 2D and E. (**E**) Target mRNA accumulation in agroinfiltrated leaves. Other details are as in Figures 2D and F.

Next, we analyzed if shortening the BS region of *pri-AtMIR390a* could affect amiRNA production and silencing efficacy. Several constructs for expressing amiR-NbSu and amiR-NbDXS were generated, in which the 448-nt BS region of *pri-AtMIR390a* was progressively shortened down to 2 nt (Figure 4A). Similar intense bleaching and reduced chlorophyll *a* level was observed in areas expressing amiR-NbSu or amiR-NbDXS from the *pri* or *BS* precursors (Figures 4B and 3C). In contrast, weaker bleaching was observed in areas expressing any of the two amiRNAs from *BS-D7*, or amiR-NbSu from *BS-D17*, *BS-D23* or *BS-31* (Figure 4B), corresponding with slight decreases in chlorophyll *a* content compared to controls (Figure 4C). No bleaching or changes in chlorophyll *a* content were observed in leaves expressing amiR-NbDXS from *BS-D17*, *BS-D23* or *BS-31* (Figure 4B and C). Both amiR-NbSu and amiR-NbDXS amiRNAs expressed from the *pri* or *BS* precursors accumulated to similarly high levels and induced comparable target mRNA silencing (Figure 4D and E). In contrast, their levels dropped significantly or were undetectable when expressed from *BS-D7*/*BS-D17* or *BS-D23/BS-D31*, respectively (Figure 4D) and induced significantly lower target mRNA downregulation (Figure 4E). All together these results indicate that a complete BS region is necessary for accurate precursor processing to produce high levels of amiRNAs, while further shortening this BS region drastically reduces amiRNA accumulation.

### New high-throughput vectors for expressing amiRNAs from *AtMIR390a*-based precursors only including the BS

Previous high-throughput *AtMIR390a*-based amiRNA ‘B/c’ vectors included the complete 521 bp sequence of the *pri-AtMIR390a* precursor (16). Here, we developed two additional ‘B/c’ vectors for the efficient cloning and expression of amiRNAs from shortened precursors only including *AtMIR390a* BS (Supplementary Figure S3). *pENTR-BS-AtMIR390a-B/c* Gateway-compatible entry vector was generated for direct cloning of amiRNA inserts and subsequent recombination into the preferred Gateway expression vector including the regulatory features of choice. *pMDC32B-BS-AtMIR390a-B/c* vector was generated for the direct cloning of amiRNA inserts into a binary expression vector. Both vectors contain a truncated *AtMIR390a* BS sequence including a 1461-bp DNA cassette with the control of cell death (*ccd*B) gene flanked by two inverted *Bsa*I sites downstream the BS region. The generation of an amiRNA construct with these two new B/c vectors is similar to that described for previous *AtMIR390a-B/c*-based vectors (16) (Supplementary Figure S4 and Appendix S1).

### Transient expression of amiRNAs from shortened, *MIR390*-based chimeric precursors triggers effective silencing of *N. benthamiana* endogenous genes and of pathogenic viral RNAs

**Figure 5.**
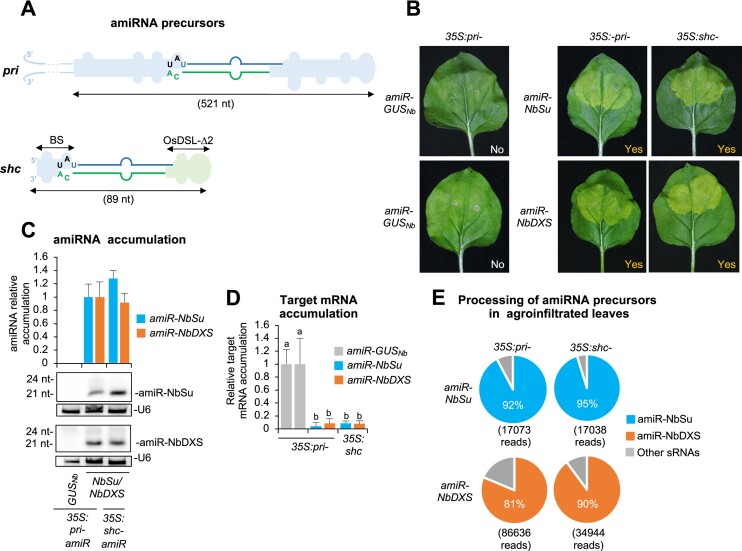
Functional analysis of constructs expressing the amiR-NbSu amiRNA against *N. benthamiana SULPHUR* (*NbSu*) or *DXS* (*NbDXS*) from *pri* and *shc* precursors. (**A**) Diagrams of the *pri* and *shc* amiRNA precursors. The total length of each precursor is indicated. Other details are as in Figure 2B. (**B**) Photos at 7 days post-agroinfiltration (dpa) of leaves agroinfiltrated with the two constructs. (**C**) Northern blot detection of amiR-NbSu and amiR-NbDXS amiRNAs in RNA preparations from agroinfiltrated leaves at 2 dpa. Other details are as in Figures 2D and E. (**D**) Target mRNA accumulation in agroinfiltrated leaves. Other details are as in Figures 2D and F. (**E**) amiRNA processing from *AtMIR390a*-based precursors. Pie charts show percentages of reads corresponding to expected, accurately processed 21-nucleotide mature amiR-NbSu and amiR-NbDXS (blue or orange sections, respectively) or to other 19–24-nucleotide sRNAs (gray sectors).

Because our previous results showed that the efficiency of amiRNA production and induced silencing of *N. benthamiana* endogenous genes were maintained in shortened precursors including the *BS* or *OsMIR390-D2* regions, the new ‘B/c’ vectors were used to systemically test the effect of combining both regions in a single precursor. The resulting shortened chimeric *MIR390-*based precursor of only 89 nt was named *shc-MIR390* precursor (or simply ‘*shc*’) (Figure 5A). First, we introduced into *pMDC32B-BS-AtMIR390a-B/c* the sequence corresponding to amiR-NbSu or amiR-NbDXS including the *OsMIR390-D2* region to generate the *35S:shc-amiR-NbSu* or *35S:shc-amiR-NbDXS* constructs, respectively. Both constructs were independently agroinfiltrated into *N. benthamiana* leaves together with control constructs, and induced similar strong bleaching, amiRNA accumulation and target mRNA downregulation in agroinfiltrated areas than their respective controls expressing these same amiRNAs from the full-length *pri* precursor (Figure 5B–D). To confirm the correct processing of *shc* precursors and compare it to that of the *pri*, sRNA libraries from leaves expressing *35S:pri-amiR-NbSu*, *35S:pri-amiR-NbDXS*, *35S:shc-amiR-NbSu* and *35S:shc-amiR-NbDXS* were prepared and sequenced. Interestingly, in samples expressing *35S:shc-amiR-NbSu* or *35S:shc-amiR-NbDXS*, the majority (95% and 90% respectively) of 19–24-nt (+) reads mapping within ±4 nt of the 5′ end of the amiRNA guide corresponded to authentic 21-nt amiR-NbSu or amiR-NbDXS (Figure 5E). These results indicate a high accuracy in the processing of *shc* precursors which was similar to that observed for the *pri* precursors (Figure 5E, Figure S5).

Next, we aimed to test if amiRNAs produced from the *shc* precursor could also be applied to silence exogenous transcripts, such as viral RNAs. Here, the sequence corresponding to the most efficient anti-tomato spotted wilt virus (TSWV) amiRNA (amiR-TSWV) identified in previous amiRNA screenings (41) was expressed from the *shc* precursor in a *N. benthamiana* leaf inoculated two days later with TSWV (Supplementary Figure S6A). At 7 days post-inoculation (dpi), all leaves inoculated with TSWV and expressing amiR-TSWV either from the *pri* or *shc* precursors did not display virus-induced symptoms, while leaves inoculated with TSWV and expressing the control amiR-GUS_Nb_ displayed multiple local lesions typical of TSWV infection (Supplementary Figure S6B). AmiRNA accumulation analyzed by sRNA northern blot showed that amiR-TSWV accumulated to similar levels in tissues expressing *35S:shc-amiR-TSWV* or *35S:pri-amiR-TSWV* (Supplementary Figure S6C). At 20 dpi, TSWV RNA was not detected in upper non-inoculated leaves from plants inoculated with TSWV and expressing *35S:shc-amiR-TSWV* or *35S:pri-amiR-TSWV*, while plants expressing *35S:pri-amiR-GUS_Nb_* and inoculated with TSWV accumulated high levels of TSWV RNA (Supplementary Figure S6C). Thus, these results indicate that the *shc* precursor can be used to express high levels of amiRNAs to induce antiviral resistance in plants.

### Stable expression of amiRNAs from *shc-MIR390* precursors leads to effective silencing of Arabidopsis endogenous genes

**Figure 6.**
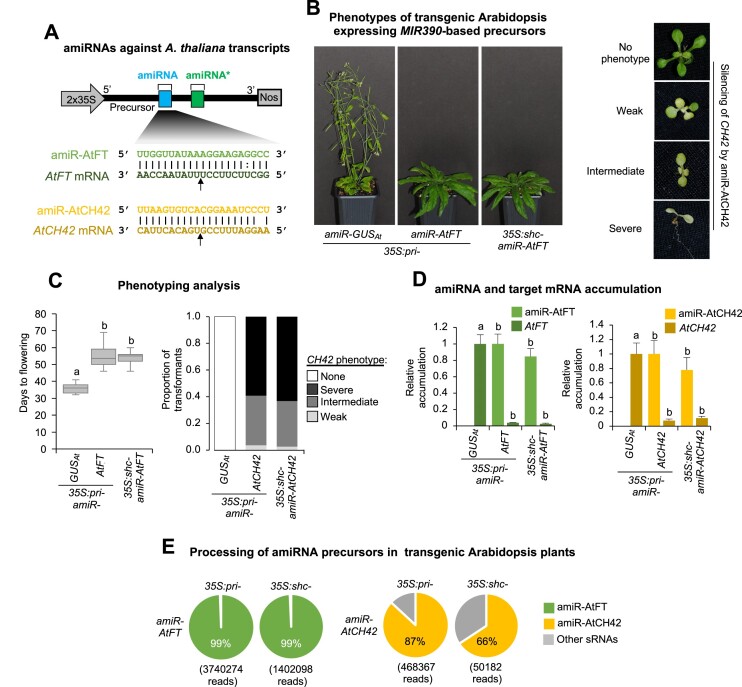
Functional analysis of constructs expressing the amiR-AtFT and amiR-AtCH42 amiRNAs against *A. thaliana FLOWERING LOCUS T* (*AtFT*) or *CHLORINE 42* (*AtCH42*) from *pri* and *shc* precursors. (**A**) Nucleotides corresponding to the guide strand of the amiRNA against *AtFT* and *AtCH42* are in light green and light yellow, respectively, while nucleotides of *AtFT* and *AtCH42* target mRNAs are in dark green and dark yellow, respectively. Other details are as in Figure 2A. (**B**) Representative images of Arabidopsis T1 transgenic plants expressing amiRNAs from different precursors. Left, 45-day-old adult plants expressing amiR-GUS_At_ or amiR-AtFT. Right, 10-day-old T1 seedlings expressing amiR-AtCH42 and showing bleaching phenotypes of diverse degrees. (**C**) Phenotyping analysis. Left, box plot representing the mean flowering time of Arabidopsis T1 transgenic plants expressing amiR-GUS_At_ or amiR-AtFT from different precursors. Pairwise Student's *t*-test comparisons are represented with the letter ‘a’ if significantly different (*P* < 0.05) and the letter ‘b’ if not (*P* > 0.05). Right, bar graph representing, for each line, the proportion of seedlings displaying a severe (black areas), intermediate (dark gray areas), or weak (light gray areas) bleaching phenotype, or with wild-type appearance (white areas). (**D**) amiRNA and target RNA accumulation. Left, bar graph showing the relative accumulation of amiR-AtFT [mean relative level (*n* = 3) + standard deviation amiRNA relative accumulation, *pri-amiR-AtFT* = 1.0] and of *AtFT* mRNA [mean relative level (*n* = 3) + standard error of *AtFT* mRNAs after normalization to *ACTIN 2* (*AtACT2*), as determined by quantitative RT-qPCR, *pri-amiR-GUS_At_* = 1]. Right, bar graph showing the relative accumulation of amiR-AtCH42 [mean relative level (*n* = 3) + standard deviation amiRNA relative accumulation, *pri-amiR-AtCH42* = 1.0] and of *AtCH42* mRNA [mean relative level (n = 3) + standard error of *AtCH42* mRNAs after normalization to *ACTIN 2* (*AtACT2*), as determined by quantitative RT-qPCR, *pri-amiR-GUS_At_* = 1]. (**E**) amiRNA processing from *pri* or *shc* precursors. Pie charts show percentages of reads corresponding to expected, accurately processed 21-nucleotide mature amiR-AtFT and amiR-AtCH42 (green or yellow sections, respectively) or to other 19–24-nucleotide sRNAs (gray sectors).

Next, we aimed to further confirm the functionality of *shc* precursors in a different plant species and when stably expressed in transgenic plants. For that purpose we used the amiR-AtFT/*AtFT* and amiR-AtCH42/*AtCH42* silencing sensor systems in Arabidopsis in which the transgenic expression of amiR-AtFT or amiR-AtCH42 from *pri-AtMIR390a* leads to a significant delay in flowering time or to an intense bleaching due to the targeting of endogenous *FLOWERING LOCUS T* (*AtFT*) or *CHLORINA 42* (*AtCH42*) endogenous genes, respectively (16). Here, we introduced amiR-AtFT and amiR-CH42 into *pMDC32B-BS-AtMIR390a-B/c* to generate the *35S:shc-amiR-AtFT* and *35S:shc-amiR-AtCH42* constructs, respectively (Figure 6A). These constructs were transformed independently into Arabidopsis Col-0 plants together with control constructs *35S:pri-amiR-GUS_Ath_*, *35S:pri-amiR-AtFT* and *35S:pri-amiR-AtCH42* expressing an amiRNA against GUS (with no predicted off-targets in Arabidopsis), *AtFT* and *AtCH42*, respectively, from *pri-AtMIR390a*. To systemically compare the processing and induced silencing of amiR-AtFT or amiR-AtCH42 produced from *shc* and *pri* precursors, plant phenotypes, amiRNA and target mRNA accumulation and processing efficiency were measured in Arabidopsis T1 transgenic lines.

All *35S:pri-amiR-AtFT* (*n* = 40) and *35S:shc-amiR-AtFT* (*n* = 34) transformants flowered later than the average flowering time of the *35S:pri-amiR-GUS_Ath_* control lines (*n* = 48) (Figure 6B, Table S2), and their average flowering time (54.7 ± 6 and 54.4 ± 3.2, respectively) was not significantly different between each other (Figure 6C). RNA-blot and RT-qPCR assays revealed that both amiR-AtFT and *AtFT* mRNA accumulation was similar in lines expressing amiR-AtFT from each of the two precursors (Figure 6D), and high-throughput sequencing of sRNAs showed that both precursors were efficiently processed, as in both cases 99% of the reads corresponded to authentic amiR-AtFT (Figure 6E, Figure S7). Similarly, all *35S:pri-amiR-AtCH42* (*n* = 54) and *35S:shc-amiR-AtCH42* (*n* = 38) transformants displayed bleaching of comparable degrees (Figure 6B and C; Table S2), accumulated similar levels of amiR-CH42 and AtCH42 mRNA (Figure 6D), and displayed effective precursor processing (Figure 6E, Figure S7). Taken together these results indicate that the *shc* precursor is functionally equivalent to the *pri* precursor in inducing highly effective silencing of endogenous genes when stably expressed in Arabidopsis.

### Unique functionality of the *shc* precursor in triggering widespread gene silencing in *N. benthamiana* when expressed from a viral vector

**Figure 7.**
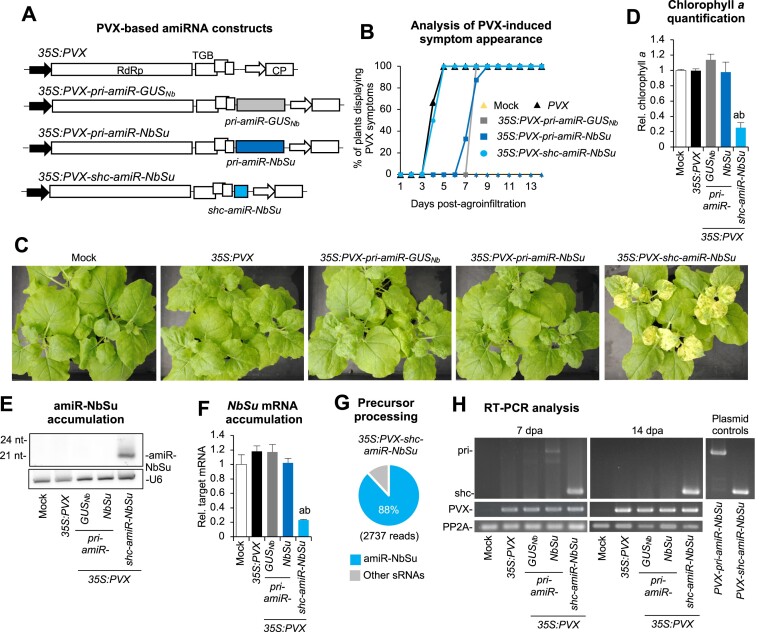
Functional analysis of *Potato virus X* (PVX) constructs expressing amiRNAs from *pri* and *shc* precursors. (**A**) Diagram of PVX-based constructs. *pri-amiR-GUS_Nb_*, *pri-amiR-NbSu* and *shc-amiR-NbSu* cassettes are shown in grey, dark blue and light blue boxes, respectively. PVX genes RdRp, TGB and CP are represented in white boxes, and CP promoter from *Bamboo mosaic virus* (BaMV) with a white arrow. (**B**) Two-dimensional line graph showing, for each of the three-plant sets listed, the percentage of symptomatic plants per day during 14 days. (**C**) Photos at 14 days post-agroinfiltration (dpa) of sets of three plants agroinfiltrated with the different constructs. (**D**) Bar graph showing the relative content of chlorophyll *a* in apical leaves from plants agroinfiltrated with different constructs (Mock = 1.0). Bar with the letter ‘a’ are significantly different from that of the corresponding mock control samples (*P* < 0.05 in pairwise Student's *t*-test comparison). (**E**) Northern blot detection of amiR-NbSu in RNA preparations from apical leaves collected at 14 dpa and pooled from three independent plants. (**F**) Target *NbSu* mRNA accumulation in RNA preparations from apical leaves collected at 14 dpa and analyzed individually. Mean relative level (*n* = 3) + standard error of *NbSu* mRNAs after normalization to *PROTEIN PHOSPHATASE 2A* (*PP2A*), as determined by RT-qPCR (mock = 1.0 in all comparisons). Other details are as in D. (**G**) amiR-NbSu processing from the *shc* precursor included in PVX (*35S:PVX-shc-amiR-NbSu* construct). Pie chart show percentages of reads corresponding to expected, accurately processed 21-nucleotide mature amiR-NbSu (blue sections) or to other 19–24-nucleotide sRNAs (gray sectors). (**H**) RT-PCR detection of PVX, *pri* and *shc* precursors in apical leaves at 7 and 14 dpa. RT-PCR products corresponding to the control *NbPP2A* are also shown (bottom), as well as control amplifications of *pri* and *shc* fragments from plasmids.

Because cargo capacity of viral vectors is limited, and large inserts are typically eliminated during viral replication in VIGS vectors (46), we hypothesized that the short (89-nt long) *shc* precursor fragment could be a more stable insert when included into a viral vector than the longer 521-nt *pri* precursor. To test this, *pri-amiR-NbSu* and *shc-amiR-NbSu* sequences were inserted into a PVX infectious clone to generate the *35S:PVX-pri-amiR-NbSu* and *35S:PVX-shc-amiR-NbSu* constructs, respectively (Figure 7A). These constructs were expected to express amiR-NbSu from *pri* and *shc* precursors, respectively, during PVX infection and induce the bleaching of PVX-infected tissues. In addition, the *35S:PVX-pri-amiR-GUS_Nb_* negative control construct was also generated. These three constructs together with the insert-free *35S:PVX* construct (Figure 7A) were independently agroinoculated in one leaf of three *N. benthamiana* plants. A ‘mock’ set of plants was agroinfiltrated with the agroinfiltration solution.

Mild leaf curling typical of PVX-induced symptoms was observed in upper non-agroinoculated leaves between 4 and 5 dpa in all plants agroinoculated with the *35S:PVX* and *35S:PVX-shc-amiR-NbSu* constructs (Figure 7B). However, curling in apical leaves from plants agroinoculated with the *35S:PVX-pri-amiR-GUS_Nb_* and *35S:PVX-pri-amiR-NbSu* constructs appeared 3–4 days later, between 7 and 9 dpa (Figure 7B). Interestingly, bleaching of apical leaves appeared at 8–9 dpa only in plants agroinoculated with the *35S:PVX-shc-amiR-NbSu* construct, and at 14 dpa extended to most of the apical tissues of infected plants (Figure 7C). Additional analyses confirmed the decrease in chlorophyll *a* level in bleached tissues (Figure 7D). Plants were monitored until 28 dpa, and only those expressing the *35S:PVX-shc-amiR-NbSu* construct displayed the characteristic yellowing derived from *NbSu* silencing. RNA-blot and RT-qPCR analyses showed that only tissues from plants expressing *35S:PVX-shc-amiR-NbSu* accumulated high levels of amiR-NbSu (Figure 7E) and low levels of *NbSu* mRNA (Figure 7F). Interestingly, sRNA sequencing from apical leaves of plants agroinoculated with *35S:PVX-shc-amiR-NbSu* revealed that the shc-amiR-NbSu precursor inserted into PVX was accurately processed (Figure 7G, Figure S8). Additional analyses showed that the percentages of sRNAs of (+) or (–) polarity derived from the PVX sequence are similar (48% and 52%, respectively), suggesting that these sRNAs originate from double-stranded RNA (dsRNA) intermediates produced by RNA-dependent RNA polymerases during PVX replication (Supplementary Figure S9). In contrast, the majority (89%) of sRNAs derived from the shc-amiR-NbSu sequence included in PVX are of (+) polarity, similarly to when the same precursor is expressed without PVX (Supplementary Figure S9). These results most likely indicate that amiR-NbSu originates from the *shc* precursor rather than from dsRNA intermediates of replication.

Finally, the presence of both *pri* and *shc* precursors in apical leaves was analyzed by RT-PCR at 7 and 14 dpa with oligonucleotides flanking the precursor insertion site. At 7 dpa, the *shc* precursor was clearly amplified in plants expressing *35S:PVX-shc-amiR-NbSu*, while only the *pri* precursor of the *35S:PVX-pri-amiR-NbSu* samples was barely detected (Figure 7H). Indeed, at 14 dpa only the *shc* precursor was detected (Figure 7H). Importantly, PVX was detected in all plants expressing PVX-based constructs at the two timepoints analyzed (Figure 7H) thus excluding the possibility that the absence of *pri* precursors was due to a lack of infection in these samples. Sequencing analysis of RT-PCR products from each of the three *PVX-shc-amiR-NbSu* infected samples showed that no mutations accumulated in the *shc-amiR-NbSu* insert. These results indicate that the *pri* fragments are eliminated from the PVX vector during viral infection, while the shorter *shc* fragments are stable both structurally and at a sequence level.

### A PVX-based, DNA-free system for inducing widespread silencing in plants with amiRNAs

**Figure 8.**
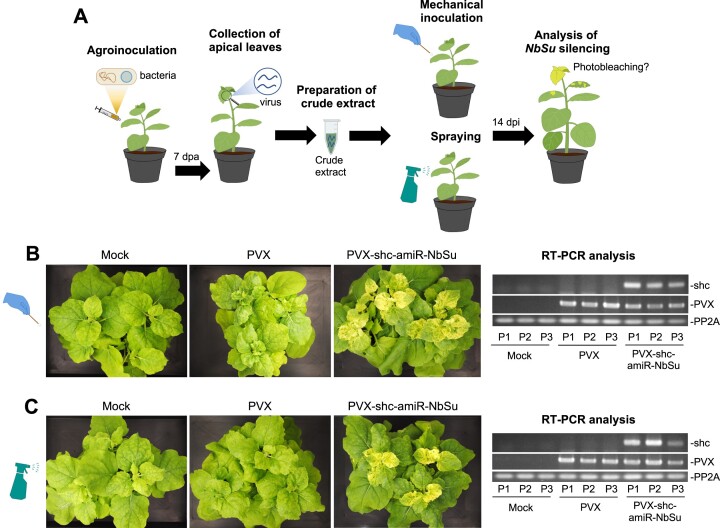
Non-transgenic, DNA-free widespread gene silencing through amiR-VIGS. (**A**) Experimental set up to test gene silencing triggered by *PVX-shc-amiR-NbSu*. (**B**) Widespread *NbSu* silencing induced by mechanically inoculated crude extracts. Left, photos at 14 days post-agroinfiltration (dpa) of sets of three plants mechanically inoculated with different crude extracts obtained from agroinoculated plants. Right, RT-PCR detection of PVX and *shc* precursors in apical leaves at 14 dpa. RT-PCR products corresponding to the control *NbPP2A* are also shown (bottom). (**C**) Widespread *NbSu* silencing induced by sprayed crude extracts. Left, photos at 14 days post-agroinfiltration (dpa) of sets of three plants sprayed with different crude extracts obtained from agroinoculated plants. Right, RT-PCR detection of PVX and *shc* precursors in apical leaves at 14 dpa. Other details are as in (B).

Next, we explored the possibility of triggering widespread silencing in a DNA-free, non-transgenic manner, by using alternative methods to agroinfiltration for inoculating *shc*-containing PVX sequences. For this purpose, a crude extract from bleached apical leaves of each of the three individual plants agroinoculated with *35S:PVX-shc-amiR-NbSu* was prepared. Each crude extract was used as inoculum to mechanically inoculate one young *N. benthamiana* plant (Figure 8A). As controls, similar crude extracts were prepared from mock and empty PVX-agroinoculated plants and analyzed in parallel. Interestingly, all three plants mechanically inoculated with extracts derived from *35S:PVX-shc-amiR-NbSu* agroinfiltrated plants displayed prominent bleaching and accumulated authentic PVX-derived *shc-amiR-NbSu* precursors (Figure 8B) with no sequence alterations as confirmed by sequencing of RT-PCR products. Plants inoculated with extracts from *35S:PVX*-agroinfiltrated plants displayed mild PVX characteristic symptoms and accumulate PVX RNAs, while plants inoculated with extracts derived from mock-inoculated plants remained symptomless and virus-free (Figure 8B).

Finally, we wondered if widespread silencing could also be triggered by applying PVX-shc-amiR-NbSu-containing raw extracts through spraying (Figure 8A). Remarkably, all three plants sprayed with crude extracts derived from *35S:PVX-shc-amiR-NbSu* agroinoculated plants displayed bleached apical leaves which accumulated authentic *shc-amiR-NbSu* precursors with no sequence alterations as confirmed by sequencing of the RT-PCR products (Figure 8C). Importantly, plants sprayed with crude extracts from *35S:PVX*-agroinoculated plants or mock-inoculated plants displayed only mild PVX-derived symptoms or no symptoms at all, respectively, and no bleaching (Figure 8C). These results indicate that crude extracts can be directly sprayed to leaves to trigger amiR-VIGS in *N. benthamiana* in a non-transgenic, DNA-free manner.

## DISCUSSION

In this work, we have systematically analyzed the minimal structural and sequence requirements for producing effective amiRNAs from the 521-nt long *AtMIR390a* precursor. Our analyses show that highly effective amiRNAs can be produced from the considerably shorter *shc* precursor of only 89 nt, including the BS of *AtMIR390a* and the DSL of *OsMIR390* with a 2-nt deletion. The unique ability of *shc* precursors to stably and efficiently express authentic amiRNAs from an RNA viral vector, such as that derived from PVX, highlights the utility of engineering such class of minimal precursors for transgene-free widespread gene silencing in plants.

### Effects on miRNA accumulation of sequence modifications at the BS of the precursor

Systematic mutational analyses done in several plant pri-miRNAs for analyzing miRNA production have shown that, for base-to-loop processed precursors, sequence modifications at the BS region have more deleterious effects in both foldback processing efficiency and accuracy than mutations in the DSL region, while deletions of nucleotides of the ssRNA segments seem largely neutral (4,6). In several plant *MIRNA* precursors, a single change in the BS ∼15 nt below the miRNA is sufficient to completely abolish their processing (47–49), as observed for instance in *AtMIR390a*, in which a G-to-A point mutation in the base of the foldback resulted in misprocessing of the miR390/miR390* duplex and subsequent reduction in *TAS3*-derived tasiRNA levels (50). All in all, these studies support the idea that base-to-loop precursors require a BS of ∼15 nt for DCL1 recognition and accurate first cleavage (9). Our results generally agree with this, as shortening *AtMIR390a* BS drastically affects both amiRNA accumulation and induced target mRNA silencing. Still, it is worth to note that some degree of amiRNA accumulation and target mRNA silencing was observed when expressing amiRNAs from the *BS-D7* precursor, suggesting that DCL1 activity may not be completely abolished when processing *AtMIR390a*-based amiRNA precursors with a BS shorter than 15 nt. Pertinent to this context, it was reported that an artificially shortened form of *AtMIR166b* precursor with only six nucleotides at the BS was processed *in vivo*, releasing miR166b and causing developmental defects (51). Importantly, it seems that an intact BS of *AtMIR390a* is necessary but sufficient for high amiRNA accumulation to levels comparable to those obtained with the *pri-AtMIR390a* precursor. Similarly, it was previously reported that miR171 was efficiently and accurately released from the *AtMIR171* foldback in *N. benthamiana* (52), thus reinforcing the idea that ssRNA segments seem dispensable for efficient and accurate miRNA precursor processing. In contrast, in the case of loop-to-base precursors, the BS region is probably dispensable for processing, as shown with the efficient processing of an *AtMIR157c*-based precursor lacking the BS region (53). In a more recent genome-wide study, a set of sequence features for the processing of plant pri-miRNA precursors were identified (54). In particular, specific nucleotide pairs at the precursors’ DCL1 cleavage sites were observed, with the identity of the nucleotides located at the mismatched positions of the BS having a big impact on the processing efficiency. Here, we did not analyze the effect of changing the identity of the nucleotides that are mismatched in *AtMIR390a* BS. Hence, we cannot rule out that some of these changes might, for instance, increase amiRNA accumulation, as observed for miR172 when expressed from *pri-AtMIR172a* with intentionally paired nucleotides at the first DCL1 cleavage site (54).

### Effects of sequence modifications at the DSL of the precursor on miRNA accumulation

For base-to-loop processed miRNA precursors, a few reports have shown that their native DSL region can be modified without compromising the processing of the precursor. For instance, eliminating bulges at the DSL does not interfere with miR167 biogenesis from *AtMIR167a* precursors (48), while an *AtMIR166b* precursor with a truncated DSL region of only 22-nt accumulated miR166b to levels similar to those obtained with the wild-type precursor (51). Here, deletions of 6–13 nt in the stem of *AtMIR390a* DSL were tolerated, while further shortening of the stem dramatically decreased or abolished amiRNA biogenesis. Interestingly, the chimeric *pri-AtMIR390a-OsDSL* precursor including *OsMIR390* DSL produced high amounts of amiR-NbSu and amiR-NbCla in *N. benthamiana* agroinfiltrated leaves, as reported before for other amiRNA sequences (43). In this work, we further deleted the distal stem of *OsMIR390* while maintaining the terminal loop and found that a 2-nt deletion was tolerated, while longer deletions had a negative effect of amiRNA accumulation. This result suggests that a minimal distal stem length is required for efficient processing, as proposed before for explaining miR172 misprocessing from an *AtMIR172* precursor lacking the terminal loop (47).

### A DNA-free, RNA virus-based system to induce widespread gene silencing in plants

AmiRNAs expressed from viral vectors have been used before in plants for silencing endogenous genes. In particular, nuclear-replicating viruses of DNA genome such as cabbage leaf curl virus (CaLCuV), tomato yellow leaf curl China virus (TYLCCNV) or tomato mottle virus (ToMoV) successfully expressed authentic amiRNAs in *N. benthamiana* (22–24), while the cytoplasmic-replicating tobacco mosaic virus (TMV) of RNA genome failed to produce effective amiRNAs in this same host (23). Interestingly, there is only one example in plants of the use of cytoplasmic-replicating RNA virus [barley stripe mosaic virus (BSMV)] to express amiRNAs (55), although the accumulation of authentic amiRNAs *in vivo* was not proven. Still, a major drawback of the mentioned nuclear-replicating DNA viruses for use in amiR-VIGS is their limited host-range and, in some cases, tissue tropism, with viral replication restricted to phloem cells.

Here we used PVX, a plus (+) single-stranded RNA virus infecting 62 plant species of 27 families including important crops in the family *Solanaceae* (e.g. potato, tomato, pepper or tobacco) (56,57), to efficiently produce authentic amiRNAs in *N. benthamiana* as shown by northern blot and high-throughput sRNA sequencing. Where the *shc* precursors are accurately processed from PVX RNAs to release authentic 21-nt amiRNAs and which DCL is involved in this process is unknown. What is known is that PVX replicates in the cytoplasm and that DCL4 is the primary plant DCL functioning in antiviral defense against RNA viruses and producing 21-nt sRNAs from diced viral RNAs (58,59). It is also known that DCL4, besides its ability for processing long dsRNA precursors, can access flexibly structured single-stranded RNA substrates such as pre-miRNA-like RNAs, as shown in the biogenesis of several sRNAs derived from the satellite RNA of cucumber mosaic virus (60). Despite DCL4 could be the DCL generating amiR-NbSu from PVX RNAs, we cannot rule out that DCL1 might be transiently translocated to the cytoplasm to process the amiRNA precursor, similarly to what has been described with the redistribution of Drosha during a Sindbis virus infection in an animal system (61,62). Alternatively, DCL1 might also act in the cytoplasm right after its synthesis and before being shuttled to the nucleus. We cannot either rule out that PVX RNA fragments that act as amiRNA precursors or the whole PVX genomic RNA enter transiently into the nucleus allowing DCL1 processing and release of the amiRNA, although this last scenario seems more unlikely. In any case, *35S:PVX-amiR-NbSu*-triggered *NbSu* silencing in *DCL4i* knockdown plants is significantly reduced compared to wild-type and DCL1i knockdown *N. benthamiana* plants (Supplementary Figure S10). These results suggest that DCL4 could be the main DCL responsible for the generation of amiR-NbSu from PVX RNAs in the cytoplasm acting directly on the precursor. In any case, the precision in the processing of the *shc* precursor from viral RNAs is remarkable (Figure 7G and Figure S8), and essentially no 21-nt secondary, phased sRNAs derived from amiRNA targets were detected (Data S3, Figure S11) suggesting a lack of transitivity that and reinforces the specificity of the PVX-based amiR-VIGS approach. Interestingly, our data presented in Figure S8 suggests that amiR-NbSu is processed from PVX (+) strand RNAs and not from RdRP-derived dsRNA replication intermediates. Processing of PVX genomic RNA to produce amiR-NbSu seems unlikely, as this may have a deep impact on virus infectivity. Also, it may be difficult that amiR-NbSu precursor folds properly when embedded within the large genomic RNA. In contrast, based on the design of the PVX-based amiR-VIGS construct, the amiR-NbSu precursor is at the 5′ terminus of the subgenomic RNA, which may allow its proper folding for accurate amiR-NbSu release. For all these reasons, amiR-NbSu may be derived from the subgenomic (+) RNA generated during the infection rather than from PVX genomic RNA.

The use of viral vectors in VIGS approaches typically depends on the agroinfiltration of plant tissues with DNA construct(s) for triggering the viral infection (46). Therefore, although not stably transformed, transgenic plant tissues are produced, what certainly limits the biotechnological applications of this type of strategies. Our system uses crude extracts as inoculum, which are obtained from apical (non-agroinfiltrated) leaves infected with PVX variants carrying the amiRNA precursor. Hence, the use of crude extracts for triggering amiR-VIGS circumvents this limitation, while the delivery of raw extracts to plants through spraying should allow to scale up this methodology for large-surface applications in fields. A key aspect to highlight is the high stability of the *shc* precursor, which is retained in PVX genome even after one passage without sequence alterations, despite the well-known proneness of PVX to lose inserted foreign sequences through *in planta* recombination (63). Noteworthy, in this work, we used a PVX vector that incorporates a deletion of the amino-terminal end of the CP and a heterologous promoter derived from another potexvirus, BaMV, that was previously shown to contribute to insert stability (37). In any case, the small size and high sequence stability of the *shc* precursor in PVX, as well as the high specificity intrinsic of the amiRNA approach, make this PVX-based amiR-VIGS strategy an attractive alternative to classic dsRNA-based VIGS in plants. Indeed, the degree of target silencing induced by PVX constructs expressing amiR-NbSu from the *shc* precursor or a *NbSu* transcript fragment of the same size (89 nt) is similar (Supplementary Figure S12). These results support that PVX-based amiR-VIGS with *shc* precursors is highly effective and may be preferred to classic dsRNA-based VIGS because of its higher specificity, at least when similar size inserts are used. AmiRNAs are computationally designed to exclusively silence the intended target(s) while target gene fragments included in classic VIGS constructs typically generate large populations of sRNAs of unpredicted size and sequence that can accidentally target cellular RNAs and induce undesired off-target effects.

### Biotechnological interest of minimal amiRNA precursors

Several plant monocistronic miRNA precursors have been used to express amiRNAs in plants [reviewed in (19)]. Such precursors vary in structure and length, with an average length significantly higher than that of their corresponding miRNA foldback (Supplementary Figure S13A), as most of them correspond to full-length pri-miRNA precursors. To our knowledge, the *shc-MIR390* precursor is the shortest of all amiRNA precursors tested in plants so far, being considerably shorter than its parental *pri-AtMIR390a* precursor (Supplementary Figure S13B). Minimal amiRNA precursors may have several advantages. First, they should be more stable and retained in the viral genome for longer periods, as discussed before for PVX. This advantage may be of particular interest in amiR-VIGS experiments involving viruses with limited cargo capacity. Second, using shorter amiRNA precursor sequences should decrease the cost of producing amiRNA constructs, which is particularly convenient in high-throughput applications where large amounts of amiRNA constructs are generated for plant transformation (64–66). Here, *shc* precursors are inserted into *BS-AtMIR390a-B/*c-based vectors after annealing two oligos of 58 bases, while cloning of amiRNA inserts in previous *AtMIR390a-B/c*-based vectors required the annealing of 75 bases-long oligonucleotides (16). Importantly, the reduction of the oligonucleotide length from 75 to 58 bases should allow for a more cost-effective oligo synthesis and at the lowest production scale (25 nmole) offered by the main oligo-manufacturing companies. And third, minimal amiRNA precursors may be more efficiently synthesized *in vitro* or in bacteria for topical application to plants, among other possible advantages. In any case, the use of minimal amiRNA precursors in a non-transgenic, DNA-free manner should definitely accelerate the development of next-generation RNAi treatments of crops in line with international regulations.

## Supplementary Material

gkad747_Supplemental_files

## Data Availability

High-throughput sequencing data can be found in the Sequence Read Archive (SRA) database under accession number PRJNA957136. New *BS-AtMIR390a-B/c* vectors are available from Addgene at pMDC32B-BS-AtMIR390a-B/c: #199560 (https://www.addgene.org/199560/), pENTR-BS-AtMIR390a-B/c: #199559 (https://www.addgene.org/199559/) .

## Notes

Present address: Anamarija Primc, Center for Organismal Studies (COS), University of Heidelberg, Heidelberg, Germany.

Present address: Javier Sánchez-Vicente, Madeinplant S.L., Valencia, Spain.

## References

[1] Bologna N.G. , Voinnet O. The diversity, biogenesis, and activities of endogenous silencing small RNAs in Arabidopsis. Annu. Rev. Plant Biol. 2014; 65:473–503. 24579988 10.1146/annurev-arplant-050213-035728

[2] Rogers K. , Chen X. Biogenesis, turnover, and mode of action of plant microRNAs. Plant Cell. 2013; 25:2383–2399. 23881412 10.1105/tpc.113.113159 PMC3753372

[3] Achkar N.P. , Cambiagno D.A., Manavella P.A. miRNA biogenesis: a dynamic pathway. Trends Plant Sci. 2016; 21:1034–1044. 27793495 10.1016/j.tplants.2016.09.003

[4] Bajczyk M. , Jarmolowski A., Jozwiak M., Pacak A., Pietrykowska H., Sierocka I., Swida-Barteczka A., Szewc L., Szweykowska-Kulinska Z. Recent insights into plant miRNA biogenesis: multiple layers of miRNA level regulation. Plants. 2023; 12:342. 36679055 10.3390/plants12020342 PMC9864873

[5] Jodder J. Regulation of pri-MIRNA processing: mechanistic insights into the miRNA homeostasis in plant. Plant Cell Rep. 2021; 40:783–798. 33454802 10.1007/s00299-020-02660-7

[6] Li M. , Yu B. Recent advances in the regulation of plant miRNA biogenesis. RNA Biol. 2021; 18:2087–2096. 33666136 10.1080/15476286.2021.1899491 PMC8632083

[7] Zhang L. , Xiang Y., Chen S., Shi M., Jiang X., He Z., Gao S. Mechanisms of MicroRNA biogenesis and stability control in plants. Front. Plant Sci. 2022; 13:844149. 35350301 10.3389/fpls.2022.844149 PMC8957957

[8] Bologna N.G. , Mateos J.L., Bresso E.G., Palatnik J.F. A loop-to-base processing mechanism underlies the biogenesis of plant microRNAs miR319 and miR159. EMBO J. 2009; 28:3646–3656. 19816405 10.1038/emboj.2009.292 PMC2790483

[9] Cuperus J.T. , Fahlgren N., Carrington J.C. Evolution and functional diversification of MIRNA genes. Plant Cell. 2011; 23:431–442. 21317375 10.1105/tpc.110.082784 PMC3077775

[10] Yu Y. , Jia T., Chen X. The ‘how’ and ‘where’ of plant microRNAs. New Phytol. 2017; 216:1002–1017. 29048752 10.1111/nph.14834 PMC6040672

[11] Addo-Quaye C. , Snyder J.A., Park Y.B., Li Y.F., Sunkar R., Axtell M.J. Sliced microRNA targets and precise loop-first processing of MIR319 hairpins revealed by analysis of the Physcomitrella patens degradome. RNA. 2009; 15:2112–2121. 19850910 10.1261/rna.1774909 PMC2779683

[12] Axtell M.J. Classification and comparison of small RNAs from plants. Annu. Rev. Plant Biol. 2013; 64:137–159. 23330790 10.1146/annurev-arplant-050312-120043

[13] Carbonell A. Dalmay T. Artificial small RNA-based strategies for effective and specific gene silencing in plants. Plant Gene Silencing: Mechanisms and Applications. 2017; CABI Publishing 110–127.

[14] Ossowski S. , Schwab R., Weigel D. Gene silencing in plants using artificial microRNAs and other small RNAs. Plant J. 2008; 53:674–690. 18269576 10.1111/j.1365-313X.2007.03328.x

[15] Tiwari M. , Sharma D., Trivedi P.K. Artificial microRNA mediated gene silencing in plants: progress and perspectives. Plant Mol. Biol. 2014; 86:1–18. 25022825 10.1007/s11103-014-0224-7

[16] Carbonell A. , Takeda A., Fahlgren N., Johnson S.C., Cuperus J.T., Carrington J.C. New generation of artificial MicroRNA and synthetic trans-acting small interfering RNA vectors for efficient gene silencing in Arabidopsis. Plant Physiol. 2014; 165:15–29. 24647477 10.1104/pp.113.234989 PMC4012576

[17] Lunardon A. , Kariuki S.M., Axtell M.J. Expression and processing of polycistronic artificial microRNAs and trans-acting siRNAs from transiently introduced transgenes in Solanum lycopersicum and Nicotiana benthamiana. Plant J. 2021; 106:1087–1104. 33655542 10.1111/tpj.15221

[18] Carbonell A. , Lisón P., Daròs J.-A. Multi-targeting of viral RNAs with synthetic trans-acting small interfering RNAs enhances plant antiviral resistance. Plant J. 2019; 100:720–737. 31350772 10.1111/tpj.14466 PMC6899541

[19] Cisneros A.E. , de la Torre-Montaña A., Martín-García T., Carbonell A. Tang G. , Teotia S., Tang X., Singh D. Artificial small RNAs for functional genomics in plants. RNA-Based Technologies for Functional Genomics in Plants, Concepts and Strategies in Plant Sciences. 2021; Cham Springer International Publishing 1–29.

[20] Vasav A.P. , Meshram B.G., Pable A.A., Barvkar V.T. Artificial microRNA mediated silencing of cyclase and aldo–keto reductase genes reveal their involvement in the plumbagin biosynthetic pathway. J. Plant Res. 2023; 136:47–62. 36227455 10.1007/s10265-022-01415-7

[21] Basso M.F. , Ferreira P.C.G., Kobayashi A.K., Harmon F.G., Nepomuceno A.L., Molinari H.B.C., Grossi-de-Sa M.F. MicroRNAs and new biotechnological tools for its modulation and improving stress tolerance in plants. Plant Biotechnol. J. 2019; 17:1482–1500. 30947398 10.1111/pbi.13116 PMC6662102

[22] Ju Z. , Cao D., Gao C., Zuo J., Zhai B., Li S., Zhu H., Fu D., Luo Y., Zhu B. A viral satellite DNA vector (TYLCCNV) for functional analysis of miRNAs and siRNAs in plants. Plant Physiol. 2017; 173:1940–1952. 28228536 10.1104/pp.16.01489 PMC5373046

[23] Kuo Y.-W. , Falk B.W. Artificial microRNA guide strand selection from duplexes with no mismatches shows a purine-rich preference for virus- and non-virus-based expression vectors in plants. Plant Biotechnol. J. 2022; 20:1069–1084. 35113475 10.1111/pbi.13786 PMC9129084

[24] Tang Y. , Wang F., Zhao J., Xie K., Hong Y., Liu Y. Virus-based microRNA expression for gene functional analysis in plants. Plant Physiol. 2010; 153:632–641. 20388670 10.1104/pp.110.155796 PMC2879806

[25] Cisneros A.E. , de la Torre-Montaña A., Carbonell A. Systemic silencing of an endogenous plant gene by two classes of mobile 21-nucleotide artificial small RNAs. Plant J. 2022; 110:1166–1181. 35277899 10.1111/tpj.15730 PMC9310713

[26] Dadami E. , Boutla A., Vrettos N., Tzortzakaki S., Karakasilioti I., Kalantidis K. DICER-LIKE 4 but not DICER-LIKE 2 may have a positive effect on potato spindle tuber viroid accumulation in Nicotiana benthamiana. Mol. Plant. 2013; 6:232–234. 23100483 10.1093/mp/sss118

[27] Clough S.J. , Bent A.F. Floral dip: a simplified method for agrobacterium-mediated transformation of Arabidopsis thaliana. Plant J. 1998; 16:735–743. 10069079 10.1046/j.1365-313x.1998.00343.x

[28] López-Dolz L. , Spada M., Daròs J.-A., Carbonell A. Fine-tune control of targeted RNAi efficacy by plant artificial small RNAs. Nucleic Acids Res. 2020; 48:6234–6250. 32396204 10.1093/nar/gkaa343 PMC7293048

[29] Schwab R. , Ossowski S., Riester M., Warthmann N., Weigel D. Highly specific gene silencing by artificial microRNAs in Arabidopsis. Plant Cell. 2006; 18:1121–1133. 16531494 10.1105/tpc.105.039834 PMC1456875

[30] Fahlgren N. , Hill S.T., Carrington J.C., Carbonell A. P-SAMS: a web site for plant artificial microRNA and synthetic trans-acting small interfering RNA design. Bioinformatics. 2016; 32:157–158. 26382195 10.1093/bioinformatics/btv534 PMC4681993

[31] Nakasugi K. , Crowhurst R., Bally J., Waterhouse P. Combining transcriptome assemblies from multiple de novo assemblers in the allo-tetraploid plant Nicotiana benthamiana. PLoS One. 2014; 9:e91776. 24614631 10.1371/journal.pone.0091776 PMC3948916

[32] Curtis M.D. , Grossniklaus U. A gateway cloning vector set for high-throughput functional analysis of genes in planta. Plant Physiol. 2003; 133:462–469. 14555774 10.1104/pp.103.027979 PMC523872

[33] Carbonell A. Design and high-throughput generation of artificial small RNA constructs for plants. Methods Mol. Biol. 2019; 1932:247–260. 30701506 10.1007/978-1-4939-9042-9_19

[34] Uranga M. , Aragonés V., Selma S., Vázquez-Vilar M., Orzáez D., Daròs J.-A. Efficient Cas9 multiplex editing using unspaced sgRNA arrays engineering in a Potato virus X vector. Plant J. 2021; 106:555–565. 33484202 10.1111/tpj.15164 PMC8251967

[35] Pasin F. , Bedoya L.C., Bernabé-Orts J.M., Gallo A., Simón-Mateo C., Orzaez D., García J.A. Multiple T-DNA delivery to plants using novel mini binary vectors with compatible replication origins. ACS Synth. Biol. 2017; 6:1962–1968. 28657330 10.1021/acssynbio.6b00354

[36] Schürer H. , Lang K., Schuster J., Mörl M. A universal method to produce in vitro transcripts with homogeneous 3′ ends. Nucleic Acids Res. 2002; 30:e56. 12060694 10.1093/nar/gnf055 PMC117298

[37] Dickmeis C. , Fischer R., Commandeur U. Potato virus X-based expression vectors are stabilized for long-term production of proteins and larger inserts. Biotechnol. J. 2014; 9:1369–1379. 25171768 10.1002/biot.201400347

[38] Aragonés V. , Aliaga F., Pasin F., Daròs J.-A. Simplifying plant gene silencing and genome editing logistics by a one-agrobacterium system for simultaneous delivery of multipartite virus vectors. Biotechnol. J. 2022; 17:2100504. 10.1002/biot.202100504 35332696

[39] Cuperus J.T. , Carbonell A., Fahlgren N., Garcia-Ruiz H., Burke R.T., Takeda A., Sullivan C.M., Gilbert S.D., Montgomery T.A., Carrington J.C. Unique functionality of 22-nt miRNAs in triggering RDR6-dependent siRNA biogenesis from target transcripts in Arabidopsis. Nat. Struct. Mol. Biol. 2010; 17:997–1003. 20562854 10.1038/nsmb.1866 PMC2916640

[40] Llave C. , Xie Z., Kasschau K.D., Carrington J.C. Cleavage of scarecrow-like mRNA targets directed by a class of arabidopsis miRNA. Science. 2002; 297:2053–2056. 12242443 10.1126/science.1076311

[41] Carbonell A. , Lopez C., Daròs J.-A. Fast-forward identification of highly effective artificial small RNAs against different tomato spotted wilt virus isolates. Mol. Plant Microbe. Interact. 2019; 32:142–156. 30070616 10.1094/MPMI-05-18-0117-TA

[42] Carbonell A. , Daròs J.-A. Design, synthesis, and functional analysis of highly specific artificial small RNAs with antiviral activity in plants. Methods Mol. Biol. 2019; 2028:231–246. 31228118 10.1007/978-1-4939-9635-3_13

[43] Carbonell A. , Fahlgren N., Mitchell S., Cox K.L. Jr, Carrington J.C Highly specific gene silencing in a monocot species by artificial microRNAs derived from chimeric miRNA precursors. Plant J. 2015; 82:1061–1075. 25809382 10.1111/tpj.12835 PMC4464980

[44] Hannon G.J. FASTX-Toolkit (RRID:SCR_005534). 2010; http://hannonlab.cshl.edu/fastx_toolkit.

[45] Hamilton A. , Voinnet O., Chappell L., Baulcombe D. Two classes of short interfering RNA in RNA silencing. EMBO J. 2002; 21:4671–4679. 12198169 10.1093/emboj/cdf464 PMC125409

[46] Rössner C. , Lotz D., Becker A. VIGS goes viral: how VIGS transforms our understanding of plant science. Annu. Rev. Plant Biol. 2022; 73:703–728. 35138878 10.1146/annurev-arplant-102820-020542

[47] Mateos J.L. , Bologna N.G., Chorostecki U., Palatnik J.F. Identification of MicroRNA processing determinants by random mutagenesis of Arabidopsis MIR172a precursor. Curr. Biol. 2010; 20:49–54. 20005105 10.1016/j.cub.2009.10.072

[48] Song L. , Axtell M.J., Fedoroff N.V. RNA secondary structural determinants of miRNA precursor processing in Arabidopsis. Curr. Biol. 2010; 20:37–41. 20015653 10.1016/j.cub.2009.10.076

[49] Werner S. , Wollmann H., Schneeberger K., Weigel D. Structure determinants for accurate processing of miR172a in Arabidopsis thaliana. Curr. Biol. 2010; 20:42–48. 20015654 10.1016/j.cub.2009.10.073

[50] Cuperus J.T. , Montgomery T.A., Fahlgren N., Burke R.T., Townsend T., Sullivan C.M., Carrington J.C. Identification of MIR390a precursor processing-defective mutants in Arabidopsis by direct genome sequencing. Proc. Natl. Acad. Sci. U.S.A. 2010; 107:466–471. 20018656 10.1073/pnas.0913203107 PMC2806713

[51] Chorostecki U. , Moro B., Rojas A.M.L., Debernardi J.M., Schapire A.L., Notredame C., Palatnik J.F. Evolutionary footprints reveal insights into plant MicroRNA biogenesis. Plant Cell. 2017; 29:1248–1261. 28550151 10.1105/tpc.17.00272 PMC5502457

[52] Parizotto E.A. , Dunoyer P., Rahm N., Himber C., Voinnet O. In vivo investigation of the transcription, processing, endonucleolytic activity, and functional relevance of the spatial distribution of a plant miRNA. Genes Dev. 2004; 18:2237–2242. 15371337 10.1101/gad.307804 PMC517516

[53] Moro B. , Chorostecki U., Arikit S., Suarez I.P., Höbartner C., Rasia R.M., Meyers B.C., Palatnik J.F. Efficiency and precision of microRNA biogenesis modes in plants. Nucleic Acids Res. 2018; 46:10709–10723. 30289546 10.1093/nar/gky853 PMC6237749

[54] Rojas A.M.L. , Drusin S.I., Chorostecki U., Mateos J.L., Moro B., Bologna N.G., Bresso E.G., Schapire A., Rasia R.M., Moreno D.M. et al. Identification of key sequence features required for microRNA biogenesis in plants. Nat. Commun. 2020; 11:5320. 33087730 10.1038/s41467-020-19129-6 PMC7577975

[55] Jian C. , Han R., Chi Q., Wang S., Ma M., Liu X., Zhao H. Virus-based MicroRNA silencing and overexpressing in common wheat (Triticum aestivum L.). Front. Plant Sci. 2017; 8:500. 28443107 10.3389/fpls.2017.00500 PMC5385339

[56] Adams M.J. , Antoniw J.F., Bar-Joseph M., Brunt A.A., Candresse T., Foster G.D., Martelli G.P., Milne R.G., Fauquet C.M. Virology Division News: the new plant virus family Flexiviridae and assessment of molecular criteria for species demarcation. Arch. Virol. 2004; 149:1045–1060. 15098118 10.1007/s00705-004-0304-0

[57] Loebenstein G. , Gaba V. Loebenstein G. , Lecoq H. Chapter 6 - viruses of potato. Advances in Virus Research, Viruses and Virus Diseases of Vegetables in the Mediterranean Basin. 2012; 84:Academic Press 209–246. 10.1016/B978-0-12-394314-9.00006-3 22682169

[58] Bouche N. , Lauressergues D., Gasciolli V., Vaucheret H. An antagonistic function for Arabidopsis DCL2 in development and a new function for DCL4 in generating viral siRNAs. EMBO J. 2006; 25:3347–3356. 16810317 10.1038/sj.emboj.7601217 PMC1523179

[59] Deleris A. , Gallego-Bartolome J., Bao J., Kasschau K.D., Carrington J.C., Voinnet O. Hierarchical action and inhibition of plant dicer-like proteins in antiviral defense. Science. 2006; 313:68–71. 16741077 10.1126/science.1128214

[60] Du Q.-S. , Duan C.-G., Zhang Z.-H., Fang Y.-Y., Fang R.-X., Xie Q., Guo H.-S. DCL4 Targets cucumber mosaic virus satellite RNA at novel secondary structures. J. Virol. 2007; 81:9142–9151. 17609283 10.1128/JVI.02885-06 PMC1951434

[61] Shapiro J.S. , Varble A., Pham A.M., tenOever B.R. Noncanonical cytoplasmic processing of viral microRNAs. RNA. 2010; 16:2068–2074. 20841420 10.1261/rna.2303610 PMC2957047

[62] Shapiro J.S. , Langlois R.A., Pham A.M., tenOever B.R. Evidence for a cytoplasmic microprocessor of pri-miRNAs. RNA. 2012; 18:1338–1346. 22635403 10.1261/rna.032268.112 PMC3383965

[63] Lacomme C. , Chapman S. Use of Potato Virus X (PVX)–Based vectors for gene expression and virus-induced gene silencing (VIGS). Curr. Protoc. Microbiol. 2008; 8:16I.1.1–16I.1.13. 10.1002/9780471729259.mc16i01s8 18770535

[64] Hauser F. , Chen W., Deinlein U., Chang K., Ossowski S., Fitz J., Hannon G.J., Schroeder J.I. A genomic-scale artificial microRNA library as a tool to investigate the functionally redundant gene space in Arabidopsis. Plant Cell. 2013; 25:2848–2863. 23956262 10.1105/tpc.113.112805 PMC3784584

[65] Jover-Gil S. , Paz-Ares J., Micol J.L., Ponce M.R. Multi-gene silencing in Arabidopsis: a collection of artificial microRNAs targeting groups of paralogs encoding transcription factors. Plant J. 2014; 80:149–160. 25040904 10.1111/tpj.12609

[66] Zhang Y. , Nasser V., Pisanty O., Omary M., Wulff N., Di Donato M., Tal I., Hauser F., Hao P., Roth O. et al. A transportome-scale amiRNA-based screen identifies redundant roles of Arabidopsis ABCB6 and ABCB20 in auxin transport. Nat. Commun. 2018; 9:4204. 30310073 10.1038/s41467-018-06410-y PMC6182007

