# Supplementary material for: Transgene-free, virus-based gene silencing in plants by artificial microRNAs derived from minimal precursors
Source: Nucleic Acids Res. 2023 Sep 15;51(19):10719–36. doi: 10.1093/nar/gkad747 (PMC10602918; doi:10.1093/nar/gkad747)
Supplement: gkad747_Supplemental_files [file gkad747_supplemental_files.zip › Supplementary_combined.pdf]

SUPPLEMENTARY DATA

Supplementary Data are available at NAR Online.

Figure S1. Spraying of crude extracts obtained from virus infected plants.

Figure S2. Functional analysis of artificial microRNAs (amiRNAs) against *N. benthamiana 1-DEOXY-D-XYLULOSE-5-PHOSPHATE SYNTHASE* (*NbDXS*) in agroinfiltrated leaves.

Figure S3. *BS-AtMIR390a-B/c*-based vectors for direct cloning of amiRNAs. Top, diagram of the Gateway-compatible *pENTR-BS-AtMIR390a-B/c* entry vector.

Figure S4. Direct cloning of amiRNAs in vectors containing a modified version of *BS-AtMIR390a* that includes a *ccd*B cassette flanked by two *Bsa*I sites (*B*saI/*c*cdB or 'B/c' vectors).

Figure S5. Mapping of 19-24 nucleotide small RNA reads to *pri* and *shc* precursors expressing amiR-NbSu or NbDXS amiRNAs.

Figure S6. Antiviral effects of constructs expressing amiR-TSWV, an amiRNA against *Tomato spotted wilt virus* (TSWV), from *pri* and *shc* precursors.

Figure S7. Mapping of 19-24 nucleotide small RNA reads to *pri* and *shc* precursors expressing amiR-AtFT or AtCH42 amiRNAs.

Figure S8. Mapping of 19-24 nucleotide small RNA reads to PVX-derived sequences expressing amiR-NbSu.

Figure S9. Sequencing analysis of sRNA reads from *35S:shc-amiR-NbSu* agroinfiltrated leaves and from PVX-sch-amiR-NbSu infected tissues.'

Figure S10. Genetic analysis in wild-type (WT) and in *DCL1i* and *DCL4i* knockdown plants of *NbSu* silencing triggered by a *Potato virus X* (PVX) construct expressing amiR-NbSu from the *shc* precursor.

Figure S11. Phasing analysis of amiRNA target RNA-derived 21 nucleotide small RNAs.

Figure S12. Comparative analysis of *Potato virus X* (PVX) constructs expressing amiR-NbSu from the *shc* precursor and a 89-nt long fragment of the *NbSu* gene.

Figure S13. Analysis of the length of *MIRNA* foldbacks and amiRNA precursors used for gene silencing in plants.

Table S1. Name, sequence and use of DNA oligonucleotides used in this study.

Table S2. Phenotypic penetrance of amiRNAs expressed in *A. thaliana* Col-0 T1 transgenic plants.

Appendix S1. Protocol to design and clone amiRNAs downstream the BS region in *BS-AtMIR390a-Bsa*I/*ccd*B-based ('B/c') vectors.

Appendix S2. Protocol to generate PVX-based amiRNA constructs (*shc* precursor)

Appendix S3. FASTA sequences of amiRNA-producing precursors.

Appendix S4. DNA sequence of *Bsa*I-*ccd*B-based (B/c) vectors used for direct cloning of amiRNAs in *MIR390*-based *shc* precursors.

Data S1. Complete list of optimal results generated by P-SAMS amiRNA Designer for the design of amiRNAs against *NbDXS* with no off-targets in *N. benthamiana*.

Data S2. sRNA reads from amiRNA-expressing tissues.

Data S3. sRNA (+) reads of target RNAs and species-specific tasiRNA-generating controls (*AtTAS1c* in *A. thaliana* and *AtTAS3* in *N. benthamiana*).

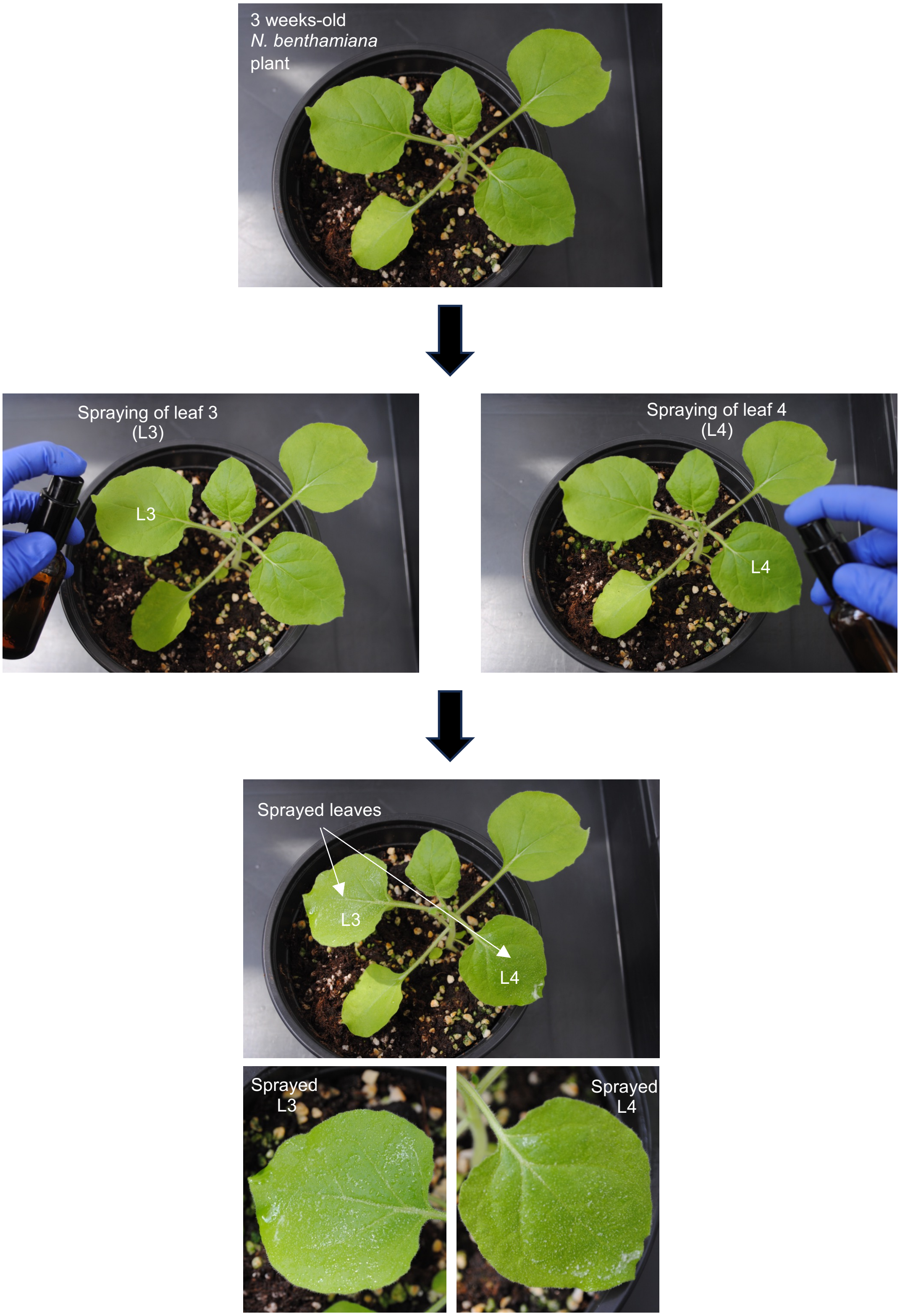

Figure S1. Spraying of crude extracts obtained from virus infected plants. Leaves 3 and 4 (counting form the bottom) of 3 weeks-old *Nicotiana benthamiana* plants (upper photograph) are consecutively sprayed at a 5-10 cm distance (middle photographs) using a high-density polyethylene vaporizer. Bottom photographs show leaves after the spraying.

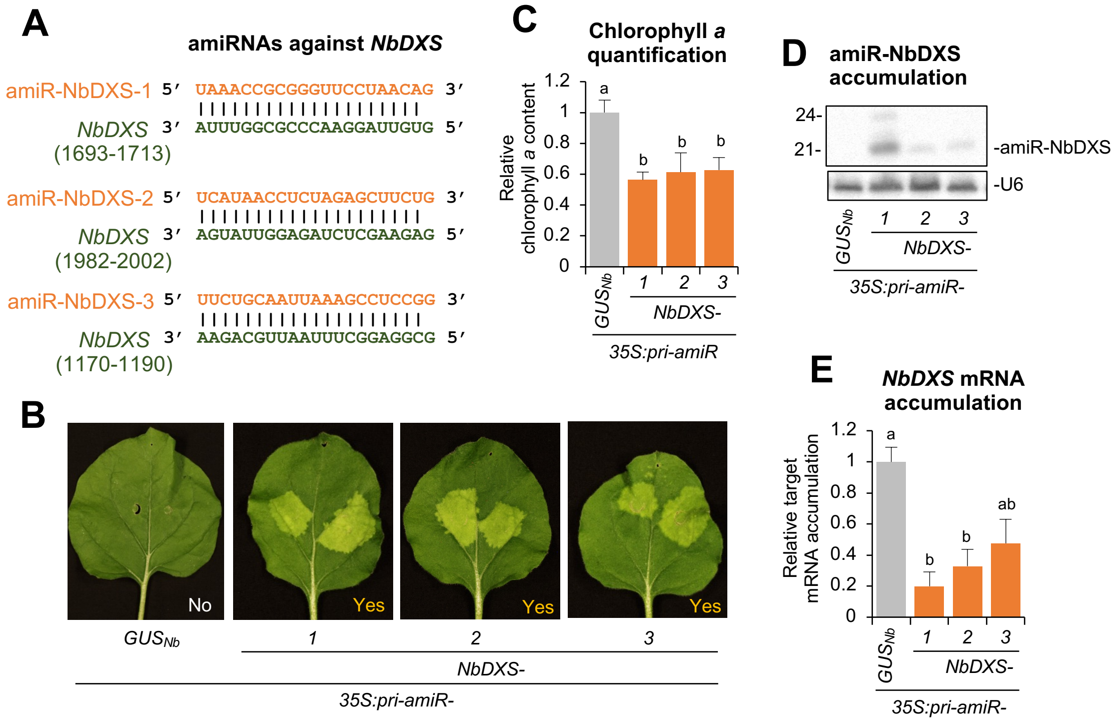

Figure S2. Functional analysis of artificial microRNAs (amiRNAs) against *N. benthamiana 1-DEOXY-D-XYLULOSE-5-PHOSPHATE SYNTHASE* (*NbDXS*) in agroinfiltrated leaves. (a) Base-pairing of amiRNAs and NbDXS target mRNAs. Coordinates of the complete target site in NbDXS mRNAs are given. The arrows indicate the amiRNA-predicted cleavage site. (b) Photographs at 7 days post-agroinfiltration (dpa) of leaves agroinfiltrated with the different amiRNA constructs. Photobleaching appearance or absence is labeled with a "Yes" or a "No". (c) Bar graph showing the relative content of chlorophyll *a* in agroinfiltrated areas (*35S:pri-amiR-GUS*$_{Nb}$ = 1.0). Bars with the letter 'a' are significantly different from that of sample *35S:pri-amiR-GUS*$_{Nb}$ (P < 0.01 in pairwise Student's t-test comparisons). (d) Northern blot detection of amiR-NbDXS amiRNAs in RNA preparations from agroinfiltrated leaves at 2 dpa. (e) Accumulation of *NbDXS* mRNA. Mean mean + SE relative level (n = 3) of *NbDXS* mRNAs after normalization to *PROTEIN PHOSPHATASE 2A* (*PP2A*), as determined by quantitative RT-PCR (qPCR) (*35S:pri-amiR-GUS*$_{Nb}$ = 1.0 in all comparisons). Other details are as shown in (b).

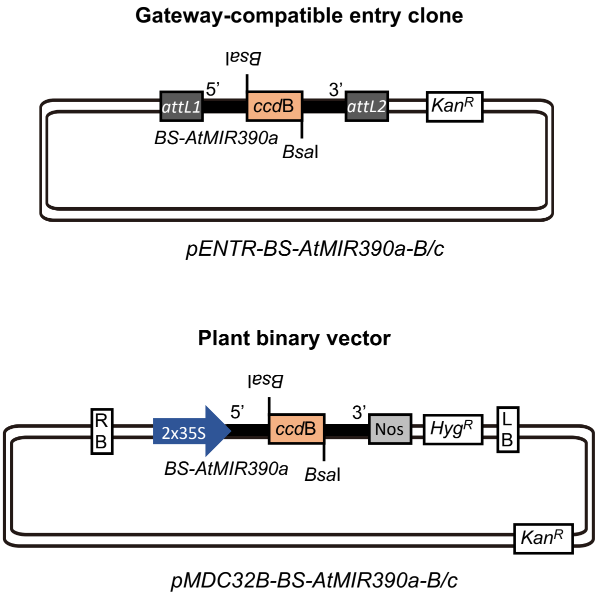

Figure S3. *BS-AtMIR390a-B/c*-based vectors for direct cloning of amiRNAs. Top, diagram of the Gateway-compatible *pENTR-BS-AtMIR390a-B/c* entry vector. Bottom, diagram of the *pMDC32B-BS-AtMIR390a-B/c* binary vector for in plant expression of amiRNAs. RB: right border; 35S: Cauliflower mosaic virus promoter; *Bsa*I: *Bsa*I recognition site, *ccd*B: gene encoding the gyrase toxin; LB: left border; attL1 and attL2: GATEWAY recombination sites. Kan^R: kanamycin resistance gene; Hyg^R: hygromycin resistance gene.

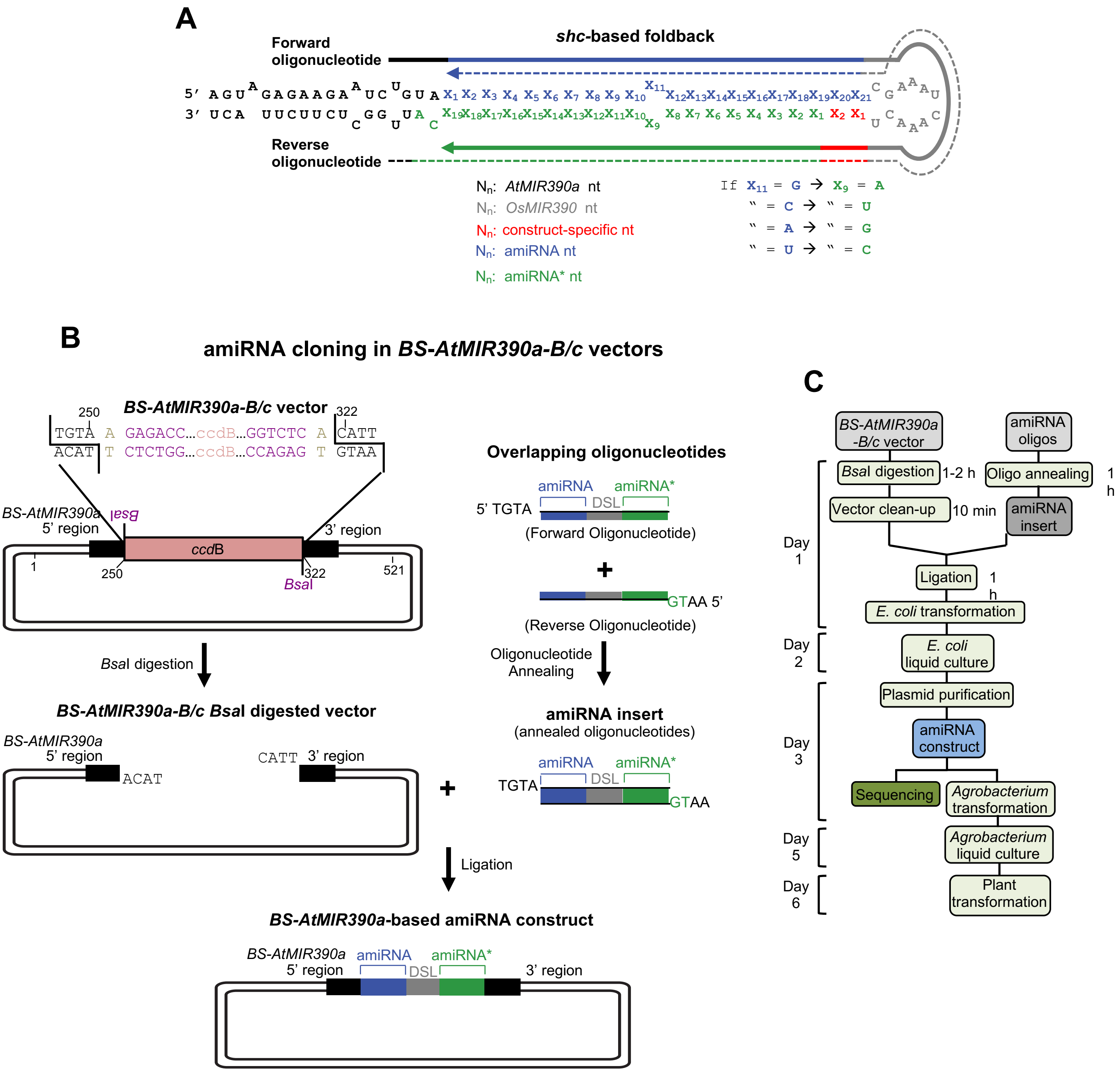

Figure S4. Direct cloning of amiRNAs in vectors containing a modified version of *BS-AtMIR390a* that includes a *ccd*B cassette flanked by two *Bsa*I sites (*Bsa*I/*ccd*B or 'B/c' vectors). A, Design of two overlapping oligonucleotides for amiRNA cloning in *BS-AtMIR390a*-based "B/c" vectors including *OsMIR390* DSL sequences. Sequences covered by the forward and the reverse oligonucleotides are represented with continuous or dotted lines, respectively. Nucleotides of *BS-AtMIR390a* precursor, *OsMIR390*-derived distal stem loop (DSL), amiRNA guide strand and amiRNA* strand are in black, grey, blue and green, respectively. Other nucleotides that may be modified for preserving authentic *OsMIR390a* foldback secondary structure are in red. Rules for assigning identity to position 9 of the amiRNA* are indicated. B, Diagram of the steps for amiRNA cloning in *pre-AtMIR390a-B/c* vectors. The amiRNA insert obtained after annealing the two overlapping oligonucleotides has 5'-TGTA and 5'-AATG overhangs and is directly inserted in a directional manner into a *BS-AtMIR390a-B/c* vector previously linearized with *Bsa*I. Nucleotides of the *Bsa*I sites and those arbitrarily chosen and used as spacers between the *Bsa*I recognition sites and the *BS-AtMIR390a* sequence are in purple and light brown, respectively. Other details are as described in panel A. C, Flowchart of steps from amiRNA construct generation to plant transformation.

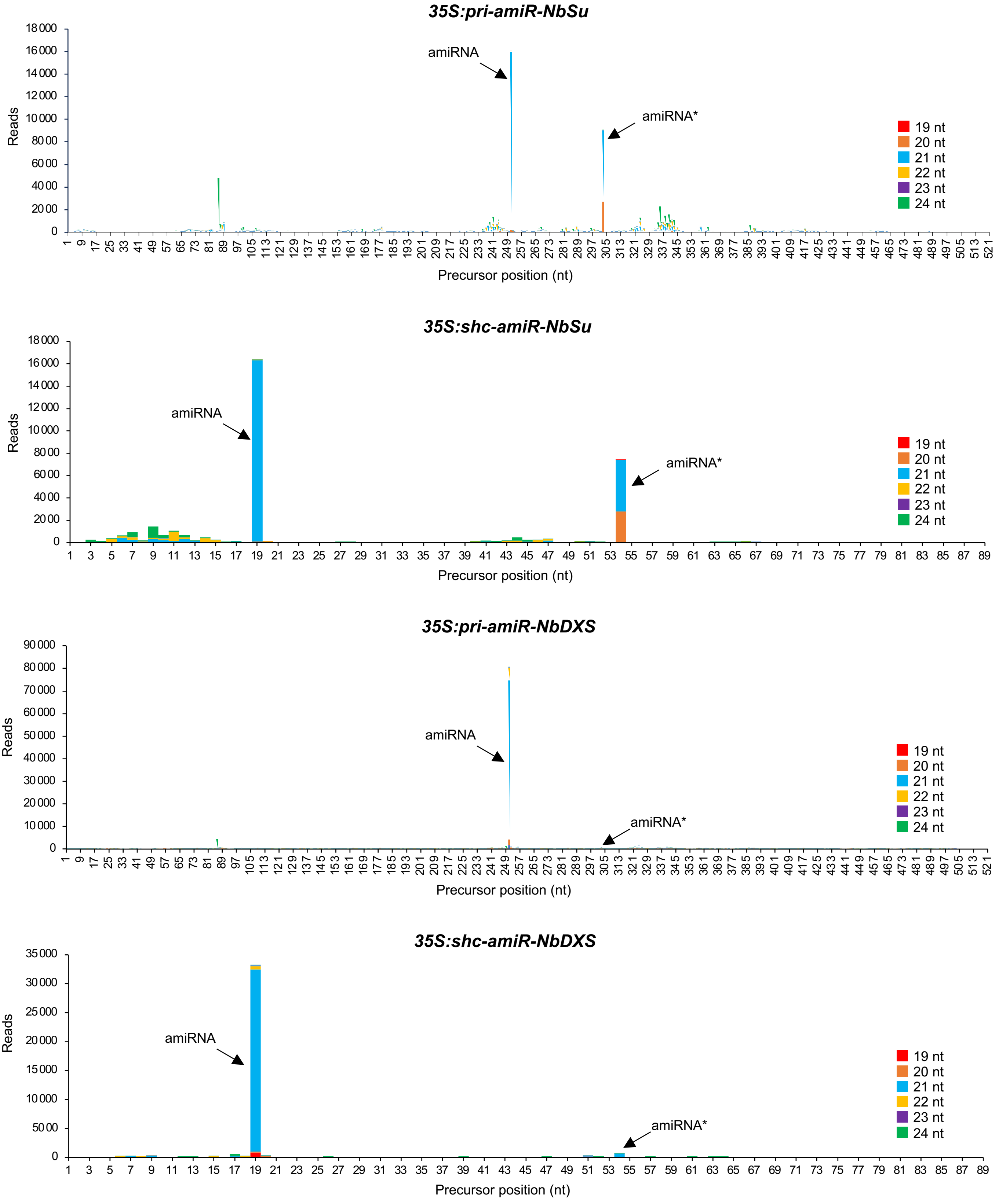

Figure S5. Mapping of 19-24 nucleotide small RNA reads to *pri* and *shc* precursors expressing amiR-NbSu or NbDXS amiRNAs. The *x*-axis indicates the position on the precursor in nucleotides of the 5' end of the sequence plotted. The *y*-axis is the small RNA coverage in total number of reads for each nucleotidic position.

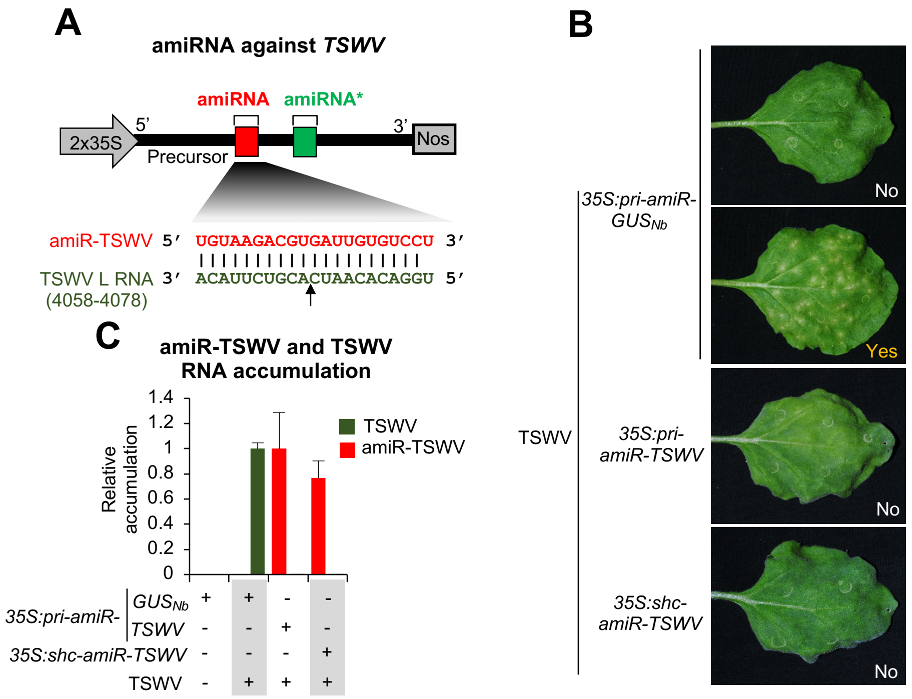

Figure S6. Antiviral effects of constructs expressing amiR-TSWV, an amiRNA against *Tomato spotted wilt virus* (TSWV), from *pri* and *shc* precursors. **A**, Diagram of amiR-TSWV constructs expressing amiR-TSWV directed against TSWV segment L, with amiRNA and star strand positions in the precursor indicated with red and green color, respectively. Base-pairing between amiR-TSWV and its target site is shown, with the predicted cleavage position indicated by an arrow. **B**, Photos at 7 days post-agroinfiltration (dpa) of leaves agroinfiltrated with the different constructs, some of which were further inoculated with TSWV. **C**, Bar graph showing the relative accumulation of amiR-TSWV in agroinfiltrated leaves at 2 dpa [mean relative level (n = 3) + standard deviation amiRNA relative accumulation, *pri-amiR-TSWV* + TSWV= 1.0] and of TSWV RNA in apical leaves at 21 dpa [mean relative level (n = 3) + standard error of TSWV RNAs after normalization to *PROTEIN PHOSPHATASE 2A* (*PP2A*), as determined by quantitative RT-qPCR, *pri-amiR-GUS*$_{Nb}$ + TSWV= 1].

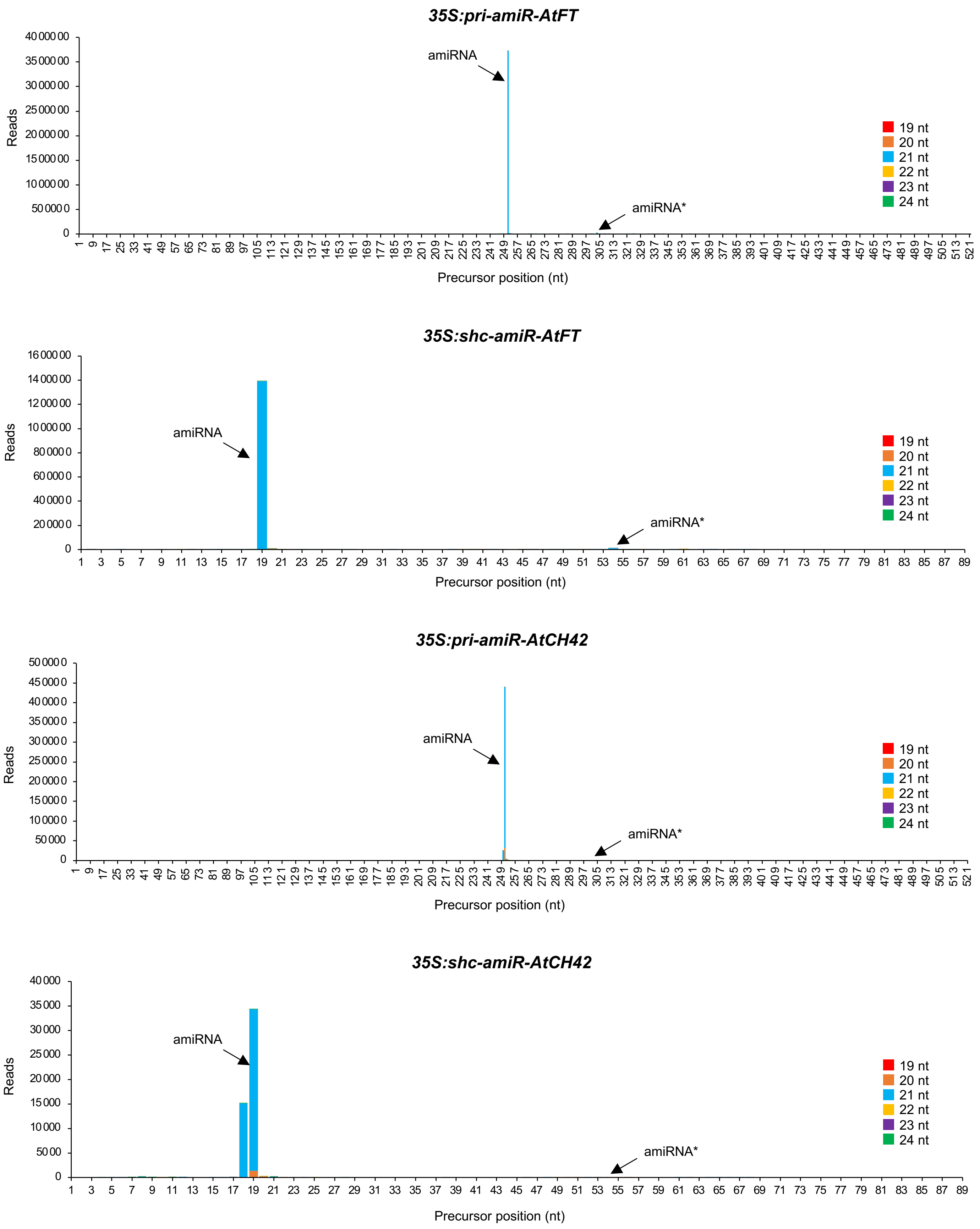

Figure S7. Mapping of 19-24 nucleotide small RNA reads to *pri* and *shc* precursors expressing amiR-AtFT or AtCH42 amiRNAs. The *x*-axis indicates the position on the precursor in nucleotides of the 5' end of the sequence plotted. The *y*-axis is the small RNA coverage in total number of reads for each nucleotidic position.

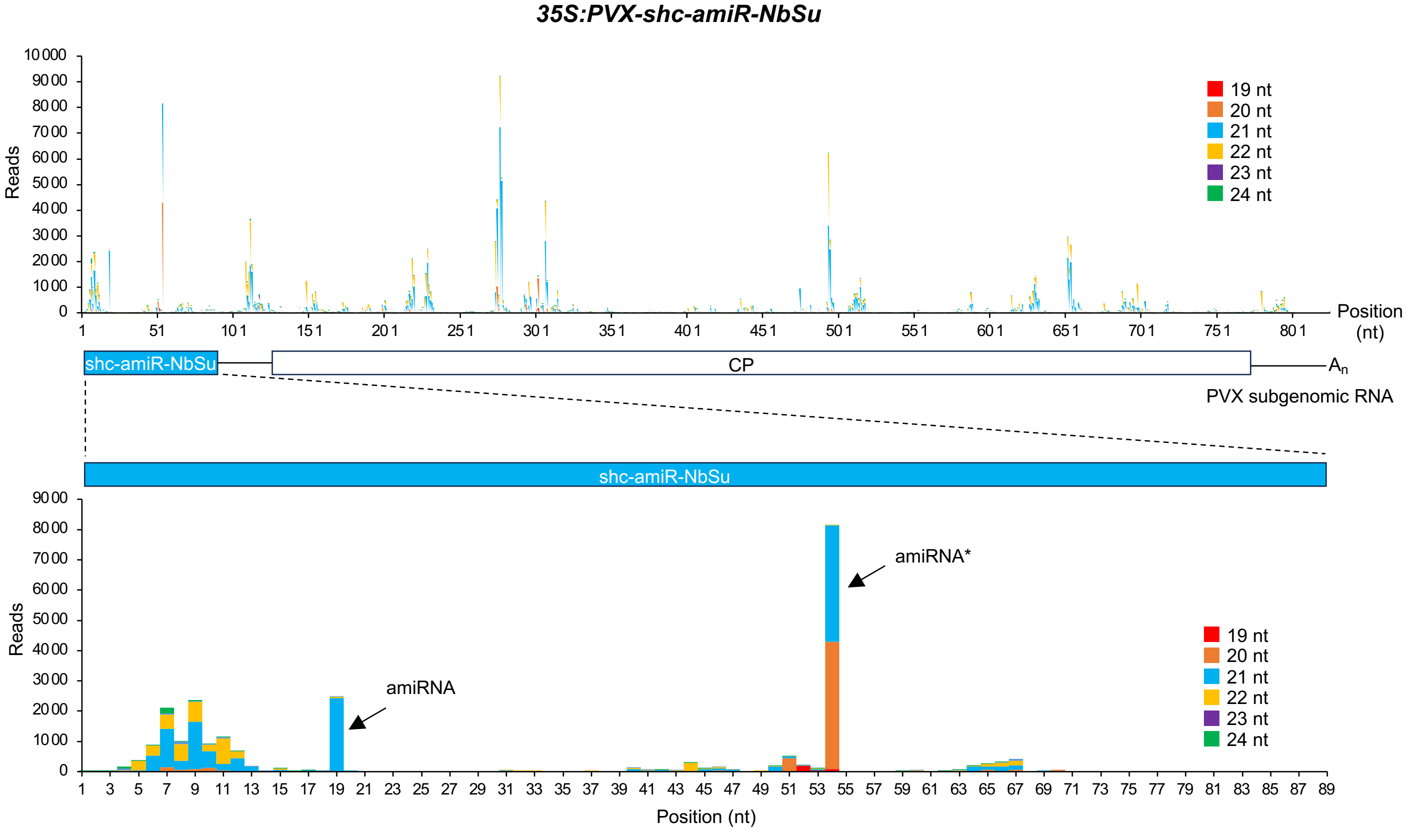

Figure S8. Mapping of 19-24 nucleotide small RNA reads to PVX-derived sequences expressing amiR-NbSu. Top, mapping of reads to the whole subgenomic RNA sequence including PVX coat protein (CP). Bottom, mapping of reads exclusively to the *shc* precursor. The *x*-axis indicates the position on the corresponding RNA sequence (subgenomic RNA or *shc* precursor in top and bottom graphs, respectively) in nucleotides of the 5' end of the sequence plotted. The *y*-axis is the small RNA coverage in total number of reads for each nucleotidic position.

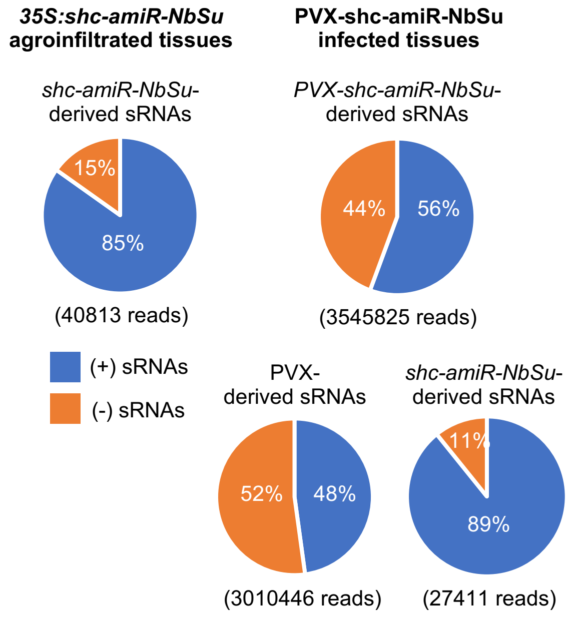

Figure S9. Sequencing analysis of sRNA reads from *35S:shc-amiR-NbSu* agroinfiltrated leaves and from PVX-sch-amiR-NbSu infected tissues. Pie charts showing percentages of reads corresponding to 19-24 nt sRNAs of (+) or (-) polarity (blue and orange sections, respectively).

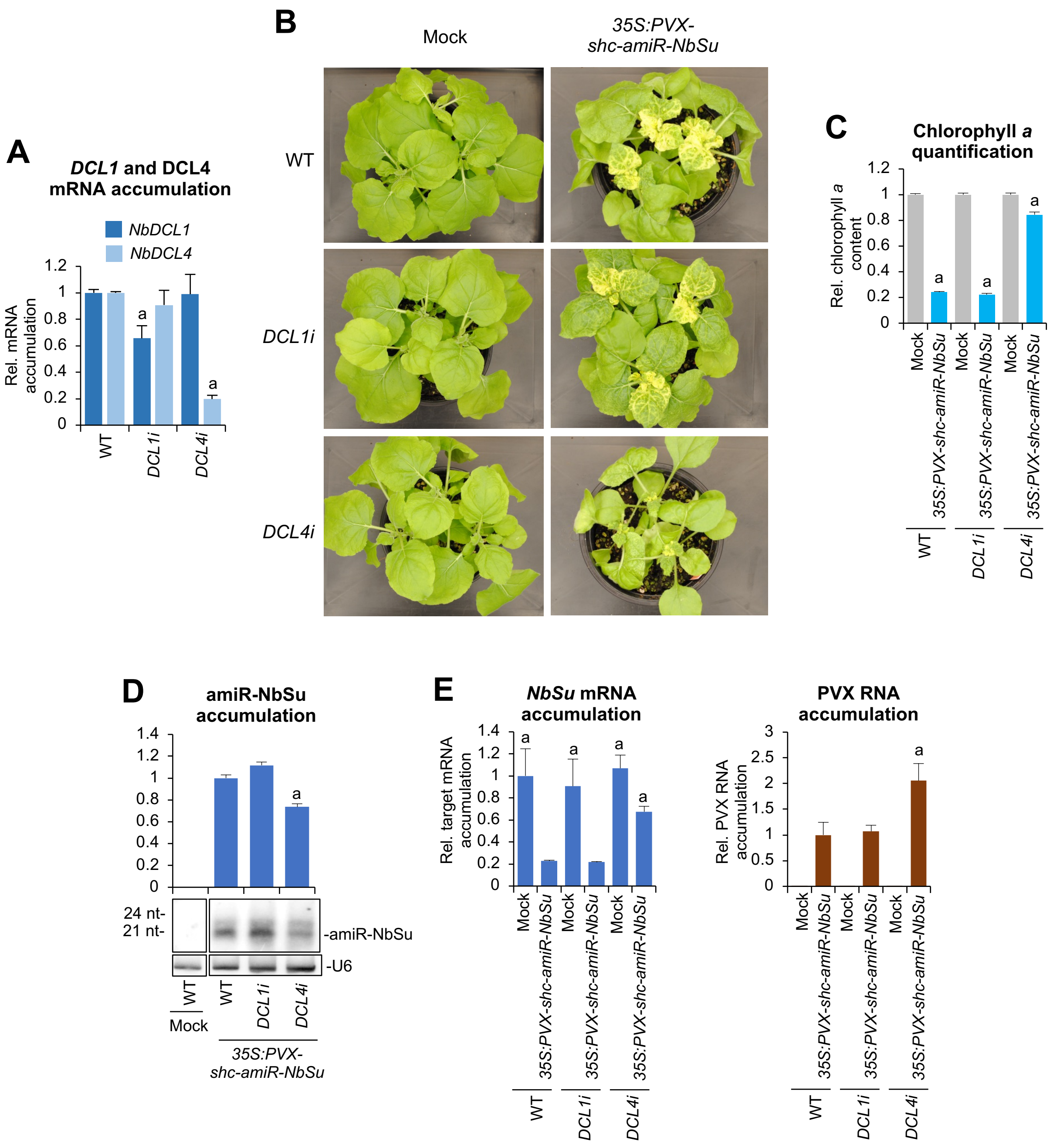

Figure S10. Genetic analysis in wild-type (WT) and in *DCL1i* and *DCL4i* knockdown plants of *NbSu* silencing triggered by a *Potato virus X* (PVX) construct expressing amiR-NbSu from the *shc* precursor. **A**, *NbDCL1* and *NbDCL4* mRNA accumulation in RNA preparations from leaves of WT, *DCL1i* and *DCL4i N. benthamiana* plants. Mean relative level (n = 3) + standard error of mRNAs after normalization to *PROTEIN PHOSPHATASE 2A* (*PP2A*), as determined by RT-qPCR (WT = 1.0 in all comparisons). Bar with the letter "a" is significantly different from that of the corresponding WT samples (P < 0.05 in pairwise Student's t-test comparison). **B**, Photos at 14 days post-agroinfiltration (dpa) of sets of three plants mock inoculated or agroinfiltrated with the *35S:PVX-shc-amiR-NbSu* construct. **C**, Bar graph showing the relative content of chlorophyll *a* in apical leaves from plants mock inoculated or agroinfiltrated with the *35S:PVX-shc-amiR-NbSu* construct (Mock = 1.0). Bar with the letter "a" is significantly different from that of the corresponding Mock control samples (P < 0.05 in pairwise Student's t-test comparison). **D**, Northern blot detection of amiR-NbSu in RNA preparations from apical leaves collected at 14 dpa. The graph at top shows the mean (n = 3) + standard deviation amiRNA relative accumulation (WT = 1.0). Bar with a letter "a" is significantly different from that of the WT sample agroinfiltrated with the *35S:PVX-shc-amiR-NbSu* construct . One blot from three biological replicates is shown. **E**, Target *NbSu* mRNA and PVX RNA accumulation in RNA preparations from apical leaves collected at 7 dpa and analyzed individually. Mean relative level (n = 3) + standard error of *NbSu* mRNAs and PVX RNAs after normalization to *PROTEIN PHOSPHATASE 2A* (*PP2A*), as determined by RT-qPCR (WT + mock = 1.0 in *NbSu* dataset, WT + *35S:PVX-shc-amiR-NbSu* = 1.0 in PVX dataset). Bar with the letter "a" is significantly different from that of the corresponding WT + *35S:PVX-shc-amiR-NbSu* samples (P < 0.05 in pairwise Student's t-test comparison).

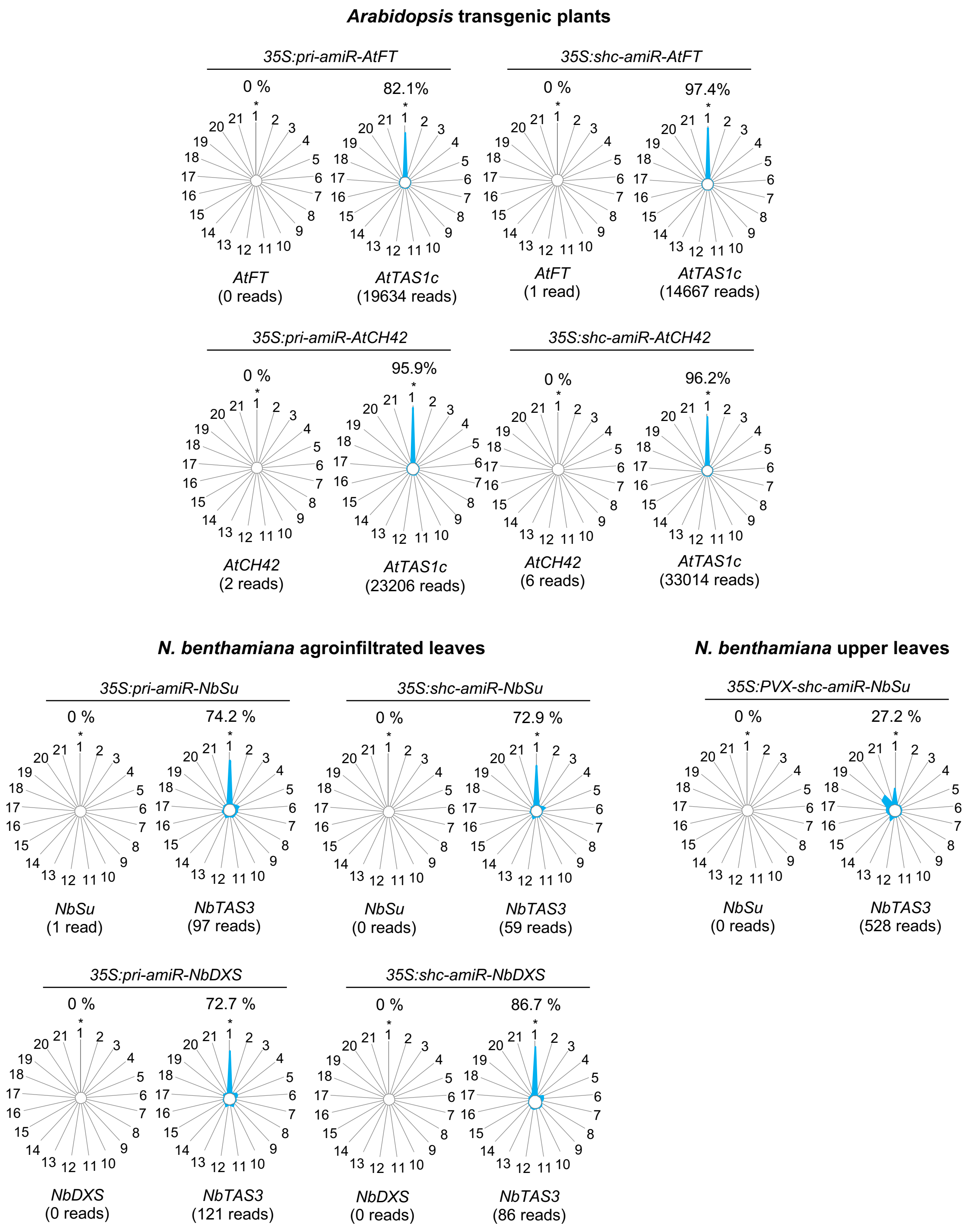

Figure S11. Phasing analysis of amiRNA target RNA-derived 21 nucleotide small RNAs. Radar plots show proportions of 21-nucleotide reads corresponding to each of the 21 registers from *AtFT*, *AtCH42*, *NbSu* and *NbDXS*, with position 1 designated as immediately after the amiRNA guided cleavage site. Control plots for *AtTAS1c* and *NbTAS3* are shown for *A. thaliana* and *N. benthamiana* datasets, respectively. The percentage of 21-nucleotide reads corresponding to phasing register 1 is indicated.

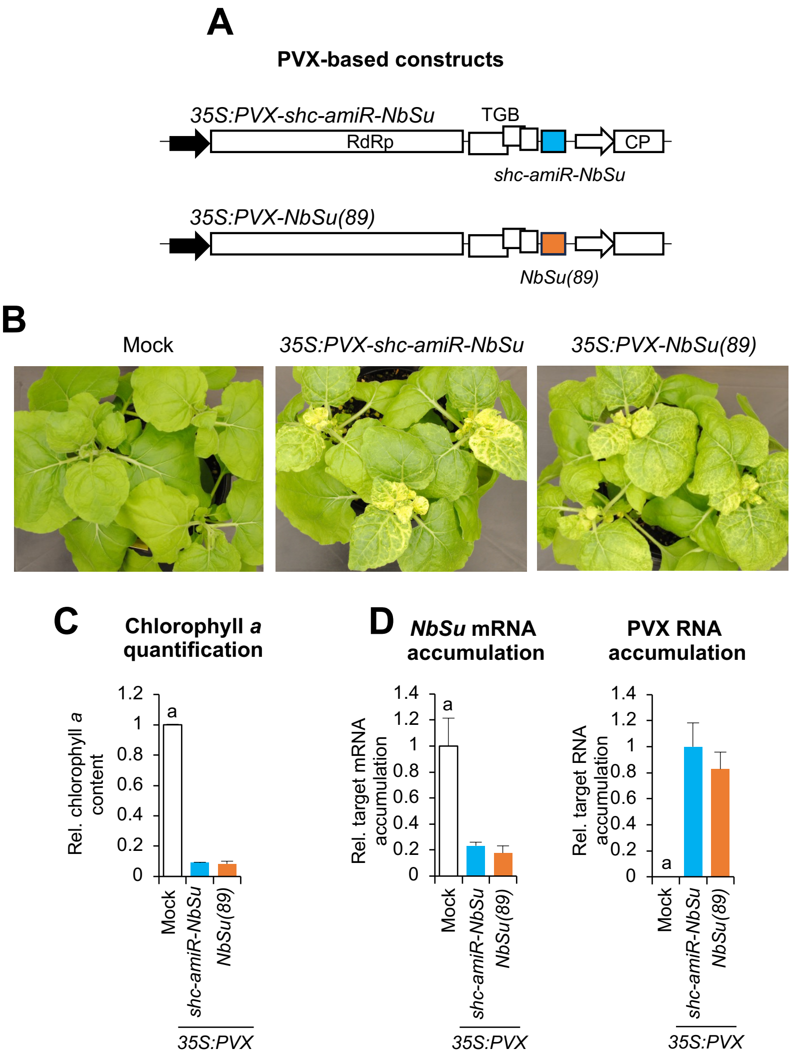

Figure S12. Comparative analysis of *Potato virus X* (PVX) constructs expressing amiR-NbSu from the *shc* precursor and a 89-nt long fragment of the *NbSu* gene. **A**, Diagram of PVX-based constructs. *shc-amiR-NbSu* and *NbSu(89)* cassettes are shown in light blue and orange boxes, respectively. PVX genes RdRp, TGB and CP are represented in white boxes, and CP promoter from *Bamboo mosaic virus* (BaMV) with a white arrow. **B**, Photos at 14 days post-agroinfiltration (dpa) of sets of three plants agroinfiltrated with the different constructs. **C**, Bar graph showing the relative content of chlorophyll *a* in apical leaves from plants agroinfiltrated with different constructs (Mock = 1.0). Bar with the letter "a" is significantly different from that of the corresponding *35S:PVX-shc-amiR-NbSu* samples (P < 0.05 in pairwise Student's t-test comparison). **D**, Target *NbSu* mRNA and PVX RNA accumulation in RNA preparations from apical leaves collected at 7 dpa and analyzed individually. Mean relative level (n = 3) + standard error of *NbSu* mRNAs and PVX RNAs after normalization to *PROTEIN PHOSPHATASE 2A* (*PP2A*), as determined by RT-qPCR (mock = 1.0 in *NbSu* dataset and *35S:PVX-shc-amiR-NbSu* = 1.0 in PVX dataset). Bar with the letter "a" is significantly different from that of the corresponding *35S:PVX-shc-amiR-NbSu* samples (P < 0.05 in pairwise Student's t-test comparison).

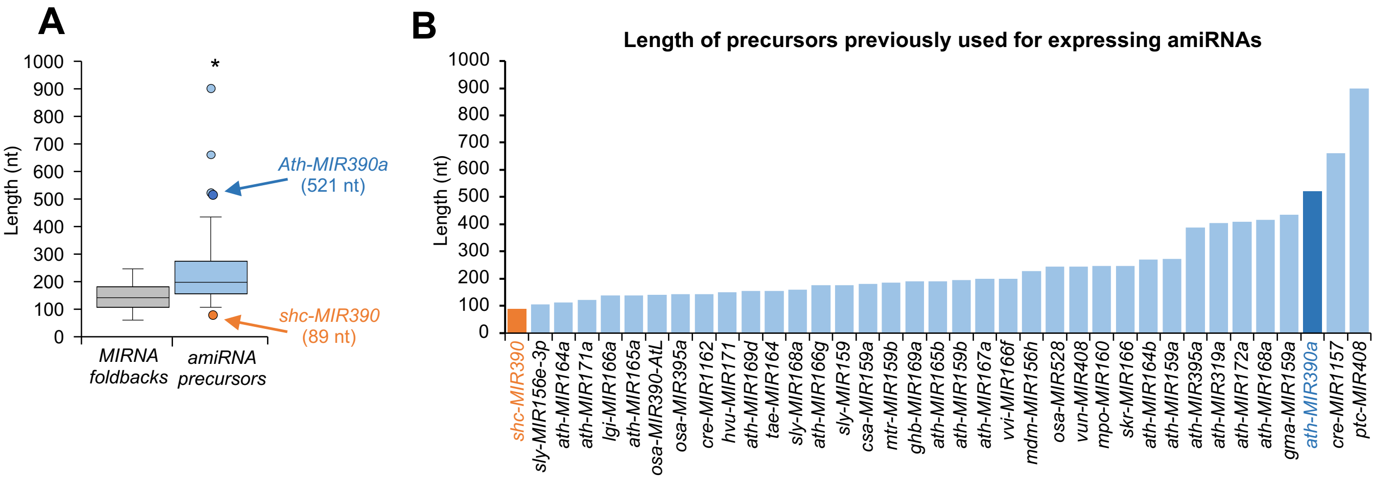

Figure S13. Analysis of the length of *MIRNA* foldbacks and amiRNA precursors used for gene silencing in plants.

Table S1. Name, sequence and use of DNA oligonucleotides used in this study.

Oligonu-cleotide	Sequence	Construct/Aim
AC-55	AGGGGCCATGCTAATCTTCTC	DNA probe for U6 detection
AC-157	GGCCTCTTCCTTTATAACCAA	DNA probe for amiR-AtFT detection
AC-158	AGGGATTTCCGTGACACTTAA	DNA probe for amiR-AtCH42 detection
AC-159	AAAAATGGCTGAGGCTGATGA	qPCR amplification of *AtACT2* mRNA
AC-160	GAAAAACAGCCCTGGGAGC	
AC-163	CATGCACAAGTAGGGACGGTT	qPCR amplification of *AtCH42* mRNA
AC-164	GTCACGGAAATCCTTTGGGTT	
AC-169	TGGAACAACCTTTGGCAATG	qPCR amplification of *AtFT* mRNA
AC-170	CGACACGATGAATTCCTGCA	
AC-251	TGTATAAACCGCGGGTTCCTAACAGATGATGATCACATTCGTT ATCTATTTTTTCTGTTAGGAAACCGCGGTTTA	*35S:pri-amiR-NbDXS-1* (*35S:pri-amiR-NbDXS*)
AC-252	AATGTAAACCGCGGTTTCCTAACAGAAAAAATAGATAACGAAT GTGATCATCATCTGTTAGGAACCCGCGGTTTA	
AC-253	TGTATCATAACCTCTAGAGCTTCTGATGATGATCACATTCGTT ATCTATTTTTTCAGAAGCTCTCGAGGTTATGA	*35S:pri-amiR-NbDXS-2*
AC-254	AATGTCATAACCTCGAGAGCTTCTGAAAAAATAGATAACGAAT GTGATCATCATCAGAAGCTCTAGAGGTTATGA	
AC-255	TGTATTCTGCAATTAAAGCCTCCGGATGATGATCACATTCGTT ATCTATTTTTTCCGGAGGCTTGAATTGCAGAA	*35S:pri-amiR-NbDXS-3*
AC-256	AATGTTCTGCAATTCAAGCCTCCGGAAAAAATAGATAACGAAT GTGATCATCATCCGGAGGCTTTAATTGCAGAA	
AC-270	CTGTTAGGAACCCGCGGTTTA	DNA probe to detect amiR-NbDXS-1
AC-271	CAGAAGCTCTAGAGGTTATGA	DNA probe to detect amiR-NbDXS-2
AC-272	CCGGAGGCTTTAATTGCAGAA	DNA probe to detect amiR-NbDXS-3
AC-335	CACCAGTAGAGAAGAATCTGTA	*pENTR-BS-amiR-NbSu/pMDC32B-BS-amiR-NbSu/ pENTR-BS-amiR-NbDXS/pMDC32B-BS-amiR-NbDXS*
AC-336	AGTAAGAAGAGCCAATGT	
AC-355	GACCCTGATGTTGATGTTCGCT	qPCR amplification of *NbSu* mRNA
AC-356	GAGGGATTTGAAGAGAGATTTC	
AC-359	GGTGGTGGGACTGGTATGAA	qPCR amplification of *NbDXS* mRNA
AC-360	GCAAATCTCACTGGCAGCTT	
AC-365	GACCCTGATGTTGATGTTCGCT	PCR&qPCR amplification of *NbPP2A* mRNA
AC-366	GAGGGATTTGAAGAGAGATTTC	
AC-416	A+GGA+CAC+AAT+CAC+GTC+TTA+CA	LNA probe for amiR-TSWV detection
AC-417	G+CGG+GAA+GTC+CAC+CAC+GGT+TA	LNA probe for amiR-NbSu detection
AC-418	C+TGT+TAG+GAA+CCC+GCG+GTT+TA	LNA probe for amiR-NbDXS detection
AC-484	TGTATAACCGTGGTGGACTTCCCGCTCGAAATCAAACTAGCGG GAAGTCAACCACGGTTA	*35S:OsDSL-amiR-NbSu*
AC-485	AATGTAACCGTGGTTGACTTCCCGCTAGTTTGATTTCGAGCGG GAAGTCCACCACGGTTA	

AC-486	TGTATAACCGTGGTGGACTTCCCGCCGAAATCAAACTGCGGGAAGTCAACCACGGTTA	*35S:OsDSL-Δ2-amiR-NbSu/ 35S:shc-amiR-NbSu*
AC-487	AATGTAACCGTGGTTGACTTCCCGCAGTTTGATTTCGGCGGGAAGTCCACCACGGTTA	
AC-488	TGTATAACCGTGGTGGACTTCCCGCGAAATCAAACGCGGGAAGTCAACCACGGTTA	*35S:OsDSL-Δ4-amiR-NbSu*
AC-489	AATGTAACCGTGGTTGACTTCCCGCGTTTGATTTCGCGGGAAGTCCACCACGGTTA	
AC-490	TGTATAACCGTGGTGGACTTCCCGCAAATCAAAGCGGGAAGTCAACCACGGTTA	*35S:OsDSL-Δ6-amiR-NbSu*
AC-491	AATGTAACCGTGGTTGACTTCCCGCTTTGATTTGCGGGAAGTCCACCACGGTTA	
AC-492	TGTATAACCGTGGTGGACTTCCCGCTCGATTCCTAGCGGGAAGTCAACCACGGTTA	*35S:OsDS-AtL-amiR-NbSu*
AC-493	AATGTAACCGTGGTTGACTTCCCGCTAGGAATCGAGCGGGAAGTCCACCACGGTTA	
AC-494	TGTATAACCGTGGTGGACTTCCCGCGATGATCACATTCGTTATCTATTGCGGGAAGTCAACCACGGTTA	*35S:AtDSL-Δ6-amiR-NbSu*
AC-495	AATGTAACCGTGGTTGACTTCCCGCAATAGATAACGAATGTGATCATCGCGGGAAGTCCACCACGGTTA	
AC-496	TGTATAACCGTGGTGGACTTCCCGCGATCACATTCGTTATCGCGGGAAGTCAACCACGGTTA	*35S:AtDSL-Δ13-amiR-NbSu*
AC-497	AATGTAACCGTGGTTGACTTCCCGCGATAACGAATGTGATCGCGGGAAGTCCACCACGGTTA	
AC-498	TGTATAACCGTGGTGGACTTCCCGCACATTCGTGCGGGAAGTCAACCACGGTTA	*35S:AtDSL-Δ21-amiR-NbSu*
AC-499	AATGTAACCGTGGTTGACTTCCCGCACGAATGTGCGGGAAGTCCACCACGGTTA	
AC-500	TGTATAACCGTGGTGGACTTCCCGCATTCGCGGGAAGTCAACCACGGTTA	*35S:AtDSL-Δ25-amiR-NbSu*
AC-501	AATGTAACCGTGGTTGACTTCCCGCGAATGCGGGAAGTCCACCACGGTTA	
AC-539	GCACTTAACTACAGAGAAATGCAATG	qPCR amplification of *NbDCL4* mRNA
AC-540	ACAATGTTTGAGCGCCTTCT	
AC-558	CACCGAGAAGAATCTGTATAACCGTGGTGGACTTCCCGCATGATGATCACATTCGTTATCTATTTTTTGCGGGAAGTCAACCACGGTTACATTGGCTCTTCTT	*pENTR-BS-Δ7-amiR-NbSu/ 35S:BS-Δ7-amiR-NbSu*
	AAGAAGAGCCAATGTAACCGTGGTTGACTTCCCGCAAAAAATAGATAACGAATGTGATCATCATGCGGGAAGTCCACCACGGTTATACAGATTCTTCTCGGTG	
AC-559	CACCGAATCTGTATAACCGTGGTGGACTTCCCGCATGATGATCACATTCGTTATCTATTTTTTGCGGGAAGTCAACCACGGTTACATTGGCTC	*pENTR-BS-Δ17-amiR-NbSu/ 35S:BS-Δ17-amiR-NbSu*
	GAGCCAATGTAACCGTGGTTGACTTCCCGCAAAAAATAGATAACGAATGTGATCATCATGCGGGAAGTCCACCACGGTTATACAGATTCGGTG	
AC-560	CACCTCTGTATAACCGTGGTGGACTTCCCGCATGATGATCACATTCGTTATCTATTTTTTGCGGGAAGTCAACCACGGTTACATTGG	*pENTR-BS-Δ23-amiR-NbSu/ 35S:BS-Δ23-amiR-NbSu*
	CCAATGTAACCGTGGTTGACTTCCCGCAAAAAATAGATAACGAATGTGATCATCATGCGGGAAGTCCACCACGGTTATACAGAGGTG	
AC-561	CACCTATAACCGTGGTGGACTTCCCGCATGATGATCACATTCGTTATCTATTTTTTGCGGGAAGTCAACCACGGTTACA	*pENTR-BS-Δ31-amiR-NbSu/ 35S:BS-Δ31-amiR-NbSu*
	TGTAACCGTGGTTGACTTCCCGCAAAAAATAGATAACGAATGTGATCATCATGCGGGAAGTCCACCACGGTTATAGGTG	
AC-593	TGTATAAACCGCGGGTTCCTAACAGGATGATCACATTCGTTATCTATTCTGTTAGGAAACCGCGGTTTA	*35S:AtDSL-Δ6-amiR-NbDXS*
AC-594	AATGTAAACCGCGGTTTCCTAACAGAATAGATAACGAATGTGATCATCCTGTTAGGAACCCGCGGTTTA	

AC-595	TGTATAAACCGCGGGTTCCTAACAGGATCACATTCGTTATCCTGTTAGGAAACCGCGGTTTA	*35S:AtDSL-Δ13-amiR-NbDXS*
AC-596	AATGTAAACCGCGGTTTCCTAACAGGATAACGAATGTGATCCTGTTAGGAACCCGCGGTTTA	
AC-597	TGTATAAACCGCGGGTTCCTAACAGACATTCGTCTGTTAGGAAACCGCGGTTTA	*35S:AtDSL-Δ21-amiR-NbDXS*
AC-598	AATGTAAACCGCGGTTTCCTAACAGACGAATGTCTGTTAGGAACCCGCGGTTTA	
AC-599	TGTATAAACCGCGGGTTCCTAACAGATTCCTGTTAGGAAACCGCGGTTTA	*35S:AtDSL-Δ25-amiR-NbDXS*
AC-600	AATGTAAACCGCGGTTTCCTAACAGGAATCTGTTAGGAACCCGCGGTTTA	
AC-601	TGTATAAACCGCGGGTTCCTAACAGTCGAAATCAAACTACTGTTAGGAAACCGCGGTTTA	*35S:OsDSL-amiR-NbDXS*
AC-602	AATGTAAACCGCGGTTTCCTAACAGTAGTTTGATTTCGACTGTTAGGAACCCGCGGTTTA	
AC-603	TGTATAAACCGCGGGTTCCTAACAGCGAAATCAAACTCTGTTAGGAAACCGCGGTTTA	*35S:OsDSL-Δ2-amiR-NbDXS/ 35S:shc-amiR-NbDXS*
AC-604	AATGTAAACCGCGGTTTCCTAACAGAGTTTGATTTCGCTGTTAGGAACCCGCGGTTTA	
AC-605	TGTATAAACCGCGGGTTCCTAACAGGAAATCAAACCTGTTAGGAAACCGCGGTTTA	*35S:OsDSL-Δ4-amiR-NbDXS*
AC-606	AATGTAAACCGCGGTTTCCTAACAGGTTTGATTTCCTGTTAGGAACCCGCGGTTTA	
AC-607	TGTATAAACCGCGGGTTCCTAACAGAAATCAAACTGTTAGGAAACCGCGGTTTA	*35S:OsDSL-Δ6-amiR-NbDXS*
AC-608	AATGTAAACCGCGGTTTCCTAACAGTTTGATTTCTGTTAGGAACCCGCGGTTTA	
AC-609	TGTATAAACCGCGGGTTCCTAACAGTCGATTCCTACTGTTAGGAAACCGCGGTTTA	*35S:OsDS-AtL-amiR-NbDXS*
AC-610	AATGTAAACCGCGGTTTCCTAACAGTAGGAATCGACTGTTAGGAACCCGCGGTTTA	
AC-611	CACCGAGAAGAATCTGTATAAACCGCGGGTTCCTAACAGATGATGATCACATTCGTTATCTATTTTTTCTGTTAGGAAACCGCGGTTTACATTGGCTCTTCTT	*pENTR-BS-Δ7-amiR-NbDXS/ 35S:BS-Δ7-amiR-NbDXS*
	AAGAAGAGCCAATGTAAACCGCGGTTTCCTAACAGAAAAAATAGATAACGAATGTGATCATCATCTGTTAGGAACCCGCGGTTTATACAGATTCTTCTCGGTG	
AC-612	CACCGAATCTGTATAAACCGCGGGTTCCTAACAGATGATGATCACATTCGTTATCTATTTTTTCTGTTAGGAAACCGCGGTTTACATTGGCTC	*pENTR-BS-Δ17-amiR-NbDXS/ 35S:BS-Δ17-amiR-NbDXS*
	GAGCCAATGTAAACCGCGGTTTCCTAACAGAAAAAATAGATAACGAATGTGATCATCATCTGTTAGGAACCCGCGGTTTATACAGATTCGGTG	
AC-613	CACCTCTGTATAAACCGCGGGTTCCTAACAGATGATGATCACATTCGTTATCTATTTTTTCTGTTAGGAAACCGCGGTTTACATTGG	*pENTR-BS-Δ23-amiR-NbDXS/ 35S:BS-Δ23-amiR-NbDXS*
	CCAATGTAAACCGCGGTTTCCTAACAGAAAAAATAGATAACGAATGTGATCATCATCTGTTAGGAACCCGCGGTTTATACAGAGGTG	
AC-614	CACCTATAAACCGCGGGTTCCTAACAGATGATGATCACATTCGTTATCTATTTTTTCTGTTAGGAAACCGCGGTTTACA	*pENTR-BS-Δ31-amiR-NbDXS/ 35S:BS-Δ31-amiR-NbDXS*
	TGTAAACCGCGGTTTCCTAACAGAAAAAATAGATAACGAATGTGATCATCATCTGTTAGGAACCCGCGGTTTATAGGTG	
AC-621	TGTATTGGTTATAAAGGAAGAGGCCCGAAATCAAACTGGCCTCTTCCGTTATAACCAA	*35S:shc-amiR-AtFT*
AC-622	AATGTTGGTTATAACGGAAGAGGCCAGTTTGATTTCGGGCCTCTTCCTTTATAACCAA	
AC-623	TGTATTAAGTGTCACGGAAATCCCTCGAAATCAAACTAGGGATTTCCTTGACACTTAA	*35S:shc-amiR-AtCH42*

AC-624	AATGTTAAGTGTCAAGGAAATCCCTAGTTTGATTTCGAGGGATTTCCGTGACACTTAA	
AC-627	agtaagaagagccaatgTgagaccGGTCTCTTACAGATTCTTCTCTACTGGTG	*pENTR-BS-AtMIR390a-BB*
AC-628	CACCAGTAGAGAAGAATCTGTAAGAGACCggtctcAcattggctcttcttact	
AC-648	gaggtcagcaccagctagcaTATAGGGGGGAAAAAAAGGTAG	*35S:PVX-pri-amiR-*GUS_{Nb}*/ PVX-pri-amiR-NbSu*
AC-650	gaggtcagcaccagctagcaGTAGAGAAGAATCTGTA	*35S:PVX-shc-amiR-NbSu*
AC-654	GGGAATCAATCACAGTGTTGGC	amiRNA precursors detection
AC-655	GCTACTATGGCACGGGCTGTAC	
AC-657	ATGTCAGGCCTGTTCACTATCC	PVX diagnostic
AC-658	TGGTGGTGGTAGAGTGACAAC	
AC-662	gggaaacttaacaaaccctaGAGACTAAAGATGAGATCTAATCTG	*35S:PVX-pri-amiR-*GUS_{Nb}*/ PVX-pri-amiR-NbSu*
AC-663	gggaaacttaacaaaccctaGTAAGAAGAGCCAA	*35S:PVX-shc-amiR-NbSu*
AC-672	TGTATGTAAGACGTGATTGTGTCCTCGAAATCAAACTAGGACACAATAACGTCTTACA	*35S:shc-amiR-TSWV*
AC-673	AATGTGTAAGACGTTATTGTGTCCTAGTTTGATTTCGAGGACACAATCACGTCTTACA	
AC-919	agaggtcagcaccagctagcATTCCTTGGGGTTCTTATCA	*35S:PVX-NbSu(89)*
AC-921	agggaaacttaacaaaccctGCATGCCCAAGTGGGGAC	
AC-923	AAAAGAATGAGATGGTATTTCGG	qPCR amplification of *NbDCL1* mRNA
AC-924	TTCTTTCTGGCATGCTCAA	
AC-927	GAAGTGCTAATGACTGCTAT	qPCR amplification of PVX RNA
AC-928	ACACGGAGGAGCTTACAGAG	
D2065	TGTATAACCGTGGTGGACTTCCCGCATGATGATCACATTCGTTATCTATTTTTTGCGGGAAGTCAACCACGGTTA	*35S:BS-amiR-NbSu*
D2066	AATGTAACCGTGGTTGACTTCCCGCAAAAAATAGATAACGAATGTGATCATCATGCGGGAAGTCCACCACGGTTA	

Table S2: Phenotypic penetrance of amiRNAs expressed in *A. thaliana* Col-0 T1 transgenic plants

Construct	T1 analyzed	Phenotypic penetrance[a]
35S:pri-amiR-GUS$_{Ath}$	48	0%
35S:pri-amiR-AtFT	40	100%
35S:shc-amiR-AtFT	34	100%
35S:pri-amiR-GUS$_{Ath}$	73	0%
35S:pri-amiR-AtCH42	54	100%<br>3.7% weak<br>37% intermediate<br>59.3 % severe
35S:shc-amiR-AtCH42	38	100%<br>2.7% weak<br>34.2% intermediate<br>63.1 % severe

[a] The Ft phenotype was defined as a higher 'days to flowering' value when compared to the average 'days to flowering' value of the *35S:pri-amiR-GUS*$_{Ath}$ control set. Ch42 phenotype is scored in 10 days-old seedling and is considered 'weak', 'intermediate' or 'severe' if seedlings have >2 leaves, exactly 2 leaves or no leaves (only 2 cotyledons), respectively.

Appendix S1

Protocol to design and clone amiRNAs downstream the BS region in *BS-AtMIR390a-Bsa*I/*ccd*B-based ('B/c') vectors.

1. Selection of the amiRNA sequence

Use the amiRNA Designer app from the P-SAMS webtool at http://p-sams.carringtonlab.org/amirna/designer.

2. Design of amiRNA oligonucleotides

Use amiRNA Designer app from the P-SAMS webtool at http://p-sams.carringtonlab.org/amirna/designer.

2.2.1 Sequence of the *BS-AtMIR390a* cassette containing the amiRNA

The following FASTA sequence includes amiRNA/amiRNA* sequences inserted in the *AtMIR390a* precursor sequence downstream the BS region:

>amiRNA in *BS-AtMIR390a*

AGTAGAGAAGAATCTGTA$X_1X_2X_3X_4X_5X_6X_7X_8X_9X_{10}X_{11}X_{12}X_{13}X_{14}X_{15}X_{16}X_{17}X_{18}X_{19}X_{20}X_{21}$CGAAATCAAACT$X_1X_2X_1X_2X_3X_4X_5X_6X_7X_8X_9X_{10}X_{11}X_{12}X_{13}X_{14}X_{15}X_{16}X_{17}X_{18}X_{19}$CA**TTGGCTCTTCTTACT**

Where:

- -X is a DNA base of the amiRNA sequence, and the subscript number is the base position in the amiRNA 21-mer
- -X is a DNA base of the amiRNA* sequence, and the subscript number is the base position in the amiRNA* 21-mer
- -**X** is a DNA base of the BS region of the *AtMIR390a* precursor
- -X is a DNA base of the *OsMIR390* precursor included in the oligonucleotides required to clone the amiRNA insert in B/c vectors

- -**X** is a DNA base of the *AtMIR390a* precursor included in the oligonucleotides required to clone the amiRNA insert in B/c vectors
- -**X** is a DNA base of the *OsMIR390a* precursor that may be modified to preserve the authentic *AtMIR390a* duplex structure

In the sequence above:

-Insert the amiRNA sequence where you see $X_1X_2X_3X_4X_5X_6X_7X_8X_9X_{10}X_{11}X_{12}X_{13}X_{14}X_{15}X_{16}X_{17}X_{18}X_{19}X_{20}X_{21}$

-Insert the amiRNA* sequence that has to verify the following base-pairing:

$$\begin{array}{ccccccccccccccccccccc} X_1 & X_2 & X_3 & X_4 & X_5 & X_6 & X_7 & X_8 & X_9 & X_{10} & X_{11} & X_{12} & X_{13} & X_{14} & X_{15} & X_{16} & X_{17} & X_{18} & X_{19} & X_{20} & X_{21} \\ | & | & | & | & | & | & | & | & | & | & & | & | & | & | & | & | & | & | & | & | \\ X_{19} & X_{18} & X_{17} & X_{16} & X_{15} & X_{14} & X_{13} & X_{12} & X_{11} & X_{10} & X_9 & X_8 & X_7 & X_6 & X_5 & X_4 & X_3 & X_2 & X_1 & X_2 & X_1 \end{array}$$

Note that:

-In general, X_1=T for amiRNA association with AGO1. In this case, X_{19}=A

-Bases X_{11} and X_9 DO NOT base-pair to preserve the central bulge of the authentic *AtMIR390a* duplex. The following base-pair rule applies:

-If X_{11}=G, then X_9=A

-If X_{11}=C, then X_9=T

-If X_{11}=A, then X_9=G

-If X_{11}=U, then X_9=C

2.2.2. Sequence of the amiRNA oligonucleotides

The sequences of the two amiRNA oligonucleotides are:

-Forward oligonucleotide (58 b),

TGTA$X_1X_2X_3X_4X_5X_6X_7X_8X_9X_{10}X_{11}X_{12}X_{13}X_{14}X_{15}X_{16}X_{17}X_{18}X_{19}X_{20}X_{21}$CGAAATCAAACT$X_1X_2X_1X_2X_3X_4X_5X_6X_7X_8X_9X_{10}X_{11}X_{12}X_{13}X_{14}X_{15}X_{16}X_{17}X_{18}X_{19}$

-Reverse oligonucleotide (58 b),

AATG$Y_{19}Y_{18}Y_{17}Y_{16}Y_{15}Y_{14}Y_{13}Y_{12}Y_{11}Y_{10}Y_9Y_8Y_7Y_6Y_5Y_4Y_3Y_2Y_1Y_2Y_1$AGTTTGATTTCG$Y_{21}Y_{20}Y_{19}Y_{18}Y_{17}Y_{16}Y_{15}Y_{14}Y_{13}Y_{12}Y_{11}Y_{10}Y_9Y_8Y_7Y_6Y_5Y_4Y_3Y_2Y_1$

Where:

-$X_1X_2X_3X_4X_5X_6X_7X_8X_9X_{10}X_{11}X_{12}X_{13}X_{14}X_{15}X_{16}X_{17}X_{18}X_{19}X_{20}X_{21}$=amiRNA sequence

-$X_1X_2X_3X_4X_5X_6X_7X_8X_9X_{10}X_{11}X_{12}X_{13}X_{14}X_{15}X_{16}X_{17}X_{18}X_{19}$=partial amiRNA* sequence

-$Y_{21}Y_{20}Y_{19}Y_{18}Y_{17}Y_{16}Y_{15}Y_{14}Y_{13}Y_{12}Y_{11}Y_{10}Y_9Y_8Y_7Y_6Y_5Y_4Y_3Y_2Y_1$=amiRNA reverse-complement sequence

-TG$Y_{19}Y_{18}Y_{17}Y_{16}Y_{15}Y_{14}Y_{13}Y_{12}Y_{11}Y_{10}Y_9Y_8Y_7Y_6Y_5Y_4Y_3Y_2Y_1$=amiRNA* reverse-complement sequence

-X_1X_2= *OsMIR390* sequence that may be modified to preserve authentic *OsMIR390a* duplex structure.

-Y_2Y_1= reverse-complement of X_1X_2

Example:

The sequences of the two oligonucleotides to clone the amiRNA 'amiR-NbSu' (TCCCATTCGATACTGCTCGCC) are:

-Sense oligonucleotide (58 b),

TGTATAACCGTGGTGGACTTCCCGCCGAAATCAAACT**GCGGGAAGTCAACCACGGTTA**

-Antisense oligonucleotide (58 b),

AATGTAACCGTGGTTGACTTCCCGCAGTTTGATTTCG**GCGGGAAGTCCACCACGGTTA**

Note: *the 58 b long oligonucleotides can be ordered desalted, no purification is required.*

3. Cloning of amiRNA sequence(s) in *BS-AtMIR390a-B/c*-based vectors

Notes:

-Available BS-AtMIR390a-B/c vectors are listed in Table I at the end of the section.

-BS-AtMIR390a-B/c-based vectors must be propagated in a ccd*B resistant* E. coli *strain such as DB3.1.*

-Alternatively, Bsa*I digestion of the B/c vector and subsequent ligation of the amiRNA oligonucleotide insert can be done in separate reactions*

3.1. Oligonucleotide annealing

-Dilute sense oligonucleotide and antisense oligonucleotide in sterile H_2O to a final concentration of 100 µM.

-Prepare Oligo Annealing Buffer:

60 mM Tris-HCl (pH 7.5)

500 mM NaCl

60 mM $MgCl_2$

10 mM DTT

Note: *Prepare 1 ml aliquots of Oligo Annealing Buffer and store at -20^{0}C.*

-Assemble the annealing reaction in a PCR tube as described below:

Forward oligonucleotide (100 µM)	2 µL
Reverse oligonucleotide (100 µM)	2 µL

Oligo Annealing Buffer	46 µL
Total volume	50 µL

The final concentration of each oligonucleotide is 4 µM.

-Use a thermocycler to heat the annealing reaction 5 min at 94°C and then cool down (0.05ºC/sec) to 20°C.

-Dilute the annealed oligonucleotides just prior to assembling the digestion-ligation reaction as described below:

Annealed oligonucleotides	3 µL
dH_2O	37 µL
Total volume	40 µL

The final concentration of each oligonucleotide is 0.15 µM.

***Note**: Do not store the diluted oligonucleotides.*

3.2. Digestion-ligation reaction

- Assemble the digestion-ligation reaction as described below:

B/c vector (x ug/uL)	Y µL (50 ng)
Diluted annealed oligonucleotides	1 µL
10x T4 DNA ligase buffer	1 µL
T4 DNA ligase (400 U/µL)	1 µL
*Bsa*I (10U/ µL, NEB)	1 µL
dH_2O	to 10 µL
Total volume	10 µL

Prepare a negative control reaction lacking *Bsa*I.

-Mix the reactions by pipetting. Incubate the reactions at room temperature for 5 minutes at 37°C.

3.3. *E. coli* transformation and analysis of transformants

-Transform 1-5 ul of the digestion-ligation reaction into an *E. coli* strain that doesn't have *ccd*B resistance (e.g. DH10B, TOP10, …) to do counter-selection.

-Pick two colonies/construct, grow LB-Kan (100 mg/ml) cultures and purify plasmids.

-Sequence with appropriate primers: M13-F (CCCAGTCACGACGTTGTAAAACGACGG) and M13-R (CAGAGCTGCCAGGAAACAGCTATGACC) for *pENTR*-based vectors; attB1 (ACAAGTTTGTACAAAAAAGCAGGCT) and attB2 (ACCACTTTGTACAAGAAAGCTGGGT) primers for *pMDC32B*-based vectors).

Table I: *Bsa*I/*ccd*B-based ('B/c') vectors for direct cloning of amiRNAs downstream the BS region in *AtMIR390a* precursor.

Vector	Small RNA expressed	Bacterial antibiotic resistance	Plant antibiotic resistance	GATEWAY use	Backbone	Promoter of syn-tasiRNA cassette	Terminator of syn-tasiRNA cassette	Plant species tested
pENTR-BS-AtMIR390a-B/c	–	Kanamycin	–	Donor	*pENTR*	–	–	–
pMDC32B-BS-AtMIR390a-B/c	amiRNA	Kanamycin Hygromycin	Hygromycin	–	*pMDC32*	*CaMV* 2x35S	*Nos*	*A. thaliana* *N. benthamiana*

Appendix S2

Protocol to generate PVX-based amiRNA constructs (*shc* precursor).

1. Preparation of the dsDNA amiRNA insert

Design and order a dsDNA (129 bp, eg. ultramer duplex in IDT) including the sequences of your amiRNA/amiRNA* inserted into the *shc* (MIR390-based) precursor, as follows:

agaggtcagcaccagctagc**AGTAGAGAAGAATCTGTA**$X_1X_2X_3X_4X_5X_6X_7X_8X_9X_{10}X_{11}X_{12}X_{13}X_{14}X_{15}X_{16}X_{17}X_{18}X_{19}X_{20}X_{21}$CGAAATCAAACT$X_1X_2$$X_1X_2X_3X_4X_5X_6X_7X_8X_9X_{10}X_{11}X_{12}X_{13}X_{14}X_{15}X_{16}X_{17}X_{18}X_{19}$CA**TTGGCTCTTCTTACT**agggtttgttaagtttccct

Where:

-X is a DNA base of the amiRNA sequence, and the subscript number is the base position in the amiRNA 21-mer

-X is a DNA base of the amiRNA* sequence, and the subscript number is the base position in the amiRNA* 21-mer

-**X** is a DNA base of the BS region of the *AtMIR390a* precursor

-X is a DNA base of the *OsMIR390* precursor included in the oligonucleotides required to clone the amiRNA insert in B/c vectors

-X is a DNA base of the *OsMIR390a* precursor that may be modified to preserve the authentic *AtMIR390a* duplex structure

-x is a DNA base of the PVX sequence, required for Gibson-based assembly

In the sequence above:

-Insert the amiRNA sequence where you see

$X_1X_2X_3X_4X_5X_6X_7X_8X_9X_{10}X_{11}X_{12}X_{13}X_{14}X_{15}X_{16}X_{17}X_{18}X_{19}X_{20}X_{21}$

-Insert the amiRNA* sequence that has to verify the following base-pairing:

$X_1\ X_2\ X_3\ X_4\ X_5\ X_6\ X_7\ X_8\ X_9\ X_{10}X_{11}X_{12}X_{13}X_{14}X_{15}X_{16}X_{17}X_{18}\ X_{19}\ X_{20}X_{21}$

| | | | | | | | | | | | | | | | | | | |

$X_{19}X_{18}X_{17}X_{16}X_{15}X_{14}X_{13}X_{12}X_{11}X_{10}X_9\ X_8\ X_7\ X_6\ X_5\ X_4\ X_3\ X_2\ X_1\ X_2\ X_1$

Note that:

-In general, X_1=T for amiRNA association with AGO1. In this case, X_{19}=A

-Bases X_{11} and X_9 DO NOT base-pair to preserve the central bulge of the authentic *AtMIR390a* duplex. The following base-pair rule applies:

-If X_{11}=G, then X_9=A

-If X_{11}=C, then X_9=T

-If X_{11}=A, then X_9=G

-If X_{11}=U, then X_9=C

Fragment #1 (*shc* amiRNA precursor) is ready.

2. Preparation of the vector

-Digest *pLB-PVX-Z* with *Mlu*I.

-Gel purify the 9921 bp band.

-Quantify 1 ul in Nanodrop.

Fragment #2 (backbone vector) is ready.

3. Assembly

-Assemble the Gibbson reaction as described below:

Fragment 1 (dsDNA insert)[a]	
Fragment 2 (vector)[b,c,d]	
GeneArt Gibson Assembly HiFI Master Mix	5 µL
dH_2O	to 10 µL
Total volume	10 µL

[a]The optimal amount of vector is between 50-100 ng

[b]Insert/vector molar excess is between 2-3.

[c]Total DNA amount is between 0.02-0.5 pmol

[d]Mass to moles conversions can be calculated here: http://nebiocalculator.neb.com/#!/ssdnaamt

-Incubate reactions at 50ºC for 1h.

-Clean up reactions with a column (e.g. Zymo Research)

-Transform 1-4 µL in *E. coli* DH5α

-Plate in L-Kan plates and incubate 16h at 37ºC

4. Clone verification

-Pick several colonies and grow in liquid LB-Kan 16h at 37ºC, and purify plasmids.

-Digest candidate clones with *Apa*I+*Xho*I

Good clones: 8595 + **1409** bp

Bad clones (empty *pLB-PVX-Z-MluI*): 8595 + **1738** bp

-Confirm insert sequence by Sanger sequencing with forward and reverse oligos AC-654(GGGAATCAATCACAGTGTTGGC) and/or AC-655 (GCTACTATGGCACGGGCTGTAC), respectively.

Appendix S3.

FASTA sequences of amiRNA-producing precursors.

pri-AtMIR390a
AtMIR390a BS
AtMIR390a DSL
OsMIR390 DSL
amiRNA
amiRNA*

AtCH42

>pri-amiR-AtCH42
TATAGGGGGGAAAAAAAGGTAGTCATCAGATATATATTTTGGTAAGAAAATATAGAAATGAATAATTTCACGTTT
AACGAAGAGGAGATGACGTGTGTTCCTTCGAACCCGAGTTTTGTTCGTCTATAAATAGCACCTTCTCTTCTCCTT
CTTCCTCACTTCCATCTTTTTAGCTTCACTATCTCTCTATAATCGGTTTTATCTTTCTCTAAGTCACAACCCAAA
AAAACAAAGTAGAGAAGAATCTGTATTAAGTGTCACGGAAATCCCTATGATGATCACATTCGTTATCTATTTTTT
AGGGATTTCCTTGACACTTAACATTGGCTCTTCTTACTACAATGAAAAAGGCCGAGGCAAAACGCCTAAAATCAC
TTGAGAATCAATTCTTTTTACTGTCCATTTAAGCTATCTTTTATAAACGTGTCTTATTTTCTATCTCTTTTGTTT
AAACTAAGAAACTATAGTATTTTGTCTAAAACAAAACATGAAAGAACAGATTAGATCTCATCTTTAGTCTC

>shc-amiR-AtCH42
AGTAGAGAAGAATCTGTATTAAGTGTCACGGAAATCCCT**CGAAATCAAACTAG**GGATTTCCTTGACACTTAACAT
TGGCTCTTCTTACT

AtFT

>pri-AtMIR390a-AtFT
TATAGGGGGGAAAAAAAGGTAGTCATCAGATATATATTTTGGTAAGAAAATATAGAAATGAATAATTTCACGTTT
AACGAAGAGGAGATGACGTGTGTTCCTTCGAACCCGAGTTTTGTTCGTCTATAAATAGCACCTTCTCTTCTCCTT
CTTCCTCACTTCCATCTTTTTAGCTTCACTATCTCTCTATAATCGGTTTTATCTTTCTCTAAGTCACAACCCAAA
AAAACAAAGTAGAGAAGAATCTGTATTGGTTATAAAGGAAGAGGCCATGATGATCACATTCGTTATCTATTTTTT
GGCCTCTTCCGTTATAACCAACATTGGCTCTTCTTACTACAATGAAAAAGGCCGAGGCAAAACGCCTAAAATCAC
TTGAGAATCAATTCTTTTTACTGTCCATTTAAGCTATCTTTTATAAACGTGTCTTATTTTCTATCTCTTTTGTTT
AAACTAAGAAACTATAGTATTTTGTCTAAAACAAAACATGAAAGAACAGATTAGATCTCATCTTTAGTCTC

>shc-amiR-AtFT
AGTAGAGAAGAATCTGTATTGGTTATAAAGGAAGAGGCC**CGAAATCAAACTGG**CCTCTTCCGTTATAACCAACAT
TGGCTCTTCTTACT

GUS$_{Nb}$

>pri-amiR-GUS$_{Nb}$
TATAGGGGGGAAAAAAAGGTAGTCATCAGATATATATTTTGGTAAGAAAATATAGAAATGAATAATTTCACGTTT
AACGAAGAGGAGATGACGTGTGTTCCTTCGAACCCGAGTTTTGTTCGTCTATAAATAGCACCTTCTCTTCTCCTT
CTTCCTCACTTCCATCTTTTTAGCTTCACTATCTCTCTATAATCGGTTTTATCTTTCTCTAAGTCACAACCCAAA
AAAACAAAGTAGAGAAGAATCTGTATCTTGTAACGCGCTTTCCCAGATGATGATCACATTCGTTATCTATTTTTT
CTGGGAAAGCTCGTTACAAGACATTGGCTCTTCTTACTACAATGAAAAAGGCCGAGGCAAAACGCCTAAAATCAC
TTGAGAATCAATTCTTTTTACTGTCCATTTAAGCTATCTTTTATAAACGTGTCTTATTTTCTATCTCTTTTGTTT
AAACTAAGAAACTATAGTATTTTGTCTAAAACAAAACATGAAAGAACAGATTAGATCTCATCTTTAGTCTC

>BS-amiR-GUS$_{Nb}$
AGTAGAGAAGAATCTGTATCTTGTAACGCGCTTTCCCAGATGATGATCACATTCGTTATCTATTTTTTCTGGGAA
AGCTCGTTACAAGACATTGGCTCTTCTTACT

NbDXS

>pri-amiR-NbDXS
TATAGGGGGGAAAAAAAGGTAGTCATCAGATATATATTTTGGTAAGAAAATATAGAAATGAATAATTTCACGTTT
AACGAAGAGGAGATGACGTGTGTTCCTTCGAACCCGAGTTTTGTTCGTCTATAAATAGCACCTTCTCTTCTCCTT
CTTCCTCACTTCCATCTTTTTAGCTTCACTATCTCTCTATAATCGGTTTTATCTTTCTCTAAGTCACAACCCAAA
AAAACAAAGTAGAGAAGAATCTGTATAAACCGCGGGTTCCTAACAGATGATGATCACATTCGTTATCTATTTTTT
CTGTTAGGAAACCGCGGTTTACATTGGCTCTTCTTACTACAATGAAAAAGGCCGAGGCAAAACGCCTAAAATCAC
TTGAGAATCAATTCTTTTTACTGTCCATTTAAGCTATCTTTTATAAACGTGTCTTATTTTCTATCTCTTTTGTTT
AAACTAAGAAACTATAGTATTTTGTCTAAAACAAAACATGAAAGAACAGATTAGATCTCATCTTTAGTCTC

>AtDSL-Δ6-amiR-NbDXS
TATAGGGGGGAAAAAAAGGTAGTCATCAGATATATATTTTGGTAAGAAAATATAGAAATGAATAATTTCACGTTT
AACGAAGAGGAGATGACGTGTGTTCCTTCGAACCCGAGTTTTGTTCGTCTATAAATAGCACCTTCTCTTCTCCTT
CTTCCTCACTTCCATCTTTTTAGCTTCACTATCTCTCTATAATCGGTTTTATCTTTCTCTAAGTCACAACCCAAA
AAAACAAAGTAGAGAAGAATCTGTATAAACCGCGGGTTCCTAACAGGATGATCACATTCGTTATCTATTCTGTTA
GGAAACCGCGGTTTACATTGGCTCTTCTTACTACAATGAAAAAGGCCGAGGCAAAACGCCTAAAATCACTTGAGA
ATCAATTCTTTTTACTGTCCATTTAAGCTATCTTTTATAAACGTGTCTTATTTTCTATCTCTTTTGTTTAAACTA
AGAAACTATAGTATTTTGTCTAAAACAAAACATGAAAGAACAGATTAGATCTCATCTTTAGTCTC

> AtDSL-Δ13-amiR-NbDXS
TATAGGGGGGAAAAAAAGGTAGTCATCAGATATATATTTTGGTAAGAAAATATAGAAATGAATAATTTCACGTTT
AACGAAGAGGAGATGACGTGTGTTCCTTCGAACCCGAGTTTTGTTCGTCTATAAATAGCACCTTCTCTTCTCCTT
CTTCCTCACTTCCATCTTTTTAGCTTCACTATCTCTCTATAATCGGTTTTATCTTTCTCTAAGTCACAACCCAAA
AAAACAAAGTAGAGAAGAATCTGTATAAACCGCGGGTTCCTAACAGGATCACATTCGTTATCCTGTTAGGAAACC
GCGGTTTACATTGGCTCTTCTTACTACAATGAAAAAGGCCGAGGCAAAACGCCTAAAATCACTTGAGAATCAATT
CTTTTTACTGTCCATTTAAGCTATCTTTTATAAACGTGTCTTATTTTCTATCTCTTTTGTTTAAACTAAGAAACT
ATAGTATTTTGTCTAAAACAAAACATGAAAGAACAGATTAGATCTCATCTTTAGTCTC

> AtDSL-Δ21-amiR-NbDXS
TATAGGGGGGAAAAAAAGGTAGTCATCAGATATATATTTTGGTAAGAAAATATAGAAATGAATAATTTCACGTTT
AACGAAGAGGAGATGACGTGTGTTCCTTCGAACCCGAGTTTTGTTCGTCTATAAATAGCACCTTCTCTTCTCCTT
CTTCCTCACTTCCATCTTTTTAGCTTCACTATCTCTCTATAATCGGTTTTATCTTTCTCTAAGTCACAACCCAAA
AAAACAAAGTAGAGAAGAATCTGTATAAACCGCGGGTTCCTAACAGACATTCGTCTGTTAGGAAACCGCGGTTTA
CATTGGCTCTTCTTACTACAATGAAAAAGGCCGAGGCAAAACGCCTAAAATCACTTGAGAATCAATTCTTTTTAC
TGTCCATTTAAGCTATCTTTTATAAACGTGTCTTATTTTCTATCTCTTTTGTTTAAACTAAGAAACTATAGTATT
TTGTCTAAAACAAAACATGAAAGAACAGATTAGATCTCATCTTTAGTCTC

> AtDSL-Δ25-amiR-NbDXS
TATAGGGGGGAAAAAAAGGTAGTCATCAGATATATATTTTGGTAAGAAAATATAGAAATGAATAATTTCACGTTT
AACGAAGAGGAGATGACGTGTGTTCCTTCGAACCCGAGTTTTGTTCGTCTATAAATAGCACCTTCTCTTCTCCTT
CTTCCTCACTTCCATCTTTTTAGCTTCACTATCTCTCTATAATCGGTTTTATCTTTCTCTAAGTCACAACCCAAA
AAAACAAAGTAGAGAAGAATCTGTATAAACCGCGGGTTCCTAACAGATTCCTGTTAGGAAACCGCGGTTTACATT
GGCTCTTCTTACTACAATGAAAAAGGCCGAGGCAAAACGCCTAAAATCACTTGAGAATCAATTCTTTTTACTGTC
CATTTAAGCTATCTTTTATAAACGTGTCTTATTTTCTATCTCTTTTGTTTAAACTAAGAAACTATAGTATTTTGT
CTAAAACAAAACATGAAAGAACAGATTAGATCTCATCTTTAGTCTC

>OsDSL-amiR-NbDXS
TATAGGGGGGAAAAAAAGGTAGTCATCAGATATATATTTTGGTAAGAAAATATAGAAATGAATAATTTCACGTTT
AACGAAGAGGAGATGACGTGTGTTCCTTCGAACCCGAGTTTTGTTCGTCTATAAATAGCACCTTCTCTTCTCCTT
CTTCCTCACTTCCATCTTTTTAGCTTCACTATCTCTCTATAATCGGTTTTATCTTTCTCTAAGTCACAACCCAAA
AAAACAAAGTAGAGAAGAATCTGTATAAACCGCGGGTTCCTAACAG**TCGAAATCAACTACT**GTTAGGAAACCGC
GGTTTACATTGGCTCTTCTTACTACAATGAAAAAGGCCGAGGCAAAACGCCTAAAATCACTTGAGAATCAATTCT
TTTTACTGTCCATTTAAGCTATCTTTTATAAACGTGTCTTATTTTCTATCTCTTTTGTTTAAACTAAGAAACTAT
AGTATTTTGTCTAAAACAAAACATGAAAGAACAGATTAGATCTCATCTTTAGTCTC

>OsDSL-Δ2-amiR-NbDXS

TATAGGGGGAAAAAAAGGTAGTCATCAGATATATATTTTGGTAAGAAAATATAGAAATGAATAATTTCACGTTT
AACGAAGAGGAGATGACGTGTGTTCCTTCGAACCCGAGTTTTGTTCGTCTATAAATAGCACCTTCTCTTCTCCTT
CTTCCTCACTTCCATCTTTTTAGCTTCACTATCTCTCTATAATCGGTTTTATCTTTCTCTAAGTCACAACCCAAA
AAAACAAAGTAGAGAAGAATCTGTATAAACCGCGGGTTCCTAACAG**CGAAATCAAACTCT**GTTAGGAAACCGCGG
TTTACATTGGCTCTTCTTACTACAATGAAAAAGGCCGAGGCAAAACGCCTAAAATCACTTGAGAATCAATTCTTT
TTACTGTCCATTTAAGCTATCTTTTATAAACGTGTCTTATTTTCTATCTCTTTTGTTTAAACTAAGAAACTATAG
TATTTTGTCTAAAACAAAACATGAAAGAACAGATTAGATCTCATCTTTAGTCTC

>OsDSL-Δ4-amiR-NbDXS
TATAGGGGGAAAAAAAGGTAGTCATCAGATATATATTTTGGTAAGAAAATATAGAAATGAATAATTTCACGTTT
AACGAAGAGGAGATGACGTGTGTTCCTTCGAACCCGAGTTTTGTTCGTCTATAAATAGCACCTTCTCTTCTCCTT
CTTCCTCACTTCCATCTTTTTAGCTTCACTATCTCTCTATAATCGGTTTTATCTTTCTCTAAGTCACAACCCAAA
AAAACAAAGTAGAGAAGAATCTGTATAAACCGCGGGTTCCTAACAG**GAAATCAAACCT**GTTAGGAAACCGCGGTT
TACATTGGCTCTTCTTACTACAATGAAAAAGGCCGAGGCAAAACGCCTAAAATCACTTGAGAATCAATTCTTTTT
ACTGTCCATTTAAGCTATCTTTTATAAACGTGTCTTATTTTCTATCTCTTTTGTTTAAACTAAGAAACTATAGTA
TTTTGTCTAAAACAAAACATGAAAGAACAGATTAGATCTCATCTTTAGTCTC

>OsDSL-Δ6-amiR-NbDXS
TATAGGGGGAAAAAAAGGTAGTCATCAGATATATATTTTGGTAAGAAAATATAGAAATGAATAATTTCACGTTT
AACGAAGAGGAGATGACGTGTGTTCCTTCGAACCCGAGTTTTGTTCGTCTATAAATAGCACCTTCTCTTCTCCTT
CTTCCTCACTTCCATCTTTTTAGCTTCACTATCTCTCTATAATCGGTTTTATCTTTCTCTAAGTCACAACCCAAA
AAAACAAAGTAGAGAAGAATCTGTATAAACCGCGGGTTCCTAACAG**AAATCAAACT**GTTAGGAAACCGCGGTTTA
CATTGGCTCTTCTTACTACAATGAAAAAGGCCGAGGCAAAACGCCTAAAATCACTTGAGAATCAATTCTTTTTAC
TGTCCATTTAAGCTATCTTTTATAAACGTGTCTTATTTTCTATCTCTTTTGTTTAAACTAAGAAACTATAGTATT
TTGTCTAAAACAAAACATGAAAGAACAGATTAGATCTCATCTTTAGTCTC

>OsDS-AtL-amiR-NbDXS
TATAGGGGGAAAAAAAGGTAGTCATCAGATATATATTTTGGTAAGAAAATATAGAAATGAATAATTTCACGTTT
AACGAAGAGGAGATGACGTGTGTTCCTTCGAACCCGAGTTTTGTTCGTCTATAAATAGCACCTTCTCTTCTCCTT
CTTCCTCACTTCCATCTTTTTAGCTTCACTATCTCTCTATAATCGGTTTTATCTTTCTCTAAGTCACAACCCAAA
AAAACAAAGTAGAGAAGAATCTGTATAAACCGCGGGTTCCTAACAG**TCG**ATTC**CTACT**GTTAGGAAACCGCGGTT
TACATTGGCTCTTCTTACTACAATGAAAAAGGCCGAGGCAAAACGCCTAAAATCACTTGAGAATCAATTCTTTTT
ACTGTCCATTTAAGCTATCTTTTATAAACGTGTCTTATTTTCTATCTCTTTTGTTTAAACTAAGAAACTATAGTA
TTTTGTCTAAAACAAAACATGAAAGAACAGATTAGATCTCATCTTTAGTCTC

>BS-amiR-NbDXS
AGTAGAGAAGAATCTGTATAAACCGCGGGTTCCTAACAGATGATGATCACATTCGTTATCTATTTTTTCTGTTAG
GAAACCGCGGTTTACATTGGCTCTTCTTACT

>BS-Δ7-amiR-NbDXS
GAGAAGAATCTGTATAAACCGCGGGTTCCTAACAGATGATGATCACATTCGTTATCTATTTTTTCTGTTAGGAAA
CCGCGGTTTACATTGGCTCTTCTT

>BS-Δ17-amiR-NbDXS
GAATCTGTATAAACCGCGGGTTCCTAACAGATGATGATCACATTCGTTATCTATTTTTTCTGTTAGGAAACCGCG
GTTTACATTGGCTC

>BS-Δ23-amiR-NbDXS
TCTGTATAAACCGCGGGTTCCTAACAGATGATGATCACATTCGTTATCTATTTTTTCTGTTAGGAAACCGCGGTT
TACATTGG

>BS-Δ31-amiR-NbDXS
TATAAACCGCGGGTTCCTAACAGATGATGATCACATTCGTTATCTATTTTTTCTGTTAGGAAACCGCGGTTTACA

>shc-amiR-NbDXS
AGTAGAGAAGAATCTGTATAAACCGCGGGTTCCTAACAG**CGAAATCAAACTCT**GTTAGGAAACCGCGGTTTACAT
TGGCTCTTCTTACT

NbSu

>pri-amiR-NbSu

TATAGGGGGGAAAAAAAGGTAGTCATCAGATATATATTTTGGTAAGAAAATATAGAAATGAATAATTTCACGTTT
AACGAAGAGGAGATGACGTGTGTTCCTTCGAACCCGAGTTTTGTTCGTCTATAAATAGCACCTTCTCTTCTCCTT
CTTCCTCACTTCCATCTTTTTAGCTTCACTATCTCTCTATAATCGGTTTTATCTTTCTCTAAGTCACAACCCAAA
AAAACAAAGTAGAGAAGAATCTGTATAACCGTGGTGGACTTCCCGCATGATGATCACATTCGTTATCTATTTTTT
GCGGGAAGTCAACCACGGTTACATTGGCTCTTCTTACTACAATGAAAAAGGCCGAGGCAAAACGCCTAAAATCAC
TTGAGAATCAATTCTTTTTACTGTCCATTTAAGCTATCTTTTATAAACGTGTCTTATTTTCTATCTCTTTTGTTT
AAACTAAGAAACTATAGTATTTTGTCTAAAACAAAACATGAAAGAACAGATTAGATCTCATCTTTAGTCTC

>AtDSL-Δ6-amiR-NbSu

TATAGGGGGGAAAAAAAGGTAGTCATCAGATATATATTTTGGTAAGAAAATATAGAAATGAATAATTTCACGTTT
AACGAAGAGGAGATGACGTGTGTTCCTTCGAACCCGAGTTTTGTTCGTCTATAAATAGCACCTTCTCTTCTCCTT
CTTCCTCACTTCCATCTTTTTAGCTTCACTATCTCTCTATAATCGGTTTTATCTTTCTCTAAGTCACAACCCAAA
AAAACAAAGTAGAGAAGAATCTGTATAACCGTGGTGGACTTCCCGCGATGATCACATTCGTTATCTATTGCGGGA
AGTCAACCACGGTTACATTGGCTCTTCTTACTACAATGAAAAAGGCCGAGGCAAAACGCCTAAAATCACTTGAGA
ATCAATTCTTTTTACTGTCCATTTAAGCTATCTTTTATAAACGTGTCTTATTTTCTATCTCTTTTGTTTAAACTA
AGAAACTATAGTATTTTGTCTAAAACAAAACATGAAAGAACAGATTAGATCTCATCTTTAGTCTC

>AtDSL-Δ13-amiR-NbSu

TATAGGGGGGAAAAAAAGGTAGTCATCAGATATATATTTTGGTAAGAAAATATAGAAATGAATAATTTCACGTTT
AACGAAGAGGAGATGACGTGTGTTCCTTCGAACCCGAGTTTTGTTCGTCTATAAATAGCACCTTCTCTTCTCCTT
CTTCCTCACTTCCATCTTTTTAGCTTCACTATCTCTCTATAATCGGTTTTATCTTTCTCTAAGTCACAACCCAAA
AAAACAAAGTAGAGAAGAATCTGTATAACCGTGGTGGACTTCCCGCGATCACATTCGTTATCGCGGGAAGTCAAC
CACGGTTACATTGGCTCTTCTTACTACAATGAAAAAGGCCGAGGCAAAACGCCTAAAATCACTTGAGAATCAATT
CTTTTTACTGTCCATTTAAGCTATCTTTTATAAACGTGTCTTATTTTCTATCTCTTTTGTTTAAACTAAGAAACT
ATAGTATTTTGTCTAAAACAAAACATGAAAGAACAGATTAGATCTCATCTTTAGTCTC

>AtDSL-Δ21-amiR-NbSu

TATAGGGGGGAAAAAAAGGTAGTCATCAGATATATATTTTGGTAAGAAAATATAGAAATGAATAATTTCACGTTT
AACGAAGAGGAGATGACGTGTGTTCCTTCGAACCCGAGTTTTGTTCGTCTATAAATAGCACCTTCTCTTCTCCTT
CTTCCTCACTTCCATCTTTTTAGCTTCACTATCTCTCTATAATCGGTTTTATCTTTCTCTAAGTCACAACCCAAA
AAAACAAAGTAGAGAAGAATCTGTATAACCGTGGTGGACTTCCCGCACATTCGTGCGGGAAGTCAACCACGGTTA
CATTGGCTCTTCTTACTACAATGAAAAAGGCCGAGGCAAAACGCCTAAAATCACTTGAGAATCAATTCTTTTTAC
TGTCCATTTAAGCTATCTTTTATAAACGTGTCTTATTTTCTATCTCTTTTGTTTAAACTAAGAAACTATAGTATT
TTGTCTAAAACAAAACATGAAAGAACAGATTAGATCTCATCTTTAGTCTC

>AtDSL-25-amiR-NbSu

TATAGGGGGGAAAAAAAGGTAGTCATCAGATATATATTTTGGTAAGAAAATATAGAAATGAATAATTTCACGTTT
AACGAAGAGGAGATGACGTGTGTTCCTTCGAACCCGAGTTTTGTTCGTCTATAAATAGCACCTTCTCTTCTCCTT
CTTCCTCACTTCCATCTTTTTAGCTTCACTATCTCTCTATAATCGGTTTTATCTTTCTCTAAGTCACAACCCAAA
AAAACAAAGTAGAGAAGAATCTGTATAACCGTGGTGGACTTCCCGCATTCGCGGGAAGTCAACCACGGTTACATT
GGCTCTTCTTACTACAATGAAAAAGGCCGAGGCAAAACGCCTAAAATCACTTGAGAATCAATTCTTTTTACTGTC
CATTTAAGCTATCTTTTATAAACGTGTCTTATTTTCTATCTCTTTTGTTTAAACTAAGAAACTATAGTATTTTGT
CTAAAACAAAACATGAAAGAACAGATTAGATCTCATCTTTAGTCTC

> OsDSL-amiR-NbSu

TATAGGGGGGAAAAAAAGGTAGTCATCAGATATATATTTTGGTAAGAAAATATAGAAATGAATAATTTCACGTTT
AACGAAGAGGAGATGACGTGTGTTCCTTCGAACCCGAGTTTTGTTCGTCTATAAATAGCACCTTCTCTTCTCCTT
CTTCCTCACTTCCATCTTTTTAGCTTCACTATCTCTCTATAATCGGTTTTATCTTTCTCTAAGTCACAACCCAAA
AAAACAAAGTAGAGAAGAATCTGTATAACCGTGGTGGACTTCCCGC**TCGAAATCAAACTAGC**GGGAAGTCAACCA
CGGTTACATTGGCTCTTCTTACTACAATGAAAAAGGCCGAGGCAAAACGCCTAAAATCACTTGAGAATCAATTCT
TTTTACTGTCCATTTAAGCTATCTTTTATAAACGTGTCTTATTTTCTATCTCTTTTGTTTAAACTAAGAAACTAT
AGTATTTTGTCTAAAACAAAACATGAAAGAACAGATTAGATCTCATCTTTAGTCTC

>OsDSL-Δ2-amiR-NbSu

TATAGGGGGGAAAAAAAGGTAGTCATCAGATATATATTTTGGTAAGAAAATATAGAAATGAATAATTTCACGTTT
AACGAAGAGGAGATGACGTGTGTTCCTTCGAACCCGAGTTTTGTTCGTCTATAAATAGCACCTTCTCTTCTCCTT
CTTCCTCACTTCCATCTTTTTAGCTTCACTATCTCTCTATAATCGGTTTTATCTTTCTCTAAGTCACAACCCAAA
AAAACAAAGTAGAGAAGAATCTGTATAACCGTGGTGGACTTCCCGC**CGAAATCAAACTGC**GGGAAGTCAACCACG
GTTACATTGGCTCTTCTTACTACAATGAAAAAGGCCGAGGCAAAACGCCTAAAATCACTTGAGAATCAATTCTTT
TTACTGTCCATTTAAGCTATCTTTTATAAACGTGTCTTATTTTCTATCTCTTTTGTTTAAACTAAGAAACTATAG
TATTTTGTCTAAAACAAAACATGAAAGAACAGATTAGATCTCATCTTTAGTCTC

>OsDSL-Δ4-amiR-NbSu
TATAGGGGGGAAAAAAAGGTAGTCATCAGATATATATTTTGGTAAGAAAATATAGAAATGAATAATTTCACGTTT
AACGAAGAGGAGATGACGTGTGTTCCTTCGAACCCGAGTTTTGTTCGTCTATAAATAGCACCTTCTCTTCTCCTT
CTTCCTCACTTCCATCTTTTTAGCTTCACTATCTCTCTATAATCGGTTTTATCTTTCTCTAAGTCACAACCCAAA
AAAACAAAGTAGAGAAGAATCTGTATAACCGTGGTGGACTTCCCGC**GAAATCAAACGC**GGGAAGTCAACCACGGT
TACATTGGCTCTTCTTACTACAATGAAAAAGGCCGAGGCAAAACGCCTAAAATCACTTGAGAATCAATTCTTTTT
ACTGTCCATTTAAGCTATCTTTTATAAACGTGTCTTATTTTCTATCTCTTTTGTTTAAACTAAGAAACTATAGTA
TTTTGTCTAAAACAAAACATGAAAGAACAGATTAGATCTCATCTTTAGTCTC

>OsDSL-Δ6-amiR-NbSu
TATAGGGGGGAAAAAAAGGTAGTCATCAGATATATATTTTGGTAAGAAAATATAGAAATGAATAATTTCACGTTT
AACGAAGAGGAGATGACGTGTGTTCCTTCGAACCCGAGTTTTGTTCGTCTATAAATAGCACCTTCTCTTCTCCTT
CTTCCTCACTTCCATCTTTTTAGCTTCACTATCTCTCTATAATCGGTTTTATCTTTCTCTAAGTCACAACCCAAA
AAAACAAAGTAGAGAAGAATCTGTATAACCGTGGTGGACTTCCCGC**AAATCAAAGC**GGGAAGTCAACCACGGTTA
CATTGGCTCTTCTTACTACAATGAAAAAGGCCGAGGCAAAACGCCTAAAATCACTTGAGAATCAATTCTTTTTAC
TGTCCATTTAAGCTATCTTTTATAAACGTGTCTTATTTTCTATCTCTTTTGTTTAAACTAAGAAACTATAGTATT
TTGTCTAAAACAAAACATGAAAGAACAGATTAGATCTCATCTTTAGTCTC

>OsDS-AtL-amiR-NbSu
TATAGGGGGGAAAAAAAGGTAGTCATCAGATATATATTTTGGTAAGAAAATATAGAAATGAATAATTTCACGTTT
AACGAAGAGGAGATGACGTGTGTTCCTTCGAACCCGAGTTTTGTTCGTCTATAAATAGCACCTTCTCTTCTCCTT
CTTCCTCACTTCCATCTTTTTAGCTTCACTATCTCTCTATAATCGGTTTTATCTTTCTCTAAGTCACAACCCAAA
AAAACAAAGTAGAGAAGAATCTGTATAACCGTGGTGGACTTCCCGC**TCG**ATTC**CTAGC**GGGAAGTCAACCACGGT
TACATTGGCTCTTCTTACTACAATGAAAAAGGCCGAGGCAAAACGCCTAAAATCACTTGAGAATCAATTCTTTTT
ACTGTCCATTTAAGCTATCTTTTATAAACGTGTCTTATTTTCTATCTCTTTTGTTTAAACTAAGAAACTATAGTA
TTTTGTCTAAAACAAAACATGAAAGAACAGATTAGATCTCATCTTTAGTCTC

>BS-amiR-NbSu
AGTAGAGAAGAATCTGTATAACCGTGGTGGACTTCCCGCATGATGATCACATTCGTTATCTATTTTTTGCGGGAA
GTCAACCACGGTTACATTGGCTCTTCTTACT

>BS-Δ7-amiR-NbSu
GAGAAGAATCTGTATAACCGTGGTGGACTTCCCGCATGATGATCACATTCGTTATCTATTTTTTGCGGGAAGTCA
ACCACGGTTACATTGGCTCTTCTT

>BS-Δ17-amiR-NbSu
GAATCTGTATAACCGTGGTGGACTTCCCGCATGATGATCACATTCGTTATCTATTTTTTGCGGGAAGTCAACCAC
GGTTACATTGGCTC

>BS-Δ23-amiR-NbSu
TCTGTATAACCGTGGTGGACTTCCCGCATGATGATCACATTCGTTATCTATTTTTTGCGGGAAGTCAACCACGGT
TACATTGG

>BS-Δ31-amiR-NbSu
TATAACCGTGGTGGACTTCCCGCATGATGATCACATTCGTTATCTATTTTTTGCGGGAAGTCAACCACGGTTACA

>shc-amiR-NbSu
AGTAGAGAAGAATCTGTATAACCGTGGTGGACTTCCCGC**CGAAATCAAACTGC**GGGAAGTCAACCACGGTTACAT
TGGCTCTTCTTACT

TSWV

>pri-amiR-TSWV
TATAGGGGGGAAAAAAAGGTAGTCATCAGATATATATTTTGGTAAGAAAATATAGAAATGAATAATTTCACGTTT
AACGAAGAGGAGATGACGTGTGTTCCTTCGAACCCGAGTTTTGTTCGTCTATAAATAGCACCTTCTCTTCTCCTT
CTTCCTCACTTCCATCTTTTTAGCTTCACTATCTCTCTATAATCGGTTTTATCTTTCTCTAAGTCACAACCCAAA
AAAACAAAGTAGAGAAGAATCTGTATGTAAGACGTGATTGTGTCCTATGATGATCACATTCGTTATCTATTTTTT
AGGACACAATAACGTCTTACACATTGGCTCTTCTTACTACAATGAAAAAGGCCGAGGCAAAACGCCTAAAATCAC
TTGAGAATCAATTCTTTTTACTGTCCATTTAAGCTATCTTTTATAAACGTGTCTTATTTTCTATCTCTTTTGTTT
AAACTAAGAAACTATAGTATTTTGTCTAAAACAAAACATGAAAGAACAGATTAGATCTCATCTTTAGTCTC

>shc-amiR-TSWV
AGTAGAGAAGAATCTGTATGTAAGACGTGATTGTGTCCT**CGAAATCAAACTAG**GACACAATAACGTCTTACACAT
TGGCTCTTCTTACT

Appendix S4.

DNA sequence of *Bsa*I-*ccd*B-based (B/c) vectors used for direct cloning of amiRNAs in *MIR390*-based *shc* precursors.

>*pENTR-BS-AtMIR390a-B/c* (4076 bp)
CTTTCCTGCGTTATCCCCTGATTCTGTGGATAACCGTATTACCGCCTTTGAGTGAGCTGATACCGCTCGCCGCAG
CCGAACGACCGAGCGCAGCGAGTCAGTGAGCGAGGAAGCGGAAGAGCGCCCAATACGCAAACCGCCTCTCCCCGC
GCGTTGGCCGATTCATTAATGCAGCTGGCACGACAGGTTTCCCGACTGGAAAGCGGGCAGTGAGCGCAACGCAAT
TAATACGCGTACCGCTAGCCAGGAAGAGTTTGTAGAAACGCAAAAAGGCCATCCGTCAGGATGGCCTTCTGCTTA
GTTTGATGCCTGGCAGTTTATGGCGGGCGTCCTGCCCGCCACCCTCCGGGCCGTTGCTTCACAACGTTCAAATCC
GCTCCCGGCGGATTTGTCCTACTCAGGAGAGCGTTCACCGACAAACAACAGATAAAACGAAAGGCCCAGTCTTCC
GACTGAGCCTTTCGTTTTATTTGATGCCTGGCAGTTCCCTACTCTCGCGTTAACGCTAGCATGGATGTTTTCCCA
GTCACGACGTTGTAAAACGACGGCCAGTCTTAAGCTCGGGCCCCAAATAATGATTTTATTTTGACTGATAGTGAC
CTGTTCGTTGCAACAAATTGATGAGCAATGCTTTTTTATAATGCCAACTTTGTACAAAAAAGCAGGCTCCGCGGC
CGCCCCCTTCACCGTAGAGAAGAATCTGTAAGAGACCATTAGGCACCCCAGGCTTTACACTTTATGCTTCCGGCT
CGTATAATGTGTGGATTTTGAGTTAGGAGCCGTCGAGATTTTCAGGAGCTAAGGAAGCTAAAatggagaaaaaaa
tcactggatataccaccgttgatatatcccaatggcatcgtaaagaacattttgaggcatttcagtcagttgctc
aatgtacctataaccagaccgttcagctggatattacggcctttttaaagaccgtaaagaaaaataagcacaagt
tttatccggcctttattcacattcttgcccgcctgatgaatgctcatccggagttccgtatggcaatgaaagacg
gtgagctggtgatatgggatagtgttcacccttgttacaccgttttccatgagcaaactgaaacgttttcatcgc
tctggagtgaataccacgacgatttccggcagtttctacacatatattcgcaagatgtggcgtgttacggtgaaa
acctggcctatttccctaaagggtttattgagaatatgtttttcgtctcagccaatccctgggtgagtttcacca
gttttgatttaaacgtggccaatatggacaacttcttcgcccccgttttcaccatgggcaaatattatacgcaag
gcgacaaggtgctgatgccgctggcgattcaggttcatcatgccgtttgtgatggcttccatgtcggcagaatgc
ttaatgaattacaacagtactgcgatgagtggcagggcggggcgtaaACGCGTGGAGCCGGCTTACTAAAAGCCA
GATAACAGTATGCGTATTTGCGCGCTGATTTTTGCGGTATAAGAATATATACTGATATGTATACCCGAAGTATGT
CAAAAAGAGGTATGCTATGAAGCAGCGTATTACAGTGACAGTTGACAGCGACAGCTATCAGTTGCTCAAGGCATA
TATGATGTCAATATCTCCGGTCTGGTAAGCACAACCATGCAGAATGAAGCCCGTCGTCTGCGTGCCGAACGCTGG
AAAGCGGAAAATCAGGAAGGGATGGCTGAGGTCGCCCGGTTTATTGAAATGAACGGCTCTTTTGCTGACGAGAAC
AGGGGCTGGTGAAATGCAGTTTAAGGTTTACACCTATAAAAGAGAGAGCCGTTATCGTCTGTTTGTGGATGTACA
GAGTGATATTATTGACACGCCCGGCCGACGGATGGTGATCCCCCTGGCCAGTGCACGTCTGCTGTCAGATAAAGT
CTCCCGTGAACTTTACCCGGTGGTGCATATCGGGGATGAAAGCTGGCGCATGATGACCACCGATATGGCCAGTGT
GCCGGTTTCCGTTATCGGGGAAGAAGTGGCTGATCTCAGCCACCGCGAAAATGACATCAAAAACGCCATTAACCT
GATGTTCTGGGGAATATAAATGTCAGGCTCCCTTATACACAGCCAGTCTGCACCTCGACggtctcAcattggctc
ttcttactAAGGGTGGGCGCGCCGACCCAGCTTTCTTGTACAAAGTTGGCATTATAAGAAAGCATTGCTTATCAA
TTTGTTGCAACGAACAGGTCACTATCAGTCAAAATAAAATCATTATTTGCCATCCAGCTGATATCCCCTATAGTG
AGTCGTATTACATGGTCATAGCTGTTTCCTGGCAGCTCTGGCCCGTGTCTCAAAATCTCTGATGTTACATTGCAC
AAGATAAAAATATATCATCATGAACAATAAAACTGTCTGCTTACATAAACAGTAATACAAGGGGTGTTatgagcc
atattcaacgggaaacgtcgaggccgcgattaaattccaacatggatgctgatttatatgggtataaatgggctc
gcgataatgtcgggcaatcaggtgcgacaatctatcgcttgtatgggaagcccgatgcgccagagttgtttctga
aacatggcaaaggtagcgttgccaatgatgttacagatgagatggtcagactaaactggctgacggaatttatgc
ctcttccgaccatcaagcattttatccgtactcctgatgatgcatggttactcaccactgcgatccccggaaaaa
cagcattccaggtattagaagaatatcctgattcaggtgaaaatattgttgatgcgctggcagtgttcctgcgcc
ggttgcattcgattcctgtttgtaattgtccttttaacagcgatcgcgtatttcgtctcgctcaggcgcaatcac
gaatgaataacggtttggttgatgcgagtgattttgatgacgagcgtaatggctggcctgttgaacaagtctgga
aagaaatgcataaacttttgccattctcaccggattcagtcgtcactcatggtgatttctcacttgataacctta
tttttgacgaggggaaattaataggttgtattgatgttggacgagtcggaatcgcagaccgataccaggatcttg
ccatcctatggaactgcctcggtgagttttctccttcattacagaaacggctttttcaaaaatatggtattgata
atcctgatatgaataaattgcagtttcatttgatgctcgatgagtttttcTAATCAGAATTGGTTAATTGGTTGT
AACACTGGCAGAGCATTACGCTGACTTGACGGGACGGCGCAAGCTCATGACCAAAATCCCTTAACGTGAGTTACG
CGTCGTTCCACTGAGCGTCAGACCCCGTAGAAAAGATCAAAGGATCTTCTTGAGATCCTTTTTTTCTGCGCGTAA
TCTGCTGCTTGCAAACAAAAAAACCACCGCTACCAGCGGTGGTTTGTTTGCCGGATCAAGAGCTACCAACTCTTT
TTCCGAAGGTAACTGGCTTCAGCAGAGCGCAGATACCAAATACTGTCCTTCTAGTGTAGCCGTAGTTAGGCCACC
ACTTCAAGAACTCTGTAGCACCGCCTACATACCTCGCTCTGCTAATCCTGTTACCAGTGGCTGCTGCCAGTGGCG
ATAAGTCGTGTCTTACCGGGTTGGACTCAAGACGATAGTTACCGGATAAGGCGCAGCGGTCGGGCTGAACGGGGG
GTTCGTGCACACAGCCCAGCTTGGAGCGAACGACCTACACCGAACTGAGATACCTACAGCGTGAGCATTGAGAAA
GCGCCACGCTTCCCGAAGGGAGAAAGGCGGACAGGTATCCGGTAAGCGGCAGGGTCGGAACAGGAGAGCGCACGA
GGGAGCTTCCAGGGGGAAACGCCTGGTATCTTTATAGTCCTGTCGGGTTTCGCCACCTCTGACTTGAGCGTCGAT

TTTTGTGATGCTCGTCAGGGGGGCGGAGCCTATGGAAAAACGCCAGCAACGCGGCCTTTTTACGGTTCCTGGCCT
TTTGCTGGCCTTTTGCTCACATGTT

PURPLE/UPPERCASE: M13-F binding site
orange/lowercase: attL1
BLUE/UPPERCASE: *AtMIR390a* 5’ region
RED/UPPERCASE: *Bsa*I site
magenta/lowercase: chloramphenicol resistance gene
MAGENTA/UPPERCASE: *ccd*B gene
red/lowercase: inverted *Bsa*I site
blue/lowercase: *AtMIR390a* 3' region
orange/lowercase/underlined: attL2
PURPLE/UPPERCASE/UNDERLINED: M13-Reverse binding site
brown/lowercase: Kanamycin resistance gene

***>pMDC32B-BS-AtMIR390-B/c* (11629 bp)**

CCAGCCAGCCAACAGCTCCCCGACCGGCAGCTCGGCACAAAATCACCACTCGATACAGGCAGCCCATCAGTCCGG
GACGGCGTCAGCGGGAGAGCCGTTGTAAGGCGGCAGACTTTGCTCATGTTACCGATGCTATTCGGAAGAACGGCA
ACTAAGCTGCCGGGTTTGAAACACGGATGATCTCGCGGAGGGTAGCATGTTGATTGTAACGATGACAGAGCGTTG
CTGCCTGTGATCACCGCGGTTTCAAAATCGGCTCCGTCGATACTATGTTATACGCCAACTTTGAAAACAACTTTG
AAAAAGCTGTTTTCTGGTATTTAAGGTTTTAGAATGCAAGGAACAGTGAATTGGAGTTCGTCTTGTTATAATTAG
CTTCTTGGGGTATCTTTAAATACTGTAGAAAAGAGGAAGGAAATAATAAatggctaaaatgagaatatcaccgga
attgaaaaaactgatcgaaaaataccgctgcgtaaaagatacggaaggaatgtctcctgctaaggtatataagct
ggtgggagaaaatgaaaacctatatttaaaaatgacggacagccggtataaagggaccacctatgatgtggaacg
ggaaaaggacatgatgctatggctggaaggaaagctgcctgttccaaaggtcctgcactttgaacggcatgatgg
ctggagcaatctgctcatgagtgaggccgatggcgtcctttgctcggaagagtatgaagatgaacaaagccctga
aaagattatcgagctgtatgcggagtgcatcaggctctttcactccatcgacatatcggattgtccctatacgaa
tagcttagacagccgcttagccgaattggattacttactgaataacgatctggccgatgtggattgcgaaaactg
ggaagaagacactccatttaaagatccgcgcgagctgtatgatttttaaagacggaaaagcccgaagaggaact
tgtcttttcccacggcgacctgggagacagcaacatctttgtgaaagatggcaaagtaagtggctttattgatct
tgggagaagcggcagggcggacaagtggtatgacattgccttctgcgtccggtcgatcagggaggatatcgggga
agaacagtatgtcgagctatttttgacttactggggatcaagcctgattgggagaaaataaaatattatatttt
actggatgaattgttttagTACCTAGAATGCATGACCAAAATCCCTTAACGTGAGTTTTCGTTCCACTGAGCGTC
AGACCCCGTAGAAAAGATCAAAGGATCTTCTTGAGATCCTTTTTTTCTGCGCGTAATCTGCTGCTTGCAAACAAA
AAAACCACCGCTACCAGCGGTGGTTTGTTTGCCGGATCAAGAGCTACCAACTCTTTTTCCGAAGGTAACTGGCTT
CAGCAGAGCGCAGATACCAAATACTGTCCTTCTAGTGTAGCCGTAGTTAGGCCACCACTTCAAGAACTCTGTAGC
ACCGCCTACATACCTCGCTCTGCTAATCCTGTTACCAGTGGCTGCTGCCAGTGGCGATAAGTCGTGTCTTACCGG
GTTGGACTCAAGACGATAGTTACCGGATAAGGCGCAGCGGTCGGGCTGAACGGGGGGTTCGTGCACACAGCCCAG
CTTGGAGCGAACGACCTACACCGAACTGAGATACCTACAGCGTGAGCTATGAGAAAGCGCCACGCTTCCCGAAGG
GAGAAAGGCGGACAGGTATCCGGTAAGCGGCAGGGTCGGAACAGGAGAGCGCACGAGGGAGCTTCCAGGGGGAAA
CGCCTGGTATCTTTATAGTCCTGTCGGGTTTCGCCACCTCTGACTTGAGCGTCGATTTTTGTGATGCTCGTCAGG
GGGGCGGAGCCTATGGAAAAACGCCAGCAACGCGGCCTTTTTACGGTTCCTGGCCTTTTGCTGGCCTTTTGCTCA
CATGTTCTTTCCTGCGTTATCCCCTGATTCTGTGGATAACCGTATTACCGCCTTTGAGTGAGCTGATACCGCTCG
CCGCAGCCGAACGACCGAGCGCAGCGAGTCAGTGAGCGAGGAAGCGGAAGAGCGCCTGATGCGGTATTTTCTCCT
TACGCATCTGTGCGGTATTTCACACCGCATATGGTGCACTCTCAGTACAATCTGCTCTGATGCCGCATAGTTAAG
CCAGTATACACTCCGCTATCGCTACGTGACTGGGTCATGGCTGCGCCCCGACACCCGCCAACACCCGCTGACGCG
CCCTGACGGGCTTGTCTGCTCCCGGCATCCGCTTACAGACAAGCTGTGACCGTCTCCGGGAGCTGCATGTGTCAG
AGGTTTTCACCGTCATCACCGAAACGCGCGAGGCAGGGTGCCTTGATGTGGGCGCCGGCGGTCGAGTGGCGACGG
CGCGGCTTGTCCGCGCCCTGGTAGATTGCCTGGCCGTAGGCCAGCCATTTTTGAGCGGCCAGCGGCCGCGATAGG
CCGACGCGAAGCGGCGGGGCGTAGGGAGCGCAGCGACCGAAGGGTAGGCGCTTTTTGCAGCTCTTCGGCTGTGCG
CTGGCCAGACAGTTATGCACAGGCCAGGCGGGTTTTAAGAGTTTTAATAAGTTTTAAAGAGTTTTAGGCGGAAAA
ATCGCCTTTTTTCTCTTTTATATCAGTCACTTACATGTGTGACCGGTTCCCAATGTACGGCTTTGGGTTCCCAAT
GTACGGGTTCCGGTTCCCAATGTACGGCTTTGGGTTCCCAATGTACGTGCTATCCACAGGAAAGAGAACTTTTCG
ACCTTTTTCCCCTGCTAGGGCAATTTGCCCTAGCATCTGCTCCGTACATTAGGAACCGGCGGATGCTTCGCCCTC
GATCAGGTTGCGGTAGCGCATGACTAGGATCGGGCCAGCCTGCCCCGCCTCCTCCTTCAAATCGTACTCCGGCAG
GTCATTTGACCCGATCAGCTTGCGCACGGTGAAACAGAACTTCTTGAACTCTCCGGCGCTGCCACTGCGTTCGTA
GATCGTCTTGAACAACCATCTGGCTTCTGCCTTGCCTGCGGCGCGGCGTGCCAGGCGGTAGAGAAAACGGCCGAT
GCCGGGATCGATCAAAAAGTAATCGGGGTGAACCGTCAGCACGTCCGGGTTCTTGCCTTCTGTGATCTCGCGGTA
CATCCAATCAGCTAGCTCGATCTCGATGTACTCCGGCCGCCCGGTTTCGCTCTTTACGATCTTGTAGCGGCTAAT
CAAGGCTTCACCCTCGGATACCGTCACCAGGCGGCCGTTCTTGGCCTTCTTCGTACGCTGCATGGCAACGTGCGT
GGTGTTTAACCGAATGCAGGTTTCTACCAGGTCGTCTTTCTGCTTTCCGCCATCGGCTCGCCGGCAGAACTTGAG
TACGTCCGCAACGTGTGGACGGAACACGCGGCCGGGCTTGTCTCCCTTCCCTTCCCGGTATCGGTTCATGGATTC
GGTTAGATGGGAAACCGCCATCAGTACCAGGTCGTAATCCCACACACTGGCCATGCCGGCCGGCCCTGCGGAAAC
CTCTACGTGCCCGTCTGGAAGCTCGTAGCGGATCACCTCGCCAGCTCGTCGGTCACGCTTCGACAGACGGAAAAC
GGCCACGTCCATGATGCTGCGACTATCGCGGGTGCCCACGTCATAGAGCATCGGAACGAAAAAATCTGGTTGCTC
GTCGCCCTTGGGCGGCTTCCTAATCGACGGCGCACCGGCTGCCGGCGGTTGCCGGGATTCTTTGCGGATTCGATC
AGCGGCCGCTTGCCACGATTCACCGGGGCGTGCTTCTGCCTCGATGCGTTGCCGCTGGGCGGCCTGCGCGGCCTT
CAACTTCTCCACCAGGTCATCACCCAGCGCCGCGCCGATTTGTACCGGGCCGGATGGTTTGCGACCGTCACGCCG
ATTCCTCGGGCTTGGGGGTTCCAGTGCCATTGCAGGGCCGGCAGACAACCCAGCCGCTTACGCCTGGCCAACCGC
CCGTTCCTCCACACATGGGGCATTCCACGGCGTCGGTGCCTGGTTGTTCTTGATTTTCCATGCCGCCTCCTTTAG
CCGCTAAAATTCATCTACTCATTTATTCATTTGCTCATTTACTCTGGTAGCTGCGCGATGTATTCAGATAGCAGC
TCGGTAATGGTCTTGCCTTGGCGTACCGCGTACATCTTCAGCTTGGTGTGATCCTCCGCCGGCAACTGAAAGTTG
ACCCGCTTCATGGCTGGCGTGTCTGCCAGGCTGGCCAACGTTGCAGCCTTGCTGCTGCGTGCGCTCGGACGGCCG
GCACTTAGCGTGTTTGTGCTTTTGCTCATTTTCTCTTTACCTCATTAACTCAAATGAGTTTTGATTTAATTTCAG
CGGCCAGCGCCTGGACCTCGCGGGCAGCGTCGCCCTCGGGTTCTGATTCAAGAACGGTTGTGCCGGCGGCGGCAG
TGCCTGGGTAGCTCACGCGCTGCGTGATACGGGACTCAAGAATGGGCAGCTCGTACCCGGCCAGCGCCTCGGCAA

CCTCACCGCCGATGCGCGTGCCTTTGATCGCCCGCGACACGACAAAGGCCGCTTGTAGCCTTCCATCCGTGACCT
CAATGCGCTGCTTAACCAGCTCCACCAGGTCGGCGGTGGCCCATATGTCGTAAGGGCTTGGCTGCACCGGAATCA
GCACGAAGTCGGCTGCCTTGATCGCGGACACAGCCAAGTCCGCCGCCTGGGGCGCTCCGTCGATCACTACGAAGT
CGCGCCGGCCGATGGCCTTCACGTCGCGGTCAATCGTCGGGCGGTCGATGCCGACAACGGTTAGCGGTTGATCTT
CCCGCACGGCCGCCCAATCGCGGGCACTGCCCTGGGGATCGGAATCGACTAACAGAACATCGGCCCCGGCGAGTT
GCAGGGCGCGGGCTAGATGGGTTGCGATGGTCGTCTTGCCTGACCCGCCTTTCTGGTTAAGTACAGCGATAACCT
TCATGCGTTCCCCTTGCGTATTTGTTTATTTACTCATCGCATCATATACGCAGCGACCGCATGACGCAAGCTGTT
TTACTCAAATACACATCACCTTTTTAGACGGCGGCGCTCGGTTTCTTCAGCGGCCAAGCTGGCCGGCCAGGCCGC
CAGCTTGGCATCAGACAAACCGGCCAGGATTTCATGCAGCCGCACGGTTGAGACGTGCGCGGGCGGCTCGAACAC
GTACCCGGCCGCGATCATCTCCGCCTCGATCTCTTCGGTAATGAAAAACGGTTCGTCCTGGCCGTCCTGGTGCGG
TTTCATGCTTGTTCCTCTTGGCGTTCATTCTCGGCGGCCGCCAGGGCGTCGGCCTCGGTCAATGCGTCCTCACGG
AAGGCACCGCGCCGCCTGGCCTCGGTGGGCGTCACTTCCTCGCTGCGCTCAAGTGCGCGGTACAGGGTCGAGCGA
TGCACGCCAAGCAGTGCAGCCGCCTCTTTCACGGTGCGGCCTTCCTGGTCGATCAGCTCGCGGGCGTGCGCGATC
TGTGCCGGGGTGAGGGTAGGGCGGGGGCCAAACTTCACGCCTCGGGCCTTGGCGGCCTCGCGCCCGCTCCGGGTG
CGGTCGATGATTAGGGAACGCTCGAACTCGGCAATGCCGGCGAACACGGTCAACACCATGCGGCCGGCCGGCGTG
GTGGTGTCGGCCCACGGCTCTGCCAGGCTACGCAGGCCCGCGCCGGCCTCCTGGATGCGCTCGGCAATGTCCAGT
AGGTCGCGGGTGCTGCGGGCCAGGCGGTCTAGCCTGGTCACTGTCACAACGTCGCCAGGGCGTAGGTGGTCAAGC
ATCCTGGCCAGCTCCGGGCGGTCGCGCCTGGTGCCGGTGATCTTCTCGGAAAACAGCTTGGTGCAGCCGGCCGCG
TGCAGTTCGGCCCGTTGGTTGGTCAAGTCCTGGTCGTCGGTGCTGACGCGGGCATAGCCCAGCAGGCCAGCGGCG
GCGCTCTTGTTCATGGCGTAATGTCTCCGGTTCTAGTCGCAAGTATTCTACTTTATGCGACTAAAACACGCGACA
AGAAAACGCCAGGAAAAGGGCAGGGCGGCAGCCTGTCGCGTAACTTAGGACTTGTGCGACATGTCGTTTTCAGAA
GACGGCTGCACTGAACGTCAGAAGCCGACTGCACTATAGCAGCGGAGGGGTTGGATCAAAGTACTTTGATCCCGA
GGGGAACCCTGTGGTTGGCATGCACATACAAATGGACGAACGGATAAACCTTTTCACGCCCTTTTAAATATCCGT
TATTCTAATAAACGCTCTTTTCTCTTAGGtttacccgccaatatatcctgtcaAACACTGATAGTTTAAACTGAA
GGCGGGAAACGACAATCTGATCCAAGCTCAAGCTGCTCTAGCATTCGCCATTCAGGCTGCGCAACTGTTGGGAAG
GGCGATCGGTGCGGGCCTCTTCGCTATTACGCCAGCTGGCGAAAGGGGGATGTGCTGCAAGGCGATTAAGTTGGG
TAACGCCAGGGTTTTCCCAGTCACGACGTTGTAAAACGACGGCCAGTGCCAAGCTTGGCGTGCCTGCAGGTCAAC
ATGGTGGAGCACGACACACTTGTCTACTCCAAAAATATCAAAGATACAGTCTCAGAAGACCAAAGGGCAATTGAG
ACTTTTCAACAAAGGGTAATATCCGGAAACCTCCTCGGATTCCATTGCCCAGCTATCTGTCACTTTATTGTGAAG
ATAGTGGAAAAGGAAGGTGGCTCCTACAAATGCCATCATTGCGATAAAGGAAAGGCCATCGTTGAAGATGCCTCT
GCCGACAGTGGTCCCAAAGATGGACCCCCACCCACGAGGAGCATCGTGGAAAAAGAAGACGTTCCAACCACGTCT
TCAAAGCAAGTGGATTGATGTGATAACATGGTGGAGCACGACACACTTGTCTACTCCAAAAATATCAAAGATACA
GTCTCAGAAGACCAAAGGGCAATTGAGACTTTTCAACAAAGGGTAATATCCGGAAACCTCCTCGGATTCCATTGC
CCAGCTATCTGTCACTTTATTGTGAAGATAGTGGAAAAGGAAGGTGGCTCCTACAAATGCCATCATTGCGATAAA
GGAAAGGCCATCGTTGAAGATGCCTCTGCCGACAGTGGTCCCAAAGATGGACCCCCACCCACGAGGAGCATCGTG
GAAAAAGAAGACGTTCCAACCACGTCTTCAAAGCAAGTGGATTGATGTGATATCTCCACTGACGTAAGGGATGAC
GCACAATCCCACTATCCTTCGCAAGACCCTTCCTCTATATAAGGAAGTTCATTTCATTTGGAGAGGACCTCGACT
CTAGAGGATCCCCGGGTACCGGGCCCCCCCTCGAGGCGCGCCAAGCTATCAAACAAGTTTGTACAAAAAAGCAGG
CTCCGCGGCCGCCCCCTTCACCAGTAGAGAAGAATCTGTAAGAGACCATTAGGCACCCCAGGCTTTACACTTTAT
GCTTCCGGCTCGTATAATGTGTGGATTTTGAGTTAGGAGCCGTCGAGATTTTCAGGAGCTAAGGAAGCTAAAatg
gagaaaaaaatcactggatataccaccgttgatatatcccaatggcatcgtaaagaacattttgaggcatttcag
tcagttgctcaatgtacctataaccagaccgttcagctggatattacggcctttttaaagaccgtaaagaaaaat
aagcacaagttttatccggcctttattcacattcttgcccgcctgatgaatgctcatccggagttccgtatggca
atgaaagacggtgagctggtgatatgggatagtgttcacccttgttacaccgttttccatgagcaaactgaaacg
ttttcatcgctctggagtgaataccacgacgatttccggcagtttctacacatatattcgcaagatgtggcgtgt
tacggtgaaaacctggcctatttccctaaagggtttattgagaatatgtttttcgtctcagccaatccctgggtg
agtttcaccagttttgatttaaacgtggccaatatggacaacttcttcgcccccgttttcaccatgggcaaatat
tatacgcaaggcgacaaggtgctgatgccgctggcgattcaggttcatcatgccgtttgtgatggcttccatgtc
ggcagaatgcttaatgaattacaacagtactgcgatgagtggcagggcggggcgtaaACGCGTGGAGCCGGCTTA
CTAAAAGCCAGATAACAGTATGCGTATTTGCGCGCTGATTTTTGCGGTATAAGAATATATACTGATATGTATACC
CGAAGTATGTCAAAAAGAGGTATGCTATGAAGCAGCGTATTACAGTGACAGTTGACAGCGACAGCTATCAGTTGC
TCAAGGCATATATGATGTCAATATCTCCGGTCTGGTAAGCACAACCATGCAGAATGAAGCCCGTCGTCTGCGTGC
CGAACGCTGGAAAGCGGAAAATCAGGAAGGGATGGCTGAGGTCGCCCGGTTTATTGAAATGAACGGCTCTTTTGC
TGACGAGAACAGGGGCTGGTGAAATGCAGTTTAAGGTTTACACCTATAAAAGAGAGAGCCGTTATCGTCTGTTTG
TGGATGTACAGAGTGATATTATTGACACGCCCGGCCGACGGATGGTGATCCCCCTGGCCAGTGCACGTCTGCTGT
CAGATAAAGTCTCCCGTGAACTTTACCCGGTGGTGCATATCGGGGATGAAAGCTGGCGCATGATGACCACCGATA
TGGCCAGTGTGCCGGTTTCCGTTATCGGGGAAGAAGTGGCTGATCTCAGCCACCGCGAAAATGACATCAAAAACG
CCATTAACCTGATGTTCTGGGGAATATAAATGTCAGGCTCCCTTATACACAGCCAGTCTGCACCTCGACggtctc
AcattggctcttcttactAAGGGTGGGCGCGCCGACCCAGCTTTCTTGTACAAAGTGGTTCGATAATTCCTTAAT
TAACTAGTTCTAGAGCGGCCGCCCACCGCGGTGGAGCTCGAATTTCCCCGATCGTTCAAACATTTGGCAATAAAG
TTTCTTAAGATTGAATCCTGTTGCCGGTCTTGCGATGATTATCATATAATTTCTGTTGAATTACGTTAAGCATGT

AATAATTAACATGTAATGCATGACGTTATTTATGAGATGGGTTTTTATGATTAGAGTCCCGCAATTATACATTTA
ATACGCGATAGAAAACAAAATATAGCGCGCAAACTAGGATAAATTATCGCGCGCGGTGTCATCTATGTTACTGAA
TTCGTAATCATGGTCATAGCTGTTTCCTGTGTGAAATTGTTATCCGCTCACAATTCCACACAACATACGAGCCGG
AAGCATAAAGTGTAAAGCCTGGGGTGCCTAATGAGTGAGCTAACTCACATTAATTGCGTTGCGCTCACTGCCCGC
TTTCCAGTCGGGAAACCTGTCGTGCCAGCTGCATTAATGAATCGGCCAACGCGCGGGGAGAGGCGGTTTGCGTAT
TGGCTAGAGCAGCTTGCCAACATGGTGGAGCACGACACTCTCGTCTACTCCAAGAATATCAAAGATACAGTCTCA
GAAGACCAAAGGGCTATTGAGACTTTTCAACAAAGGGTAATATCGGGAAACCTCCTCGGATTCCATTGCCCAGCT
ATCTGTCACTTCATCAAAAGGACAGTAGAAAAGGAAGGTGGCACCTACAAATGCCATCATTGCGATAAAGGAAAG
GCTATCGTTCAAGATGCCTCTGCCGACAGTGGTCCCAAAGATGGACCCCCACCCACGAGGAGCATCGTGGAAAAA
GAAGACGTTCCAACCACGTCTTCAAAGCAAGTGGATTGATGTGATAACatggtggagcacgacactctcgtctac
tccaagaatatcaaagatacagtctcagaagaccaaagggctattgagacttttcaacaaagggtaatatcggga
aacctcctcggattccattgcccagctatctgtcacttcatcaaaaggacagtagaaaaggaaggtggcacctac
aaatgccatcattgcgataaaggaaaggctatcgttcaagatgcctctgccgacagtggtcccaaagatggaccc
ccacccacgaggagcatcgtggaaaaagaagacgttccaaccacgtcttcaaagcaagtggattgatgtgatatc
tccactgacgtaagggatgacgcacaatcccactatccttcgcaagaccttcctctatataaggaagttcatttc
atttggagaggACACGCTGAAATCACCAGTCTCTCTCTACAAATCTATCTCTCTCGAGCTTTCGCAGATCCCGGG
GGGCAATGAGATATGAAAAAGCCTGAACTCACCGCGACGTCTGTCGAGAAGTTTCTGATCGAAAAGTTCGACAGC
GTCTCCGACCTGATGCAGCTCTCGGAGGGCGAAGAATCTCGTGCTTTCAGCTTCGATGTAGGAGGGCGTGGATAT
GTCCTGCGGGTAAATAGCTGCGCCGATGGTTTCTACAAAGATCGTTATGTTTATCGGCACTTTGCATCGGCCGCG
CTCCCGATTCCGGAAGTGCTTGACATTGGGGAGTTTAGCGAGAGCCTGACCTATTGCATCTCCCGCCGTGCACAG
GGTGTCACGTTGCAAGACCTGCCTGAAACCGAACTGCCCGCTGTTCTACAACCGGTCGCGGAGGCTATGGATGCG
ATCGCTGCGGCCGATCTTAGCCAGACGAGCGGGTTCGGCCCATTCGGACCGCAAGGAATCGGTCAATACACTACA
TGGCGTGATTTCATATGCGCGATTGCTGATCCCCATGTGTATCACTGGCAAACTGTGATGGACGACACCGTCAGT
GCGTCCGTCGCGCAGGCTCTCGATGAGCTGATGCTTTGGGCCGAGGACTGCCCCGAAGTCCGGCACCTCGTGCAC
GCGGATTTCGGCTCCAACAATGTCCTGACGGACAATGGCCGCATAACAGCGGTCATTGACTGGAGCGAGGCGATG
TTCGGGGATTCCCAATACGAGGTCGCCAACATCTTCTTCTGGAGGCCGTGGTTGGCTTGTATGGAGCAGCAGACG
CGCTACTTCGAGCGGAGGCATCCGGAGCTTGCAGGATCGCCACGACTCCGGGCGTATATGCTCCGCATTGGTCTT
GACCAACTCTATCAGAGCTTGGTTGACGGCAATTTCGATGATGCAGCTTGGGCGCAGGGTCGATGCGACGCAATC
GTCCGATCCGGAGCCGGGACTGTCGGGCGTACACAAATCGCCCGCAGAAGCGCGGCCGTCTGGACCGATGGCTGT
GTAGAAGTACTCGCCGATAGTGGAAACCGACGCCCCAGCACTCGTCCGAGGGCAAAGAAATAGAGTAGATGCCGA
CCGGATCTGTCGATCGACAAGCTCGAGtttctccataataatgtgtgagtagttcccagataagggaattagggt
tcctatagggtttcgctcatgtgttgagcatataagaaacccttagtatgtatttgtatttgtaaaatacttcta
tcaataaaatttctaattcctaaaaccaaaatccagtactaaaatccagatcCCCCGAATTAATTCGGCGTTAAT
TCAGTACATTAAAAACGTCCGCAATGTGTTATTAAGTTGTCTAAGCGTCAATTTGTTTACACCACAATATATCCT
GCCA

brown/lowercase: kanamycin resistance gene
CYAN/UPPERCASE/UNDERLINED: C->A transversion to block vector's *Bsa*I site
cyan/lowercase: T-DNA right border
GREEN/UPPERCASE: 2x35S CaMV promoter
ORANGE/UPPERCASE: attB1
BLUE/UPPERCASE: *AtMIR390a* 5' region
RED/UPPERCASE: *Bsa*I site
magenta/lowercase: chloramphenicol resistance gene
MAGENTA/UPPERCASE: *ccd*B gene
red/lowercase: inverted *Bsa*I site
blue/lowercase: *AtMIR390a* 3' region
ORANGE/UPPERCASE/UNDERLINED: attB2
GREY/UPPERCASE/UNDERLINED: Nos terminator
green/lowercase: CaMV promoter
BROWN/UPPERCASE: hygromycin resistance gene
green/lowercase/underlined: CaMV terminator
CYAN/UPPERCASE: T-DNA left border
